# Landscape of enhancer disruption and functional screen in melanoma cells

**DOI:** 10.1186/s13059-023-03087-5

**Published:** 2023-10-30

**Authors:** Zhao Wang, Menghan Luo, Qian Liang, Ke Zhao, Yuelin Hu, Wei Wang, Xiangling Feng, Bolang Hu, Jianjin Teng, Tianyi You, Ran Li, Zhengkai Bao, Wenhao Pan, Tielong Yang, Chao Zhang, Ting Li, Xiaobao Dong, Xianfu Yi, Ben Liu, Li Zhao, Miaoxin Li, Kexin Chen, Weihong Song, Jilong Yang, Mulin Jun Li

**Affiliations:** 1 Oujiang Laboratory (Zhejiang Lab for Regenerative Medicine, Vision and Brain Health), Institute of Aging, Key Laboratory of Alzheimer’s Disease of Zhejiang Province, The Second Affiliated Hospital, Wenzhou Medical University, Wenzhou, China; 2 Department of Epidemiology and Biostatistics, Tianjin Key Laboratory of Molecular Cancer Epidemiology, The Province and Ministry Co-Sponsored Collaborative Innovation Center for Medical Epigenetics, National Clinical Research Center for Cancer, Tianjin Medical University Cancer Institute and Hospital, Tianjin Medical University, Tianjin, China; 3 Department of Bioinformatics, School of Basic Medical Sciences, Tianjin Medical University, Tianjin, China; 4 Scientific Research Center, Wenzhou Medical University, Wenzhou, China; 5 Department of Bone and Soft Tissue Tumor, Tianjin Medical University Cancer Institute and Hospital, Tianjin Medical University, Tianjin, China; 6 Department of Biochemistry and Molecular Biology, School of Basic Medical Sciences, Tianjin Medical University, Tianjin, China; 7 Zhongshan School of Medicine, Sun Yat-Sen University, Guangzhou, China

**Keywords:** Melanoma, Highly recurrent regions (HRRs), Enhancer, Myocyte enhancer factor 2A （*MEF2A）*, Phosphatase and tensin homolog (*PTEN*)

## Abstract

**Background:**

The high mutation rate throughout the entire melanoma genome presents a major challenge in stratifying true driver events from the background mutations. Numerous recurrent non-coding alterations, such as those in enhancers, can shape tumor evolution, thereby emphasizing the importance in systematically deciphering enhancer disruptions in melanoma.

**Results:**

Here, we leveraged 297 melanoma whole-genome sequencing samples to prioritize highly recurrent regions. By performing a genome-scale CRISPR interference (CRISPRi) screen on highly recurrent region-associated enhancers in melanoma cells, we identified 66 significant hits which could have tumor-suppressive roles. These functional enhancers show unique mutational patterns independent of classical significantly mutated genes in melanoma. Target gene analysis for the essential enhancers reveal many known and hidden mechanisms underlying melanoma growth. Utilizing extensive functional validation experiments, we demonstrate that a super enhancer element could modulate melanoma cell proliferation by targeting *MEF2A*, and another distal enhancer is able to sustain *PTEN* tumor-suppressive potential via long-range interactions.

**Conclusions:**

Our study establishes a catalogue of crucial enhancers and their target genes in melanoma growth and progression, and illuminates the identification of novel mechanisms of dysregulation for melanoma driver genes and new therapeutic targeting strategies.

**Supplementary Information:**

The online version contains supplementary material available at 10.1186/s13059-023-03087-5.

## Background

Melanoma, one of the most aggressive and malignant tumors, has a high fatality rate with over fifty thousand deaths reported each year worldwide [1]. Previous large-scale genome sequencing studies have identified many putative cancer genes and hotspots in different types of melanomas [2–8]. However, about 15% cutaneous melanoma and over 50% mucosal/acral melanoma patients are unclassified based on the pattern of the most prevalent significantly mutated genes, including *BRAF*, *RAS*, and *NF1* [2, 3]. Few studies have systematically characterized genomic events outside of protein-coding genes and their functions in melanoma development. The major challenge comes from the predominantly high mutation burden of the melanoma genome with a C > T nucleotide transition signature attributable to ultraviolet radiation [9]. This hampers the identification of true melanoma drivers from background mutations only relying on genome sequencing and computational modelling, especially in the non-coding genomic region [10].

Given the poorly understood localized hypermutation processes, establishing and elucidating the melanoma regulatory landscape, such as enhancer disruption, could provide an effective avenue for identification of non-coding drivers [11–13]. It has been demonstrated that enhancer signatures are strongly enriched for SOX10/MITF and AP-1/TEAD regulome in melanoma [14–17]. Furthermore, KMT2D or STAG2 mutant melanomas could rewire metabolic pathways or interferon signaling through enhancer reprogramming [18, 19]. Importantly, CRISPR-based screening of *cis*-regulatory elements (CREs) provides a powerful tool to comprehensively investigate functional mutation events in the non-coding genome of cancers [20–22]. A high-resolution CRISPR screen of non-coding functional elements surrounding three genes, including *NF1*, *NF2*, and *CUL3*, uncovered several critical CREs that modulate drug resistance in melanoma [23]. These studies highlighted the importance of CRE dysregulations during melanoma tumorigenesis and emphasized the necessity of systematic deciphering of enhancer-altered events in melanoma genomes.

**Fig. 1:**
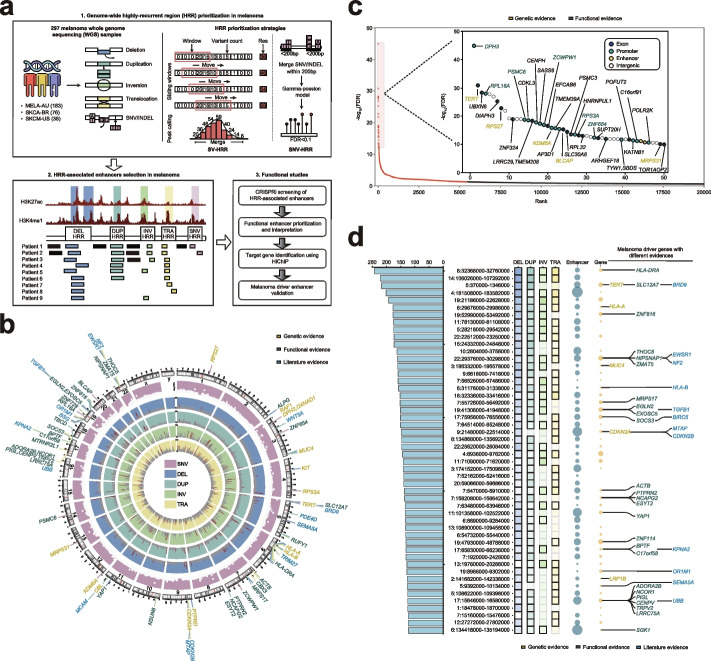
Genome-wide identification and prioritization of highly recurrent regions (HRRs) based on 297 melanoma WGS data. **a** Schematic view of HRR detection strategy and functional enhancer screening in this study. **b** Circos diagram summarizing the full HRR content of melanoma (SNV: single-nucleotide variant, DEL: deletion, DUP: duplication, INV: inversion, and TRA: translocations). Colors of the tracks and label links are determined by mutation types, only genes with most highly recurrence mutation and driver evidence are labeled, genes are divided by color according to different evidence sources (yellow: genetic evidence, candidate melanoma driver genes with genetic evidence carrying significant mutations which were integrated from several large-scale sequencing studies and reviews [2, 3, 10]; green: functional evidence, melanoma essential genes with functional evidence which were collected from The Cancer Dependency Map (DepMap) [24, 25]; blue: literature evidence, putative melanoma cancer genes which were compiled from public cancer gene databases including CancerMine [26], IntOGen [27], and NCG [28]). **c** Top 50 significant SNV/INDEL-HRRs after correcting background mutations and covariates, genes are labeled according to genetic and functional evidence. Also, dots were filled by different color based on genomic attributes of HRRs (exon: blue; promoter: light green; enhancer: yellow). **d** Genomic features of the top 50 prioritized SV-HRRs, bar plot represents total recurrence of each HRR, the heatmap provides the genomic location (GRCh37/hg19), types of HRRs as well as their recurrence. Melanoma essential genes in the right panel are present according to different evidence sources

By systematically integrating 297 high-coverage melanoma whole-genome sequencing (WGS) datasets, we prioritized highly recurrent regions (HRRs) across the whole melanoma genome. We designed a genome-scale CRISPR interference (CRISPRi) screening and identified 66 significant HRR-associated enhancers that could play growth-suppressive roles in melanoma cells. Target gene analysis for these essential enhancers revealed many hidden mechanisms underlying melanoma growth, with others already confirmed. We further demonstrated that two high-level screened HRR-associated enhancers are crucial in sustaining *MEF2A* and *PTEN* tumor-suppressive potential in melanoma cells (Fig. 1a).

## Results

### Genome-wide prioritization of highly recurrent regions (HRRs) in 297 melanoma WGS datasets

To gain sufficient power in detection of recurrent alterations in the melanoma non-coding genome, we comprehensively integrated 297 melanoma genomes with paired (tumor and normal tissues) and high-coverage (> 30 ×) WGS data from three different projects, deposited in the International Cancer Genome Consortium (ICGC) [29]. This included SKCM-US (38 patients), MELA-AU (183 patients), and SKCA-BR (76 patients) (Additional file 2: Table S1). Point somatic mutations including single-nucleotide variants (SNVs) and insertions/deletions (INDELs) were uniformly identified via GATK Mutect2 [30] (Additional file 1: Fig. S1a). To increase detection sensitivity for structural variant (SV) calling, we used two robust somatic SV detection tools, Manta [31] and GRIDSS [32], to identify four types of simple somatic SV, including deletions (DELs), duplications (DUPs), inversions (INVs), and translocations (TRAs) (Additional file 1: Fig. S1b). Based on these mutation spectra from 297 melanoma samples, we applied a Gamma-Poisson model [33] to test genomic intervals showing an excess of somatic SNVs/INDELs (Additional file 1: Fig. S1c) and leveraged a sliding window strategy to prioritize the recurrence of somatic SVs among melanoma patients (Additional file 1: Fig. S1d) (see “Methods” for details).

Consequently, we identified 5508 SNV/INDEL-associated HRRs (SNV/INDEL-HRRs) (FDR < 5%) and 13,736 SV-associated HRRs (SV-HRRs) (≥ 10 donors) in the melanoma genomes, including 8265 DEL-HRRs, 3961 DUP-HRRs, 1224 INV-HRRs, and 2717 TRA-HRRs (Fig. 1b, Additional file 3: Table S2, Additional file 4: Table S3). We prioritized these HRRs according to their mutation recurrence frequency and found that over half of the high priority HRRs incorporate known melanoma driver genes revealed by genomic analysis, CRISPR fitness screening, and other literature evidence (Fig. 1b). Among SNV/INDEL-HRRs, our results showed that *DPH3, TERT*, *UBXN8*, *RPL18A*, *PSMC6* promoter, and *RPS27, SASS6, MRPS31* 5’UTR were significantly mutated, which is consistent with previous WGS studies [3] (Fig. 1c, Additional file 3: Table S2). Notably, about 66% of the high priority SNV/INDEL-HRRs were located in the non-coding region, suggesting that alteration of the regulatory code could play an essential role in melanoma growth. Besides, 72 and 46% melanoma driver genes were affected by DEL and DUP, respectively, but few were influenced by other types of SV. These include some canonical melanoma tumor-suppressive genes such as *BRD9* [34, 35] and *CDKN2A/CDKN2B* [36, 37], and several critical melanoma oncogenes like *BIRC5* [38] and *YAP1* [39]. Nevertheless, the majority of SV-HRRs occurred at the non-coding region with unknown function (Fig. 1d, Additional file 4: Table S3).

### CRISPR tiling screen of HRR-associated enhancers for melanoma cell growth

**Fig. 2:**
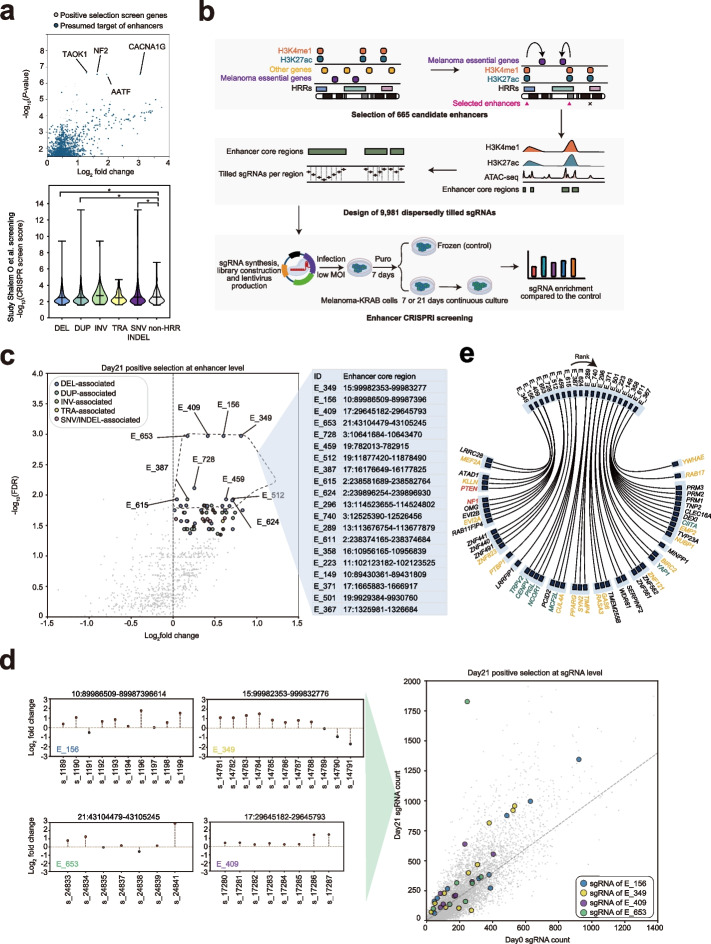
Functional screen of HRR-associated enhancers in melanoma. **a** Functional evaluation of HRR-associated enhancer in A375 public CRISPR KO positive selection screen. Scatter plot represents the most significant presumed target genes of HRR-associated enhancers in CRISPR positive selection results, presumed targets were defined as genes within 1 Mb upstream and downstream of enhancers; box plot represents the difference of CRISPR enrichment scores between the presumed genes in HRR-associated enhancer across 5 HRR types group and those in HRR-unrelated enhancer group, Mann–Whitney *U* test (*p*-value DEL = 0.0033; *p*-value DUP = 0.0028; *p*-value SNV = 0.0076). **b** Pipeline for selecting HRR-associated enhancer core regions, CRISPRi sgRNA library design and screening workflow. **c** CRISPRi screen result at enhancer level, a total of 66 functional enhancers with FDR < 0.05 were highlighted. Details about the top 20 enhancers are shown on the right table. **d** Annotation of candidate target genes of the functional enhancers according to H3K27ac HiChIP loops, genes are labeled by colors according to different evidence sources (purple: genetic evidence, green: functional evidence, blue: literature evidence, and yellow: other cancer driver genes, annotation resource see “Methods” for details). **e** CRISPRi screen result at sgRNA level, the sgRNA enrichment diagrams were shown for the top 4 significant enhancers

Given the highest mutation frequency and distinct mutational processes of melanoma compared with other cancer types, identification of true non-coding drivers from these candidate melanoma HRRs remains a challenge. Many research studies have supported that enhancer elements are highly mutated in cancers and constantly shaped via tumor evolutionary selection [11, 40–42]. Thus, we applied publicly available melanoma H3K27ac ChIP-seq to define active enhancers and intersected them with our identified melanoma HRRs (see “Methods” for details). We uncovered 4645 HRR-associated enhancers across the melanoma genome. To explore whether HRR-associated enhancers play a potential role in melanoma growth and progression, we aligned all active melanoma enhancers with their potential target genes (1 Mb upstream and downstream). Their tumor proliferation potential was then evaluated based on previous CRISPR knockout (KO) screens in melanoma [43–45]. Our analysis revealed that genes associated with DEL- and DUP-associated enhancers elicited more significant signals in the positive/negative selection of the CRISPR KO screens than those linked to HRR-unrelated enhancers (*p*-value < 0.05, Mann–Whitney *U* test) (Fig. 2a, Additional file 1: Fig. S2a). This implies that enhancer disruption through recurrent mutation trends to modulate genes suppressive of melanoma proliferation/survival.

To systematically inspect the functional consequence of enhancer disruption within melanoma HRRs, we performed a CRISPR interference (CRISPRi) screen on the A375 human melanoma cell line by employing the dCas9-KRAB system and dispersedly tiled sgRNAs for each of selected HRR-associated enhancers. Specifically, to reduce the library size and improve screening power due to the abundant enhancers, we initially collected 190 essential candidate genes associated with melanoma cell growth and drug resistance from published CRISPR/shRNA screening studies (Additional file 5: Table S4). We selected 665 HRR-associated enhancers harboring both H3K27ac and H3K4me1 signals in A375 cells within 1 Mb region of candidate essential genes. Since many H3K27ac and H3K4me1 peaks span a long distance, we also tailored enhancer regions with chromatin accessibility signal based on ATAC-seq peaks, yielding 805 enhancer core regions. Finally, we designed a pooled sgRNA library (total 9981 sgRNAs, including 1000 non-targeting control sgRNAs) via an intersected tiling strategy, in which each selected enhancer core region received a mean of 11 sgRNAs (range 2–34) (Fig. 2b, Additional file 6: Table S5). During screening, we generated melanoma cell lines stably expressing CRISPRi transgenes and performed lentiviral transduction of pooled sgRNAs at low multiplicity of infection (MOI) = 0.2. The transduced cells were selected using puromycin for a week and subsequently cultured for 7 or 21 days before harvesting the genomic DNA. The sgRNA sequences were PCR-amplified and sequenced to determine enrichment at day 7 or 21 relative to day 0 cells (Fig. 2b).

We first performed two independent CRISPRi screens on A375 (BRAF^V600E^ melanoma cell line) cell proliferation with the pooled sgRNA library (Additional file 7: Table S6). The MAGeCK-RRA algorithm [46] was used to analyze the positively selected enhancers (see “Methods” for details). The results from the replicate screens were highly consistent (*r* > 0.9, Spearman’s correlation test) and the non-targeting control showed limited effect on cell viability (Additional file 1: Fig. S2b). By positive selection analysis of CRISPRi screening at day 21, we revealed 66 significant enhancers (false discovery rate (FDR) < 0.05) which displayed a strong pro-proliferative effect (Fig. 2c, Additional file 1: Fig. S2c, Additional file 8: Table S7), suggesting these enhancers could function as tumor-suppressive regulatory elements in melanoma growth. Analysis of tiled sgRNA enrichment for the top four functional enhancers, including E_349, E_156, E_409, and E_653, further demonstrated that the enhancer effect on tumor growth can be robustly estimated (Fig. 2d). To delineate the potential target genes for the 66 significant HRR-associated enhancers, we performed in situ Hi-C followed by chromatin immunoprecipitation (HiChIP) of enhancer mark H3K27ac on A375 cells (Fig. 2e). Several target genes of top hits are regarded as classical melanoma tumor suppressors, such as *PTEN* and *NF1*. These enhancers are associated with highly recurrent loss-of-function events in melanoma patients, which implies that tumors may acquire survival advantages by disrupting the genomic sequence of these enhancers, thus decreasing the expression level of their tumor suppressor targets.

To explore the stability of the CRISPRi screening at different time points, we compared positive selection results between day 21 and day 7 screens (Additional file 1: Fig. S3a, c; Additional file 7: Table S6). We found that 14 of 66 hits from the A375 day 21 screen were also enriched on day7, including top-performing enhancer E_349. Such moderate overlap may suggest a premature screen at day7 or potential background amplification at day21. To investigate cell-type specificity of functionally enriched enhancers, we performed CRISPRi screening on a BRAF wild-type melanoma cell line, SK-MEL-2. In the positive selection from SK-MEL-2 day7 screen, we identified 25 significant enhancers, with 19 shared by either A375 day7 or day21 screens. Notably, another high-performing enhancer, E_156, showed enrichment in SK-MEL-2 cells, meriting further investigation (Additional file 8: Table S7). Additionally, we analyzed negative selection from CRISPRi screens on A375 and SK-MEL-2 (Additional file 1: Fig. S3b, d). Day7 screening revealed more functional drop-out enhancers, but fewer were detected in the day21 screen, with nearly half being of DUP type (Additional file 8: Table S7). Interestingly, enhancer E_763, potentially targeting the classic melanoma driver gene *MITF*, was supported by our HiChIP data.

### Genomic and epigenomic features of functional enhancers in melanoma

**Fig. 3:**
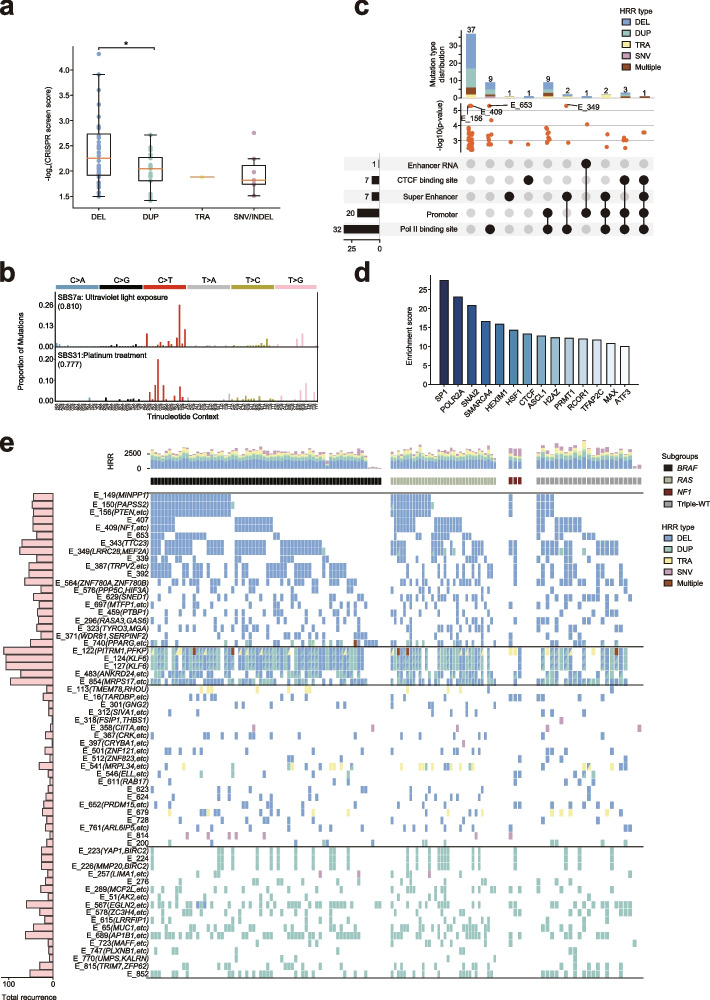
Genetic and epigenetic characteristics of melanoma functional enhancers. **a** Distribution of the functional enhancers in different HRR types, and DEL-HRRs showed a stronger enrichment in the CRISPRi screening, *p*-value = 0.036, Mann–Whitney *U* test. **b** De novo mutational signature analysis of functional HRR-associated enhancers identified two predominant signatures corresponding to ultraviolet light exposure (SBS7a) and platinum treatment (SBS31). Cosine similarity values with COSMIC mutational signatures are shown. **c** Epigenetic features of the functional enhancers in melanoma, the upset plot represents the distribution of five epigenetic signals on the functional enhancers, the bar plot shows driver mutation types, and CRISPRi screen *p*-values were labeled for top enhancers. **d** Colocalized transcription factors at the functional enhancer regions. **e** Mutational spectrum and molecular classification for genomic loci of the functional enhancers based on WGS somatic mutation profiles of 137 MELA-AU cutaneous melanoma samples. The overall recurrence rate of enhancer-located HRR is shown on the side bar, the number of HRRs per patient is shown on the top bar graph, and the functional enhancers and the target genes support by HiChIP are shown on the left. Enhancers are clustered according to their HRR types, and patients are grouped based on previous molecular classification (mutant *BRAF*, mutant *RAS*, mutant *NF1*, and Triple-WT) defined by TCGA

To investigate the potential tumor-suppressive mechanism and clinical significance of HRR-associated enhancers characterized from the A375 CRISPRi positive selection screen, we initially analyzed the recurrent mutation pattern around the 66 significant HRR-associated enhancers. As expected, inhibition of enhancers associated with DEL-HRR showed a stronger growth-promoting effect of tumor than perturbation of enhancers linked to other types of HRR (Fig. 3a). This indicates that these DEL events may drive cancer progression through disruption of tumor-suppressive enhancers. To inspect the mechanism involved in these functional enhancers, we extracted single-base-pair substitutions (SBS) in enhancer regions and calculated de novo mutational signatures using Palimpsest [47]. By examining the COSMIC mutational signature database [48], we identified two predominant signatures corresponding to ultraviolet light exposure (SBS7a) and platinum treatment (SBS31) (Fig. 3b). Analysis on all 4645 HRR-associated enhancers and separate significant enhancers also support this finding (Additional file 1: Fig. S4a, b). Thus, consistent with highly mutated protein-coding genes, exposure to ultraviolet light is still a major cause of enhancer hypermutation in melanoma. To exploit the epigenomic features associated with the 66 significant enhancers, we collected A375 ChIP-seq for some relevant transcription factors (TFs) and histone modifications (including Pol II, CTCF, MITF, H3K27ac, H3K4me3), and PRO-seq for measuring nascent RNAs. By inspecting the preference of the aforementioned loci at these functional enhancers (Additional file 9: Table S8), we found that most of the enhancers harbor the Pol II binding signal and nearly half of them show promoter-like function. In addition, 10% of enhancers display a super enhancer signature, including the most significant ones E_349 and E_740 (Fig. 3c). Among the signals from the 13 melanoma essential TFs, the ChIP-seq results suggested that the classical melanoma driver TF, MITF, might not be associated with these enhancers (Additional file 1: Fig. S4c). However, we observed HEXIM1 binding signals present in 27 functional enhancer regions, including top hits E_349 and E_156. Additionally, we discovered that binding signals from AP2A, AR, and EGFR were present on many enhancers (Additional file 1: Fig. S4c). In addition, we analyzed the enrichment of transcription regulators with the 66 significant enhancers and found that several critical melanoma-associated TFs (e.g., SP1, SNAI2, HEXIM1, ASCL1) preferentially bind at these enhancer loci (Fig. 3d). Consistent with our result, SP1 and HEXIM1 have been documented to play an important regulatory role in inhibiting melanoma-specific gene transcription. The melanoma tumor suppressor gene *HEXIM1* is upregulated by obligatory binding of SP1 under nucleotide stress conditions [49]. Furthermore, several chromatin remodelers (e.g., SMARCA4, H2AZ, PRMT1, RCOR1) and architecture proteins (e.g., CTCF, MAX) displayed high enrichment scores at these enhancers (Fig. 3d), suggesting that disruption of epigenomic regulation on non-coding regulatory elements may be essential in melanoma tumorigenesis.

We applied a latent block model [50] to cluster the 66 significant enhancers according to their associated HRR type and degree of mutation, and we found that these enhancers could generally be partitioned into four major subsets (Fig. 3e). Class I enhancers are frequently lost in melanoma patients including some that are functional towards classical tumor suppressor genes (e.g., *PTEN*, *NF1*). Class II enhancers exhibit a heterogeneous mutation pattern wherein melanoma genomes can acquire different types of mutation in some functional loci (such as the non-coding region of *KLF6*). This highlights the context-specific regulatory re-wiring that occurs during tumorigenesis. Compared to Class I enhancers, Class III enhancers are sparsely mutated by different forms among melanoma patients, while Class IV enhancers are highly duplicated across many oncogene loci (such as *YAP1*). Previous genomic classification of melanoma patient based on the pattern of the most prevalent significantly mutated genes leads to four subtypes, including mutant *BRAF*, mutant *RAS*, mutant *NF1*, and Triple-WT (wild-type) [2]. However, we found that the mutation spectrum of these functional enhancers is generally independent of the mutational pattern, relying on presence of significantly mutated genes in melanoma.

To elucidate the epigenomic heterogeneity of enhancers across different melanoma contexts, we utilized publicly available H3K27ac and open chromatin signal data from tissue samples of melanoma patient [51–53]. We examined the occurrence of the 66 significant HRR-associated enhancers identified from our CRISPRi screening. Our findings suggested that over half of these enhancers are actively prevalent across various melanoma conditions (Additional file 10: Table S9, Additional file 11: S10). To investigate genetic heterogeneity, we examined the mutation status around the 66 significant enhancers, comparing these with melanocyte/melanoma cell lines in various carcinogenic stages from the Cancer Cell Line Encyclopedia (CCLE) [54]. In all 71 melanocyte/melanoma cell lines from the CCLE, including A375, we evaluated the presence of somatic copy number variations and structural variations (DEL, DUP, INV, TRA) within the enhancer genomic region. Our findings revealed that 21 of 66 enhancers, including E_156 and E_349, exhibited copy number gain events, and 17 of 66 showed heterozygous loss events (Additional file 12: Table S11). Together, these enhancer dysregulations may be influenced by the specific tumor evolution patterns at both genetic and epigenetic levels.

### Target gene analysis between melanoma-suppressive enhancers and known or novel cancer genes

**Fig. 4:**
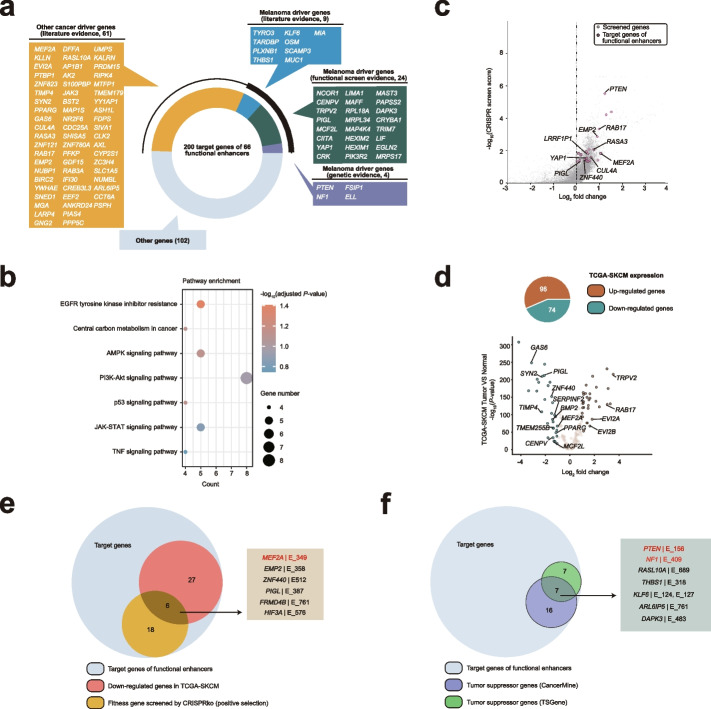
Target genes analysis of melanoma functional enhancers based on H3K27ac HiChIP. **a** Candidate target genes of the top 20 functional enhancers, genes are labeled with different colors according to different evidence sources. **b** KEGG pathway enrichment analysis of the target genes with cancer-related evidence. **c** Target gene distribution in public A375 CRISPR KO positive screen, significant target genes for the top 20 functional enhancers are labeled. **d** Target gene distribution in the differential expression analysis of TCGA-SKCM samples, significant target genes for the top 20 functional enhancers are labeled. **e** Overlaps among target genes of the functional enhancers, A375 CRISPR KO positive selected genes and significantly downregulated genes in TCGA-SKCM. **f** Overlaps among target genes of the functional enhancers and different layers of tumor suppressor evidence

In order to systematically probe the relationship between functional enhancers and cancer-related genes, we conducted an in-depth analysis of H3K27ac HiChIP data. This analysis led to the identification of 266 loops between 66 significantly screened enhancers via CRISPRi and 200 target genes, of which some have been previously recognized to play a critical role in constraining melanoma development (e.g., *PTEN*, *NF1*) (Additional file 13: Table S12). By systematically curating melanoma-associated cancer genes and other cancer genes from different resources, we annotated the 200 target genes linked to these essential enhancers (Fig. 4a, Additional file 14: Table S13). We noted that half of these target genes were previously confirmed to be cancer-associated genes, of which 18.5% are melanoma-associated genes with different types of evidence. This indicates that HRR-associated enhancers could modulate novel melanoma driver genes in carcinogenesis. Next, we performed the Kyoto Encyclopedia of Genes and Genomes (KEGG) enrichment analysis on these target genes, and we demonstrated that the functional HRR-associated enhancers revealed by our CRISPRi screen can drive tumor growth through several canonical cancer pathways, such as the EGFR signaling pathway, central carbon metabolism, and AMPK signaling pathway (Fig. 4b).

We illustrated the connectome for the top 20 essential enhancers and found that most enhancers contact multiple genes beyond the designed targets in the linear genome (Fig. 4c). The most significant CRISPRi screened enhancer E_349 interacts with *MEF2A* and *LRRC28* instead of its designed target *IGF1R*, which implies that HRR-associated enhancers could modulate multiple uncharacterized melanoma cancer genes for tumor survival. Furthermore, another significant enhancer E_223 targets the Hippo pathway melanoma oncogene *YAP1* and a multi-functional tumor suppressor *BIRC2*. This suggests that inhibition of certain HRR-associated enhancers could promote oncogenic expression by several unknown regulatory mechanisms in A375 cells. In addition, we found that some target genes, which interacted with the most active enhancers, were positively selected hits from the previous CRIPSR KO screens (Fig. 4d) or were differentially expressed between tumor and normal samples in the TCGA-SKCM cohort (Fig. 4e). Notably, several target genes were supported by complementary evidence to be tumor-suppressive in melanoma. For instance, *MEF2A*, *EMP2*, *ZNF440*, *PIGL*, *FRMD4B,* and *HIF3A* are not only downregulated in TCGA-SKCM melanoma samples but also essential for restraining tumor growth in CRIPSR KO screens (Fig. 4f). Similarly, *PTEN*, *NF1*, *KLF6*, and *THBS1* genes were frequently reported in different cancer gene resources as melanoma tumor suppressors (Fig. 4g). In summary, the integration of the enhancer connectome and CRISPRi screen identified several known or novel cancer genes in melanoma carcinogenesis.

### A super enhancer element modulates melanoma cell proliferation and apoptosis by targeting *MEF2A*

**Fig. 5:**
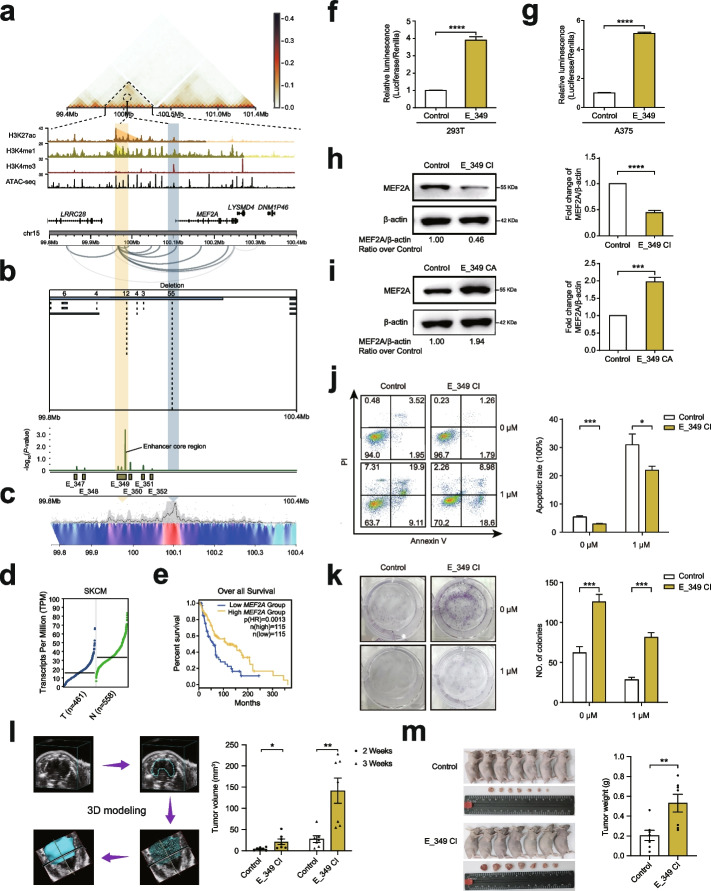
Loss of super enhancer element E_349 in melanoma unlocks tumor growth potential by modulating *MEF2A* expression. **a** Epigenetic and 3D genome profiles at the E_349-contained super enhancer and *MEF2A* locus (GRCh37/hg19 chr15: 99,800,000–100,400,000), including signals of A375 combined Hi-C, H3K27ac, H3K4me1, H3K4me3, ATAC-seq, and HiChIP loops. **b** Genomic profiles of recurrent DELs and CRISPRi enhancer screen results at the E_349 and *MEF2A* locus on 297 melanoma samples. **c** 4C assay result of interaction between the *MEF2A* promoter and E_349, light blue arrow refers to the 4C viewpoint (VP), and peak region highlighted by yellow arrow represents the chromosome region interacting with the VP. **d** Expression levels of *MEF2A* in tumor (T, *n* = 461 samples) and normal tissues (N, *n* = 558 samples) were analyzed using the TCGA-SKCM data. **e** Overall survival analysis of *MEF2A* in the low *MEF2A* expressed group and high *MEF2A* expressed group were compared using the TCGA-SKCM data. **f**, **g** The E_349 core sequence (GRCh37/hg19 chr15: 99,982,353–99,983,277) was cloned into pGL3-Promoter vector, and Luciferase assays were performed in 293T cells (**f**), and A375 cells (**g**). **h**, **i** Western blotting results of MEF2A protein expression in the E_349-inhibited A375-KRAB (**h**) and E_349-activated A375-VP64 (**i**) cells. **j**, **k** Cell apoptosis (**j**) and Plate clone formation (**k**) assay results of the E_349-inhibited A375-KRAB cells with 1 µM vemurafenib or without treatment (*n* = 3 samples). **l**, **m** The representative three-dimension (3D) modeling graphs and statistical results in calculating tumor volumes for E_349-inhibited A375-KRAB and control cells (**l**) at 2 or 3 weeks after subcutaneous injection in 5-week-old female nude mice, and then the tumors were weighed (**m**) after euthanasia of the mice (*n* = 7 mice). CI denotes CRISPR interference, and CA denotes CRISPR activation. Left graph is the representative result, and right graph is the statistical result. All the data are expressed as the means ± SD and analyzed by an unpaired two-tailed Student’s *t* test. Asterisks indicate significant differences between the indicated experimental groups: *, *p* < 0.05; **, *p* < 0.01; ***, *p* < 0.001; ****, *p* < 0.0001

We identified the enhancer E_349 (GRCh37/hg19 chr15: 99,982,353–99,983,277), located at a super enhancer locus and approximately 475 kb downstream of the designed gene *IGF1R*, as the most significant hit in our CRISPRi screens. *IGF1R* is overexpressed in melanoma and plays an oncogenic role by mediating tumor proliferation, motility, and protection from apoptosis [55], yet the relationship between E_349 and *IGF1R* gene was not supported by our A375 H3K27ac HiChIP. Instead, we found that E_349 can loop to the promoter region (125 kb downstream) of a developmental TF *MEF2A* gene (Fig. 5a), which has been documented to be important in cell differentiation, proliferation, and death [56, 57]. Given E_349 overlaps a DEL-HRR (30% recurrence rate) and its core region is highly mutated in melanoma patients (Fig. 5b), we sought to investigate whether this tumor-suppressive super enhancer element is essential in controlling melanoma growth by regulating *MEF2A*.

First, we performed the circularized chromosome conformation capture (4C) assay on the A375 cell line to evaluate the spatial proximity between E_349 and *MEF2A*. By selecting the promoter region (GRCh37/hg19 chr15: 100,125,127–100,127,180) of *MEF2A* as a viewpoint (VP), the 4C result showed that the VP region has a higher interaction frequency with the E_349 chromosomal region (Fig. 5c). This chromosome loop was further confirmed via the chromosome conformation capture (3C) assays on A375, SK-MEL-28 (another BRAF^V600E^ melanoma cell line), and SK-MEL-2 (no BRAF mutation melanoma cell line) cells at different conditions (Fig. 5c and Additional file 1: Fig. S5a-f). Then, we found that *MEF2A* is significantly downregulated in TCGA-SKCM tumor samples (tumor cell: 461 and normal cell: 558, adjusted *p*-value = 5.59e − 68, ANOVA test) (Fig. 5d). Furthermore, a lower expression of *MEF2A* was associated with a significantly decreased overall survival among TCGA patients (*p*-value = 0.0013, Kaplan–Meier estimate) (Fig. 5e). These data suggest a critical tumor-suppressive role for E_349 and its target gene *MEF2A* in melanoma growth and prognosis. In contrast, *LRRC28*, an alternative target of E_349 did not show clinical importance in the TCGA cohort (Additional file 1: Fig. S6a). We then evaluated the regulatory activity of E_349 in vitro by performing luciferase reporter assays, cloning the sequence of the E_349 core region into reporter vectors with promoter in 293T, A375, SK-MEL-28, and SK-MEL-2 cells, respectively. We observed that the E_349 core region significantly promotes luciferase activity in these four cell lines (all *p*-value < 0.0001) (Fig. 5f, g; Additional file 1: Fig. S7a, b). To further validate the regulatory function of E_349 in *MEF2A*, we packaged the lentivirus carrying the most significant enriched sgRNA for targeting of E_349, then transduced this into the A375-KRAB, A375-VP64, SK-MEL-28-KRAB, SK-MEL-28-VP64, SK-MEL-2-KRAB, and SK-MEL-2-VP64 cells, respectively. The results indicated that the expression of *MEF2A* is downregulated when E_349 is inhibited in A375 (Fig. 5h), SK-MEL-28 (Additional file 1: Fig. S7c), and SK-MEL-2 (Additional file 1: Fig. S7e) cells, and the expression of *MEF2A* is upregulated when E_349 is over-activated in A375 (Fig. 5i), SK-MEL-28 (Additional file 1: Fig. S7d), and SK-MEL-2 (Additional file 1: Fig. S7f) cells (all *p*-value < 0.001). Besides, we found that inhibition of E_349 cannot affect the expression of our designed gene *IGF1R* in A375, SK-MEL-28, and SK-MEL-2 cells (Additional file 1: Fig. S10a, d). Collectively, these data indicate that E_349 is a bona fide enhancer that directly regulates *MEF2A* expression.

Since the expression level of *MEF2A* is associated with patient prognosis, we speculated whether inhibition of E_349 could affect tumor survival phenotypes and drug sensitivity (e.g., vemurafenib). Notably, our 3C data indicated that vemurafenib cannot affect the interaction between E_349 and the *MEF2A* promoter (Additional file 1: Fig. S5d, e). We performed the cell proliferation, apoptosis, and flat colony formation assays on A375-KRAB, SK-MEL-28-KRAB, or SK-MEL-2-KRAB cells under treatment, with or without vemurafenib. We found that the proliferative abilities of the cells in the E_349-inhibited group were markedly higher than those in the control group (all *p*-value < 0.001) (Additional file 1: Fig. S7h, i, k). Furthermore, the cell apoptosis abilities of the cells in the E_349-inhibited group were significantly lower than those in the control group (all *p*-value < 0.05) (Fig. 5j and Additional file 1: Fig. S7l). Finally, the colony formation abilities of the cells in the E_349-inhibited group were significantly higher than those in the control group (all *p*-value < 0.01) (Fig. 5k, Additional file 1: Fig. S7m, n). To further access the E_349’s function in melanoma growth, we used CRISPR technology to knock out the E_349 core region in A375 cells (Additional file 1: Fig. S11a, b). Expectedly, we acquired the similar phenotypes with the E_349 inhibition (all *p*-value < 0.0001) (Additional file 1: Fig. S7g, j); when the E_349 core region was deleted, the interaction between *MEF2A* promoter and the region harboring E_349 was lost (Additional file 1: Fig. S5g), further validating the E_349 directly regulates *MEF2A* expression in A375 cells. Next, we used the cell-derived xenograft (CDX) model to further investigate the function of E_349 in melanoma growth in the nude mice. As a result, the subcutaneous A375-derived cells in the E_349-inhibited group were significantly larger (all *p*-value < 0.05) (Fig. 5l) and heavier (*p*-value < 0.01) (Fig. 5m) than those in the control group, highlighting the important function of E_349 in A375 tumor growth. Finally, to access the E_349’s function in melanoma patients, we collected patient melanoma tissues, extracted the genomes, and amplified the DNA sequence harboring E349 core region for Sanger sequencing. Fortunately, of 122 melanomas, we identified two samples with deletions or extensive mutations in E349 (Additional file 1: Fig. S12a, b). The immunohistochemistry (IHC) results indicated that E_349, when deleted or extensively mutated, showed a relatively low expression of MEF2A and a markedly stronger ability for tumor proliferation (Additional file 1: Fig. S12c). This highlights the importance of E349 in melanoma growth. Taken together, these results demonstrate loss of E_349 in melanoma can unlock tumor growth potential and promote vemurafenib resistance.

### A distal enhancer sustains *PTEN* tumor-suppressive potential in melanoma cells

**Fig. 6:**
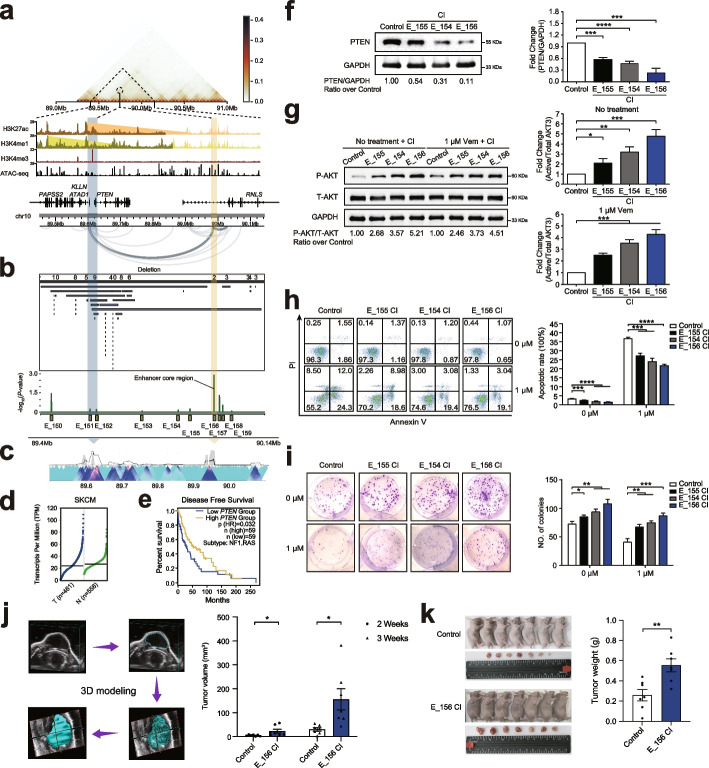
Long-range interaction between E_156 and *PTEN* maintains melanoma-suppressive function. **a** Epigenetic and 3D genome profiles at the *PTEN* and its downstream enhancer locus (GRCh37/hg19 chr10: 89,400,000–90,140,000), including signals of A375 combined Hi-C, H3K27ac, H3K4me1, H3K4me3, ATAC-seq, and HiChIP loops. **b** Genomic profiles of recurrent DELs and CRISPRi enhancer screen results at the E_156 and *PTEN* locus on 297 melanoma samples. **c** 4C assay result of interaction between the *PTEN* promoter and E_156, light blue arrow refers to the 4C viewpoint (VP), and peak region highlighted by yellow arrow represents the chromosome region interacting with the VP. **d** Expression levels of *PTEN* in tumor (T, *n* = 461 samples) and normal tissues (N, *n* = 558 samples) were analyzed using the TCGA-SKCM data. **e** Disease-free survival analysis of *PTEN* expression were analyzed using the TCGA-SKCM data. Low expression of *PTEN* predicts shorter disease-free survival. **f** Western blotting results of *MEF2A* protein expression in the E_156 (E_155 or E_154 adjacent to E_156)-inhibited A375-KRAB cells. **g** Western blotting results of PI3K/AKT signaling pathway activation in the E_156 (E_155 or E_154 adjacent to E_156)-inhibited A375-KRAB cells with 1 µM vemurafenib or without treatment. P-AKT denotes Phosphorylated AKT, and T-AKT denotes total AKT. **h**, **i** Cell apoptosis (**h**) and Plate clone formation (**i**) assay results of the E_156 (E_155 or E_154 adjacent to E_156)-inhibited A375-KRAB cells with 1 µM vemurafenib or without treatment (*n* = 3 samples). **j**,**k** The representative three-dimension (3D) modeling graphs and statistical results in calculating tumor volumes for E_156-inhibited A375-KRAB and control cells at 2 or 3 weeks after subcutaneous injection in 5-week-old female nude mice (**j**), and then the tumors were weighed (**k**) after euthanasia of the mice (*n* = 7 mice). CI denotes CRISPR interference, and CA denotes CRISPR activation. Left graph is the representative result, and right graph is the statistical result. All the data are expressed as the means ± SD and analyzed by an unpaired two-tailed Student’s *t* test. Asterisks indicate significant differences between the indicated experimental groups: *, *p* < 0.05; **, *p* < 0.01; ***, *p* < 0.001; ****, *p* < 0.0001

Recurrent deletion of *PTEN* gene is a critical driver for melanoma tumorigenesis and metastasis [58, 59]. However, not all of the melanoma cases with loss of PTEN expression can be explained by the disruptions in its coding or splicing regions, emphasizing the importance for the identification of non-coding regulatory mechanisms mediating PTEN loss. In our endogenous screen, we revealed a significant enhancer (E_156) located near *PTEN* whose inactivation could facilitate melanoma cell growth. E_156 (GRCh37/hg19 chr10: 89,986,509–89,987,396) was the second-ranked hit in the screen, which was located 375 kb downstream of the *PTEN* gene and shows frequent interaction with the *PTEN* promoter (Fig. 6a). In melanoma patients, in addition to highly recurrent mutations in the protein-coding region, *PTEN* acquires many independent upstream/downstream non-coding mutations, and many of them overlap with HRR-associated enhancers (Fig. 6b). To verify whether E_156 directly regulates the expression of *PTEN*, we performed 4C and 3C assays on melanoma cell lines by selecting the promoter region (GRCh37/hg19 chr10: 89,620,814–89,627,996) of *PTEN* as a VP. Both 4C and 3C results showed that the *PTEN* promoter had a higher interaction frequency with the enhancer core region of E_156 at different conditions (Fig. 6c, Additional file 1: Fig. S8a-f), supporting the regulatory relationship of the distal enhancer E_156 on *PTEN* in melanoma.

Given the clinical significance of *PTEN*, but not the alternative target gene *ATAD1* and *KLLN* (Fig. 6d, e; Additional file 1: Fig. S6b), as well as the evidence of direct interaction between E_156 and the *PTEN* promoter in melanoma, we evaluated the tumor-suppressive function of E_156 in vitro. For comparison, we also measured the effect of two closer enhancers, E_155 and E_154, which show moderate signals in our screen. We first performed luciferase reporter assays by cloning the sequence of their core regions into reporter vectors with promoters in 293T, A375, SK-MEL-28, and SK-MEL-2 cells, respectively. We observed that all these enhancer core regions significantly promote luciferase activity in all four cell lines (all *p*-value < 0.0001) (Additional file 1: Fig. S9a-c, n), in which E_156 displays relatively higher activity. By constructing enhancer-perturbed A375-KRAB, A375-VP64, SK-MEL-28-KRAB, SK-MEL-28-VP64, SK-MEL-2-KRAB, and SK-MEL-2-VP64 cell lines, we found that the expression of *PTEN* was downregulated or upregulated when the enhancers were inhibited (all *p*-value < 0.05) (Fig. 6f, Additional file 1: Fig. S9d, g, o) or activated (all *p*-value < 0.05) (Additional file 1: Fig. S9e,f,h, p), further validating our CRISPR screen result. Besides, we found that inhibition of E_156 cannot affect the expression of the alternative target gene *ATAD1* and *KLLN* in A375, SK-MEL-28, and SK-MEL-2 cells (Additional file 1: Fig. S10b, c, e, f). Since the PTEN/PI3K/AKT signaling pathway plays a key role in cell proliferation, growth, and apoptosis [60], we speculated whether these enhancers could affect tumor viability or drug response via the PI3K/AKT signaling pathway in melanoma cells. As expected, the phosphorylated AKT was upregulated in the enhancer-inhibited A375-KRAB and SK-MEL-28-KRAB cells (all *p*-value < 0.05) (Fig. 6g, Additional file 1: Fig. S9i), in which E_156 exhibited a stronger effect on AKT activation. Furthermore, cell proliferation, apoptosis, and flat colony formation assays were applied to A375-KRAB, SK-MEL-28-KRAB, and SK-MEL-2-KRAB cells treated with or without vemurafenib. Consequently, the proliferative abilities of the cells in the enhancer-inhibited group were all markedly higher than those in the A375-KRAB, SK-MEL-28-KRAB, and SK-MEL-2-KRAB control group (all *p*-value < 0.01) (Additional file 1: Fig. S9j, k, t). For cell apoptosis, abilities of the cells in the enhancer-inhibited group were all significantly lower than those in the A375-KRAB, SK-MEL-28-KRAB, and SK-MEL-2-KRAB control group (all *p*-value < 0.01) (Fig. 6h, Additional file 1: Fig. S9l). Finally, for the colony formation abilities of the cells in the enhancer-inhibited group, these were all markedly higher than those in the A375-KRAB, SK-MEL-28-KRAB, and SK-MEL-2-KRAB control group (all *p*-value < 0.05) (Fig. 6i, Additional file 1: Fig. S9m, r). To further access the E_156’s function in melanoma growth, we used CRISPR technology to knock out the E_156 core region in A375 cells (Additional file 1: Fig. S11c, d). As expected, we acquired the similar phenotypes with the E_156 inhibition (all *p*-value < 0.01) (Additional file 1: Fig. S9q, s); when E_156 core region was deleted, the interaction between *PTEN* promoter and the region harboring E_156 was lost (Additional file 1: Fig. S8g), further validating the E_156 directly regulates *PTEN* expression in melanoma growth. Next, we used the CDX model to further investigate the E_156’s function in melanoma growth in the nude mice. As a result, the subcutaneous A375 cells in the E_156-inhibited group was markedly larger (all *p*-value < 0.05) (Fig. 6j) and heavier (all *p*-value < 0.05) (Fig. 6k) than those in the control group, highlighting the important function of E_156 in A375 tumor growth. However, due to scarcity of collected samples, we did not find the E156 deletions or extensive mutations among the 122 melanoma patient’s samples. Taken together, among the three enhancers, inhibition of E_156 consistently resulted in a greater effect on tumor survival phenotypes, which highlights the importance of the distal enhancer in sustaining *PTEN* tumor-suppressive potential in melanoma cells.

## Discussion

The high mutation burden in the melanoma genome provides a major challenge in identifying true driver events from the background mutations, especially in the non-coding genomic regions. The dilemma could be attributed to multiple factors such as limited sequenced melanoma samples, overestimated mutation hotspots, paucity of non-coding drivers, and relatively low-throughput functional validations. In the present study, we systematically integrated 297 melanoma WGS datasets and developed a strategy to estimate HRRs for both SV and SNV/INDEL. Consequently, we present one of the largest genome-wide studies on melanoma genomics. We designed a genome-scale CRISPRi screening method to investigate the functional consequence of HRR-associated enhancers and uncovered 66 significant enhancers, which could play growth-suppressive roles in melanoma cells. These functional enhancers colocalize with several critical melanoma-associated TFs and are generally independent of the mutational pattern of classically significantly mutated genes in melanoma. By applying H3K27ac HiChIP chromatin loop detection, we identified 200 target genes linked to the functional HRR-associated enhancers, and some of these may be implicated as being potentially novel mechanisms in melanoma. In-depth functional assays demonstrated that the two highest ranked enhancers are critical in sustaining *MEF2A* and *PTEN* tumor-suppressive potential in melanoma cells.

An increasing number of studies have confirmed that disruption of CREs, such as enhancer, represents an essential tumor-driven mechanism in multiple cancers [40, 42, 61–63]. This demonstrates that CREs could be an attractive area for therapeutic targets in cancers. Despite the constant endeavor to identify driver events in highly mutated melanoma genomes [2, 3, 5–7], study for systematic assessing non-coding HRRs and their associated functional elements on substantial melanoma samples is still lacking. In order to attain the requisite sample size, this study combined high-coverage melanoma WGS data from different cohorts (including European, Australian, Brazilian, and other populations) and various cancer subtypes (including cutaneous melanoma and few mucosal/acral melanoma), which may confound the identification of true HRR and compromise the detection specificity. On the other hand, in this study, despite the significant insights into functional HRR-associated enhancers in melanoma that our study provides, we recognize its limitations. In our analysis, the selection of screened enhancers and chromatin interaction measurement based on A375 cell line did not fully account for the complexities inherent in epigenomic heterogeneity and 3D genome looping structures across different melanoma contexts. Epigenomic heterogeneity, a fundamental aspect of cancer cells, and 3D genome organization vastly influence gene expression and cellular functions [64], with evidence of substantial variances across different cell types. Thus, to advance our understanding of the intricate gene regulation in melanoma, future investigations should systematically consider these factors.

Previous CRISPR screen of CREs on melanoma cells has been performed on several critical genes, including *NF1*, *NF2*, and *CUL3*, whose loss-of-function mutations resulted in vemurafenib resistance [23]. By designing sgRNA libraries tiling across 100-kb regions surrounding the three genes, the CRISPR KO screen yielded many functional CREs in the non-coding region. Intriguingly, among 66 significant HRR-associated enhancers in our CRISPRi screen, two are located proximal to *NF1* and another two are in the vicinity of *NF2*. This indicates that loss of *NF1* or *NF2* expression will benefit tumor growth and thus primarily confer vemurafenib resistance. In addition, compared with existing genome-wide CRISPR screening of CREs in other cancer types [21, 22, 65, 66], our CRISPRi screen focused on potential enhancers for all candidate essential genes associated with melanoma cell growth and drug resistance. Nevertheless, we accept that this screening strategy will inevitably miss other types of CREs (e.g., insulators, silencers, or unmarked regulatory elements [67]) and most of the undiscovered melanoma driver genes, which are out of the regulatory scope of the desired enhancers. In addition, background amplification could impact positive selection screens at day21. In this study, we attempted to control for background amplification by eliminating low-count guides, using non-targeting control guides for normalization, and estimating significant enhancer-level enrichment with tiling sgRNAs. However, our screen strategy without selecting for a specific phenotype, based on continuous melanoma cell proliferation, might introduce unwanted background amplification. Furthermore, comparisons between day7 and day21 screens revealed limited consistent hits, likely due to the diminishing power of drop-out screening over time. This highlights the need to carefully select screening timeframes and enrichment strategies in future studies, taking into account the specific readouts.

Given that we found 66 significant HRR-associated enhancers in our CRISPRi screen, many connect to distal targets or regulate uncharacterized melanoma driver genes according to the H3K27ac HiChIP. The highest significant screened enhancer E_349 loops to the promoter of *MEF2A* instead of its designed target *IGF1R*. The context-dependent function of *MEF2A* confers its tumor-suppressive or oncogenic activity in different cancers [68]. However, *MEF2A* is poorly characterized and has been rarely studied in melanoma. By combining genomic, functional, and clinical evidence, we discovered that the critical enhancer, E_349, within a super enhancer region, plays a tumor-suppressive role in melanoma through modulation of *MEF2A* expression. In contrast, a *PTEN* enhancer was previously identified to be essential in maintaining tumor-suppressive function in T-cell leukemia [24]. Consistent with this finding, we found several distal downstream enhancers could sustain *PTEN* tumor-suppressive potential in melanoma. In summary, our study provides a comprehensive catalogue of crucial enhancers and their target genes in melanoma growth and progression. These results will not only facilitate the discovery of novel melanoma drivers, but also promote the fine-grained molecular classification and therapeutic optimization of melanoma. Interestingly, we also discovered that some significant HRR-associated enhancers are spatially proximal to several well-known melanoma oncogenes, such as *YAP1*. This proximity is likely due to the specific effects of CRISPRi at certain loci or a non-traditional gene regulatory mechanism. These findings merit further in-depth research [25].

## Conclusions

Our study establishes a catalogue of crucial enhancers and their target genes in melanoma growth and progression, and illuminates the identification of novel mechanisms of dysregulation for melanoma driver genes and new therapeutic targeting strategies.

## Methods

### Cell lines

A375 (American Type Culture Collection [ATCC], CRL-1619) and A375-derived human melanoma cell lines, 293T cell line (ATCC, CRL-3216), and 293FT cell line (ThermoFisher, R70007) were all cultured in Dulbecco’s modified Eagle’s medium (DMEM; ThermoFisher, 11965092) supplemented with 10% fetal bovine serum (FBS; ThermoFisher, R7007). SK-MEL-2 (ATCC, HTB-68), SK-MEL-2-derived, SK-MEL-28 (ATCC, HTB-72), and SK-MEL-28-derived human melanoma cell lines were maintained in Eagle’s Minimum Essential Medium (EMEM; ATCC, 30–2003) supplemented with 10% FBS. All cell lines were cultured at 37 °C in a humidified atmosphere containing 5% CO_2_ and were verified mycoplasma-free using the MycoAlert Mycoplasma Detection Kit (Lonza, LT07-218).

### WGS data and somatic mutation calling

WGS raw data of 297 paired melanoma tumor and normal samples were collected (MELA-AU: 183 samples, SKCM-US: 38 samples, and SKCA-BR: 76 samples) from the International Cancer Genome Consortium (ICGC) and European Genome-phenome Archive (EGAD00001003388) databases. For SNVs/INDELs, we directly extracted the GATK Mutect2 call sets [30] of MELA-AU and SKCM-US from the ICGC. To ensure consistent mutation calling, GATK (v4.1.4.0) and Mutect2 [30] were used to pre-process and call somatic SNV/INDELs on each tumor-normal paired sample of SKCA-BR using the parameters recommended in the GATK Best Practices. The generated mutation calls were further filtered using GATK FilterMutectCalls module and the final somatic output in VCF format was annotated and converted by VEP (v1.6.19) [69]. Considering the diversity and complexity of SV detection tools, we applied both Manta (v1.6.0) [31] and GRIDSS (v2.9.2) [32] to detect somatic SVs with default parameters for tumor-normal paired samples. The two SV calling results were used for subsequent analysis after removing all insertions and other SVs with fragment lengths over 1 Mb. The human assembly GRCh37/hg19 was used across the whole analysis.

### HRR detection and prioritization

We used fishHook [33] to detect genome-wide HRRs on somatic SNV/INDELs. Briefly, we first clustered mutations within 200 bp and collected three cell type-specific epigenomic features of epidermal keratinocyte primary cell (NHEK) as covariates, including chromatin accessibility, ChromHMM chromatin state, and DNA replication timing. Then, fishHook Gamma-Poisson model was used to correct the three epigenomic features, nucleotide context, and mutation cluster length for SNV/INDEL-HRR estimation. For detecting and prioritizing SV-HRRs, we leveraged a sliding window strategy to calculate the recurrence of somatic SVs among melanoma patients within each window (10 kb window size and 2 kb step size for DELs and DUPs, 1 kb window size, and 200 bp step size for INVs and TRAs). Subsequently, we located the window with the largest sample counts (at least four patients) and merged the adjacent windows on both sides. The merged interval whose total recurrent count ≥ 10 was defined as candidate SV-HRR. Since we produced two sets of SV-HRR by different SV callers (Manta and GRIDSS), we used BEDTools [70] merge function to obtain unique HRRs, wherein the SV-HRRs were labeled as “Dual” if they are detected by both tools. To facilitate the prioritization of SV-HRRs at patient level, we also merged all types of overlapped SV-HRRs and recalculated the recurrence count.

### HRR and HRR-associated enhancer annotation

We annotated the genomic attributes of each HRR based on RefSeq database [71], and we defined melanoma driver/essential genes from different angles, including genetic evidence, functional evidence, and literature evidence. Candidate melanoma driver genes with genetic evidence carrying significant mutations were integrated from several large-scale sequencing studies and reviews [2, 3, 10]. Melanoma essential genes with functional evidence were collected from The Cancer Dependency Map (DepMap) [72, 73] skin-related results (*p*-value < 0.05). Putative melanoma driver/essential genes or other non-melanoma cancer genes were compiled from public cancer gene databases including CancerMine [26], IntOGen [27], and NCG [28]. In addition, we uniformly processed H3K27ac ChIP-seq from 10 melanoma cell lines, including LOX_IMVI, M14, MALME-3 M, MDA-MB-435, SK-MEL-2, SK-MEL-28, SK-MEL-5, UACC-257, and UACC-62 were from GSE143653 [74], and A375 cell line was from GSE99835 [75] and GSE82332 [76]*.* All ChIP-seq raw data were mapped to the human genome (GRCh37/hg19) using Bowtie2 [77] and narrow peaks were called by MACS2 [78] with default parameters. We intersected all significant SNV/INDEL-HRRs (FDR < 5%) and SV-HRRs (≥ 10 donors) with these H3K27ac peaks to define HRR-associated enhancers.

### Functional evaluation of HRR-associated enhancer

We re-analyzed three public A375 CRISPR KO screening datasets, including two for positive screen [44, 79] and one for negative screen [45], to retrieve melanoma essential genes using MAGeCK-RRA algorithm [46], yielding two significant gene sets from positive and negative selection, respectively. By assigning presumed target gene (1 Mb near enhancer) to each HRR-associated enhancer or HRR-unrelated enhancer, we tested the difference of CRISPR enrichment scores between gene groups via Mann–Whitney *U* test across five HRR types.

### CRISPRi sgRNA library construction

We first comprehensively curated essential genes associated with melanoma cell growth and drug resistance from published CRISPR/shRNA screening studies. Then, we selected HRR-associated enhancers receiving both H3K27ac and H3K4me1 signals in A375 cells within 1 Mb region of each melanoma essential genes. These enhancer regions were further narrowed with chromatin accessibility signal based on ATAC-seq peaks. For the remaining enhancer core regions, we divided each region into several bins consisting of 100 bp and scored all eligible sgRNAs in each bin using FlashFry [80]. By selecting the best sgRNA in each bin, the intersected tiling strategy can yield a sgRNA library for all selected enhancers, and a total of 1000 unmatched sgRNAs were added into the library as negative control. The sgRNA sequences were synthesized by Synbio Technologies and cloned into lentiGuide-Puro vector (Addgene, 52963) in the form of homologous recombination to construct the plasmid library, and then packaged into the lentivirus library using the pMD2.G (Addgene, 12259) and psPAX2 (Addgene, 12260) packaging plasmids as mentioned below for subsequent screening.

### CRISPRi screen

We generated A375 or SK-MEL-2 cell lines stably expressing Lenti-dCas9-KRAB (Addgene, 89567) transgenes and performed lentiviral transduction of pooled sgRNAs at low multiplicity of infection (MOI) = 0.2. After 24 h, puromycin was added for selecting the effective infected A375-KRAB or SK-MEL-2 cells. After 7 days, the cells were divided into two groups. One group (day 0) of cells was collected and frozen as a control group, and the other groups (day 7 or 21) of cells were selected at 7 or 21 days. Genomic DNAs of the two group cells were extracted by FastPure Cell/Tissue DNA Isolation Mini Kit (Vazyme, DC102) for PCR amplification (Vazyme, N616), and the amplified products were subject to library construction using the VAHTS Universal DNA Library Prep Kit for Illumina V3 (Vazyme, ND607) according to the kit’s instructions and then performed the next-generation sequence on a HiSeq X Ten platform. The CRISPRi screen was independently performed for two times.

### CRISPRi positive selection enrichment

Two replicates of day 0 group and day 21 group were sequenced and undergone quality control using MAGeCK-VISPR [81]. We first extracted the sgRNA sequence from the 150 bp original reads, and the abundance of guides was first determined by the MAGeCK “count” module from the extracted sgRNAs. Second, we removed 1% guides of each sample with lowest count. We used the non-targeting control guides to estimate the size factor for normalization, and estimated significant enrichment according to MAGeCK-RRA algorithm [46] at enhancer level. Finally, significant enhancers were identified with FDR < 0.05.

### Mutational signature analysis

To investigate the mechanism involved in functional HRR-associated enhancer, we extracted single-base-pair substitutions (SBS) in enhancer regions and used Palimpsest [47] to calculate de novo mutational signatures. We then calculated cosine similarity values between de novo signature and known COSMIC mutational signatures. COSMIC signature with highest correlation coefficient was regarded as candidate predominant signatures among enhancers. We also performed component analysis of known melanoma signatures for each functional enhancer.

### Genomic and epigenomic features

We downloaded epigenetic signatures of A375 cell line from public repositories, including CTCF (GSM3671692) [82], H3K4me3 (GSM2653904) [83], and Pol II ChIP-seq (GSM1661790) [49], together with PRO-seq (GSE128081) [84] and super enhancer [76]. The TFs narrowPeak were downloaded from Gene Expression Omnibus (GEO), including MITF (GSE149929) [85], HEXIM1 (GSM1661788) [49], TFAP2C (GSM1011562) [86], ETV1 (GSM2127445) [87], BRD4 (GSM2359435) [88], AR (GSM3212792) [89], EGR1 (GSM3212795) [89], DDX21 (GSE149929) [85], CDK9 (GSM3664674) [84], USP7 (GSM3928164) [90], EZH2 (GSM3928166) [90], and AP2A (GSM4950452) [91]. All ChIP-seq datasets were uniformly processed by MACS2 [78]. PRO-seq was analyzed using PEPPRO [92] and dREG [93] for predicting nascent enhancer RNA with bidirectional transcription. Transcription factor colocalization analysis was performed by epiCOLOC [94]. To investigate the mutation pattern of significant enhancers, we first built a categorical matrix based on the HRR type on enhancer region for MELA-AU cutaneous melanoma patients (*N* = 137). Then we applied a latent block model [50] to cluster the categorical matrix and compared with previous molecular classification (mutant *BRAF*, mutant *RAS*, mutant *NF1*, and Triple-WT) defined by TCGA.

### In situ Hi-C followed by chromatin immunoprecipitation (HiChIP)

H3K27ac HiChIP was conducted following the previously published procedures [95]. Briefly, A375 cells were counted, and ~ 1.5 × 10^7^ cells were crosslinked with 1% formaldehyde (Sigma-Aldrich, F8775) in phosphate-buffered saline (PBS; ThermoFisher, 10010023) at room temperature for 10 min, and then quenched with 0.125 M Glycine (Sigma-Aldrich, 50046) on ice for 5 min. Next, crosslinked cells were lysed in Hi-C lysis buffer (10 mM Tris–HCl pH 8.0 [ThermoFisher, 15568025], 10 mM NaCl [Sigma-Aldrich, S7653], and 0.2% Igepal CA630 [Sigma-Aldrich, I3021]) with 50 μl of protease inhibitors (Sigma, P8340), and nuclei were extracted and digested with *Mbo*I restriction enzyme (NEB, R0147) for 2 h. After digestion, nuclei were resuspended in NEB buffer supplemented with DNA polymerase I, Large (Klenow) fragment (NEB, M0210) to fill in the restriction fragment overhangs with Biotin-14-dATP (Life Technologies, 19524–016) for 1 h. Proximal ligation was performed with T4 DNA ligase (NEB, M0202) for 4 h at 16 °C and nuclei were harvested. The nuclei were transferred to shear chromatin using a Biorupter system (Diagenode, B01060001). For each sample, the sheared chromatin was incubated overnight with 8 μl of H3K27ac antibody (Abcam, ab4729), and then Protein A-agarose beads (Millipore, 16–157) were used to capture H3K27Ac-associated chromatin. The whole H3K27ac-ChIP process was performed with the Pierce™ Magnetic ChIP kit (ThermoFisher, 26157), the final DNA was eluted with 15 μl Nuclease-Free Water (ThermoFisher, AM9930). The eluted DNA was sent for DNA biotin pull-down with Dynabeads MyOne Streptavidin T1 beads (Life technologies, 65602). Sixty micrograms of DNA was used for PCR amplification, and then AMPure XP beads (Beckman Coulter, A63880) was used to select a size range of 300 ~ 600 bp products. Finally, the HiChIP libraries were constructed using the VAHTS Universal DNA Library Prep Kit for Illumina V3 according to the kit’s instructions and then performed the next-generation sequence on a HiSeq X Ten platform.

### HiChIP loop detection and target gene annotation

HiChIP paired-end reads were aligned and analyzed using the FitHiChIP pipeline [96] with default settings to remove duplicate reads, assign reads to *Mbo*I restriction fragments, filter for valid interactions, and generate binned interaction matrices. Chromatin interactions were filtered from a minimum distance of 20 kb to a maximum of 2 Mb. To identify target gene of significant functional enhancers, we defined gene promoters as 20 kb upstream and 5 kb downstream to the transcription start sites. We intersected these promoters with significant contacts and left only those contacts for which one of the anchors fell inside the promoter region, and another anchor, therefore, was assigned to functional enhancer region we defined above. We performed KEGG enrichment analysis on target genes with cancer-related evidence using ClusterProfiler [97]. Supporting evidence of target genes on melanoma growth were further mined by differential expression analysis of TCGA-SKCM samples using GEPIA2 [98], as well as public A375 CRISPR KO positive screen [44, 79]. TSGene [99] and CancerMine [26] were also used to annotate known tumor suppressive genes. Our A375 H3K27ac HiChIP identified a total of 121,775 loops, of which 68,998 loops contain valid Enhancer-Promoter pairs. Enhancers are co-defined by A375 H3K27ac, H3K4me1, and ATAC-seq signals, and promoters of the genes are defined as 3 kb near the transcriptional start site (TSS).

### Circularized chromosome conformation capture (4C) assay

4C assays were performed as previously described [100] with slight modifications. For *MEF2A*, the *Nla*III-cutting chromosome region harboring the *MEF2A* promoter (GRCh37/hg19 chr15: 100,125,127–100,127,180) was designed as a viewpoint (VP), and *Dpn*II was the second enzyme. For *PTEN*, the *Hin*dIII-cutting chromosome region harboring the *PTEN* promoter (GRCh37/hg19 chr10: 89,620,814–89,627,996) was designed as a VP, and *Nla*III was the second enzyme. Briefly, ~ 1 × 10^7^ A375 cells were crosslinked by 2% formaldehyde, quenched with 0.125 glycine (Sigma-Aldrich, 50046), lysed with cold lysis buffer (50 mM Tris pH 7.5 [ThermoFisher, 15567027], 0.5% NP-40 [ThermoFisher, FNN0021], 150 mM NaCl, 1% Triton X-100 [ThermoFisher, T8787], 5 mM EDTA [ThermoFisher, 15575020], and 1 × protease inhibitors [ThermoFisher, 78425]), digested with *Nla*III (NEB, R0125L) or *Hin*dIII (NEB, R0104) for 4 h at 37 °C while shaking at 800 revolutions per minute (RPM) for more than three times until the digestion efficiency reached over 90%, and then ligated with T4 DNA ligase for overnight in a 7-ml reaction system after enzyme inactivation. The ligation sample was purified using the Phenol/Chloroform/Isoamyl Alcohol (25:24:1) (ThermoFisher, 15593–049) before reversing the cross-links by adding Protein K (ThermoFisher, AM2548) for 6 h at 65 °C and RNase A (ThermoFisher, 15596–018) for 1 h at 37 °C. Next, the DNA samples were digested with *Dpn*II (NEB, R0543L) or *Nla*III and ligated with T4 DNA ligase for more than 6 h in a 14-ml reaction system. The ligation product was purified using the Phenol/Chloroform/Isoamyl Alcohol (25:24:1) and further purified using the QIAquick PCR Purification Kit (QIAGEN, 28106). The purified product was amplified by PCR using a high-fidelity DNA polymerase (Vazyme, N616-02) with 8 × 200 ng DNA. 4C-seq library was constructed using the VAHTS Universal DNA Library Prep Kit for Illumina V3 according to the manufacturer’s description and sent for next-generation sequencing. pipe4C [101] were used to filter, analyze, and visualize the 4C-seq data. Primers used are listed in Additional file 15: Table S14.

### Chromosome conformation capture (3C) assay

3C assays were conducted as previously described [102, 103]. For *MEF2A*, we chose the promoter-containing region (GRCh37/hg19 chr15: 100,123,291–100,129,474) as a VP. For *PTEN*, the bait in 3C assays is the same as the VP used in the promoter 4C assay. Briefly, a total of 1 × 10^7^ A375-KRAB, SK-MEL-28-KRAB, or SK-MEL-2-KRAB cells treated with 1 μM vemurafenib (Selleck, RG7204) for 2 days or no treatment were used for 3C assays. The cells were crosslinked utilizing 2% formaldehyde, quenched with 0.125 M glycine, lysed with cold lysis buffer (50 mM Tris pH 7.5, 0.5% NP-40, 150 mM NaCl, 1% Triton X-100, 5 mM EDTA, and 1 × protease inhibitors), digested with *Hin*dIII for 4 h at 37 °C while shaking at 800 RPM for more than three times until the digestion frequency reached over 90%, and then ligated with T4 DNA ligase for overnight in a 7-ml reaction system after enzyme inactivation. The ligation samples were purified using the Phenol/Chloroform/Isoamyl Alcohol (25:24:1) before reversing the cross-links by adding Protein K for 6 h at 65 °C and RNase A for 1 h at 37 °C. The potential crosslinked-fragments were amplified using Premix Taq enzyme (TaKaRa, RR901A). The sequences harboring the detected *Hin*dIII loci were separately amplified by the Phanta Max Super-Fidelity DNA Polymerase (Vazyme, P505-d3). Concentrations of the sequences were determined by the Qubit dsDNA HS Assay Kit (ThermoFisher, Q32851). The same copy number of each sequence was blended as a reference control and subjected to proportional treatment as the experimental group. PCR products were electrophoresed on a 1.5% agarose gel and analyzed by ImageJ2 (https://imagej.net/ImageJ). Interaction frequency of each chromatin fragment was normalized by its reference control. Primers used are listed in Additional file 15: Table S14.

### Luciferase reporter assay

Genomic sequences harboring enhancer E_349 (GRCh37/hg19 chr15: 99,982,353–99,983,277), E_155 (GRCh37/hg19 chr10: 89,912,175–89,913,031), E_154 (GRCh37/hg19 chr10: 89,875,711–89,876,693), or E_156 (GRCh37/hg19 chr10: 89,986,509–89,987,396) core region were amplified from the genomic DNA of A375 cells, SK-MEL-28, or SK-MEL-2 cells and separately cloned into the downstream of luciferase gene in the pGL3-Promoter vector (Promega, E1761). After sequencing at the Beijing Genomics Institute (BGI), concentrations of the recombinant plasmids were exactly determined by the Qubit dsDNA HS Assay Kit (ThermoFisher, Q32851). The same copy numbers (equal to 1 µg of the pGL3-Promoter vector) of each recombinant plasmid together with 40 ng of pRL-TK renilla luciferase control vector (Promega, E2241) was transfected into 293T, A375, SK-MEL-28, or SK-MEL-2 cells in 24-well plates using the Lipofectamine 2000 transfection reagent (ThermoFisher, 1168019) following the manufacturer’s description. After 12 h, the transfected cells were lysed and assayed for fluorescence levels before assaying luciferase activity using the Dual-Luciferase Reporter Assay System (Promega, E1960) according to the manufacturer’s protocol. Relative luminescent signals were determined by normalizing firefly luciferase signals with renilla luciferase signals. Primers used are listed in Additional file 15: Table S14.

### Enhancer perturbation and knockout assays

For enhancer perturbation assay, A375-KRAB, SK-MEL-28-KRAB, SK-MEL-2-KRAB, A375-VP64, SK-MEL-28-VP64, and SK-MEL-2-VP64 cells were generated using the Lenti-dCas9-KRAB (Addgene, 89,567) and Lenti-dCas9-VP64 (Addgene, 61,425) plasmids. Briefly, lentiviral particles were packaged in 293FT cells utilizing the psPAX2 (Addgene, 12,260) and pMD2.G (Addgene, 12,259) packing plasmids. Virus titers were determined by Lenti-Pac HIV qRT-PCR Titration Kit (Genecopoeia, LT005). A375, SK-MEL-28, and SK-MEL-2 cells were separately transduced the lentiviruses at a multiplicity of infection (MOI) of 0.1 for 24 h and selected with 6 or 9 µg/ml blasticidin (ThermoFisher, 461,120) for 5 days, and then expanded cultivation. To perturb specific enhancers, we cloned the most significant enriched sgRNA DNA sequences into the lentiGuide-Puro (Addgene, 52,963) plasmid, respectively, each recombinant plasmid and empty vector were packaged into lentivirus as mentioned above. Then, we separately transduced the recombinant lentiviruses and empty lentivirus at an MOI of 10 into A375-KRAB, SK-MEL-28-KRAB, SK-MEL-2-KRAB, A375-VP64, SK-MEL-28-VP64, and SK-MEL-2-VP64 cells, and then expanded cultivation after selecting with 1 or 2 µg/ml puromycin (Sigma-Aldrich, P7255) for 4 days. For enhancer knockout assay, sgRNAs for E349 or E156 were designed by the CRISPOR (v4.99) Web Portal [104], and then separately cloned into the pGL3-U6-sgRNA-EGFP plasmid (Addgene, 107721). Single cell-derived stable A375-Cas9 cell line was generated using the LentiCas9-Blast (Addgene, 52962) vector as mentioned above. The recombinant plasmids (couple plasmids for each enhancer) were then separately transfected into the single cell-derived A375-Cas9 cells using the Lipofectamine 2000 transfection reagent. After 48 h, the transfected cells were digested and diluted into 50–100 cells/100 µl, and inoculated into each well of 96-well plates. After expanding culture of the cells from 96-well plates to 24-well plates, genomes of single-derived GFP-positive cells were extracted using a commercial DNA isolation kit (QIAGEN, 51304). The sequences harboring the edited loci were amplified with the Premix Taq enzyme, and the products were subsequently sent to BGI for Sanger sequencing. The sequencing results were analyzed and visualized on the CRISP-ID Web Portal [105] or SnapGene Viewer (v4.3.10) software. Primers and oligonucleotide sequences used are listed in Additional file 15: Table S14.

### Western blotting and quantitative real-time RT-PCR (RT-qPCR)

Anti-Human MEF2A Antibody (1:1000; Abcam, ab76063), Anti-Human PTEN Antibody (1:1000; Cell Signaling Technology, 9188 T), Anti-Human phosphor-AKT Antibody (1:2000; Cell Signaling Technology, 4060), Anti-Human total AKT Antibody (1:1000; Cell Signaling Technology, 4691), Anti-Human KLLN Antibody (1:1000; Abcam, ab197892), Anti-human ATAD1 Antibody (1:1000; CUSABIO, CSB-PA850855LA01HU), Anti-human IGF1R Antibody (1:1000; Abcam, ab182408), Anti-Human β-actin Antibody (1:200,000; ABclonal, AC026), and Anti-human GAPDH Antibody (1:100,000; ABclonal, AC001) were used to perform western blotting. Western blotting was performed as previously described [106]. For western blotting analysis, the gray value of protein bands was measured by ImageJ2 (https://imagej.net/ImageJ). The ratio of gray value of the target protein to that of the housekeeping protein was calculated and then normalized to control. For RT-qPCR, total RNA in cells was extracted utilizing TRIzol (ThermoFisher, 15596026) and reverse-transcribed using SuperScript III Reverse Transcriptase (ThermoFisher, 12574026). Each RT-qPCR was performed with approximately 200 ng of DNase-treated RNA using the SYBR Premix Ex Taq II (TaKaRa, RR820A). The relative expression of gene was calculated using the 2^−ΔΔCt^ method, and fold changes were calculated as described in the figure legends. Error bars represent the standard deviations (SDs) of the average fold changes based on three experimental duplicates as indicated the figure legends. Primers used are listed in Additional file 15: Table S14.

### Cell proliferation assay

A375-KRAB infected with the empty lentivirus, enhancer-inhibited A375-KRAB, SK-MEL-28-KRAB infected with the empty lentivirus, enhancer-inhibited SK-MEL-28-KRAB, SK-MEL-2-KRAB infected with the empty lentivirus, and enhancer-inhibited SK-MEL-2-KRAB cells were separately seeded 100 µl into 96-well microplates with 3000 cells per chamber, and four replicates were performed for each group. After 24 h, culture medium with 1 or 2 µM vemurafenib or no treatment was added into each chamber. Cells were further incubated for 24, 48, 60, and 72 h, and cell viability was detected using the CCK-8 Cell Counting Kit (Vazyme, A311-01) according to the manufacturer’s description. Briefly, the original culture medium in the 96-well microplates was discarded, 100 µl of 10% CCK-8-DMEM high glucose solution was added into each chamber, and then the microplates were incubated at 37 °C for 1 h. The absorbance was measured at 450 nm wave length by a microplate reader (Molecular devices, SpectraMax M). Viability was calculated as a percentage of control (A375-KRAB, SK-MEL-28-KRAB, or SK-MEL-2-KRAB cells without treatment) after background subtraction.

### Apoptosis assay

A375-KRAB infected with the empty lentivirus, enhancer-inhibited A375-KRAB, SK-MEL-28-KRAB infected with the empty lentivirus, enhancer-inhibited SK-MEL-28-KRAB, SK-MEL-2-KRAB infected with the empty lentivirus, and enhancer-inhibited SK-MEL-2-KRAB cells were separately seeded 2 ml into 6-well plates at a density of 2 × 10^5^ cells/ml, and three replicates were performed for each group. After 24 h, cells were added 1 µM vemurafenib or no treatment and then incubated in an incubator with the atmosphere of 5% CO_2_ at 37 °C for 2 days. Adherent cells were digested into single cells using No-EDTA Trypsin (ThermoFisher, 15050065) and stained with Annexin V-FITC/propidium iodide (PI) Apoptosis Detection Kit (Vazyme, A211-02) according to the manufacturer’s description. Briefly, the harvested cells were washed two times with pre-cooled PBS and incubated with 5 µl FITC-labeled Annexin V and 5 µl PI Staining Solution at room temperature for 10 min in the dark room. Early apoptotic (Annexin V-FITC stained only) and late apoptotic (Annexin V-FITC and PI double-stained) cells were detected by flow cytometry (BD Biosciences, USA), and the results were analyzed and visualized with FlowJo (FlowJo LLC.).

### Flat colony formation assay

A375-KRAB infected with the empty lentivirus, enhancer-inhibited A375-KRAB, SK-MEL-28-KRAB infected with the empty lentivirus, enhancer-inhibited SK-MEL-28-KRAB, SK-MEL-2-KRAB infected with the empty lentivirus, and enhancer-inhibited SK-MEL-2-KRAB cells were separately seeded 2 ml in 6-well plates with 500 cells for A375-derived or SK-MEL-2-derived cells and 1000 cells for SK-MEL-28-derived cells per chamber, and three replicates were performed for each group. Each chamber was added 1 µM vemurafenib or no treatment in a 2-ml culture system, and incubated in an incubator with the atmosphere of 5% CO_2_ at 37 °C. Vemurafenib was replaced every 3 days. After 12 days, the culture supernatant was discarded and washed two times with PBS, and then 4% paraformaldehyde (ThermoFisher, R37814) was added and fixed for 15 min. The stationary solution was discarded, and the plates were washed two times with PBS. The number of colonies was counted and photographed with camera after staining with 0.5% crystal violet (ThermoFisher, R40052) for 30 min and washed extensively with sterilized distilled water.

### Cell-derived xenograft (CDX)

A375-KRAB infected with the empty lentivirus and enhancer-inhibited A375-KRAB melanoma cells were used for the CDX. Briefly, 1 × 10^6^ viable cells were resuspended in 100 µl DMEM and subcutaneously injected into the armpit of 5-week-old female nude mice (C57BL/6 background). At 14 or 21 days after injection, the tumor volumes were detected and analyzed by a high-resolution ultrasound imaging system for small animals (FUJIFILM VisualSonics, model Vevo 3100) according to the manufacturer’s instructions. Above 3 weeks after tumor cell inoculation, when the tumors reached a volume of 150–200 mm^3^, the nude mice were killed by cutting their necks, and the tumor tissues were harvested and macro dissected to minimize the content of necrotic tissue. The acquired tumors were weighed by an electronic balance (METTLER TOLEDO, model ME204E).

### Immunohistochemistry (IHC)

Paraffin-embedded melanoma samples of two patients with E_349 mutation were retrieved from the tissue sample library of Tianjin Medical University Cancer Institute and Hospital. The IHC assay was used to detect the expression of MEF2A and Cell proliferation-related proteins in paraffin-embedded E_349 deletion or extensive mutations melanoma tissues and matched control melanoma tissues. The slices were heated, dewaxed, rehydrated, and put into sodium citrate buffer (pH buffer = 6.0; ThermoFisher, 005000) for antigen repair. The slide was then soaked in 3% hydrogen peroxide (H_2_O_2_; ThermoFisher, 241020010) to inhibit endogenous peroxidase activity. After rinsing three times with PBS, the slices were incubated overnight with the first antibody, including rabbit or mouse antibodies against MEF2A (1:800; Affinity, AF6381) or Ki-67 (1:800; Abcam, ab16667) at 4 °C, and antibody diluention buffer was used the Superkine Enhanced Antibody Dilution Buffer (Abbkine, BMU103). Slices were washed three times using PBS and then treated with a second antibody including anti-rabbit IgG (1:2000, Cell Signal Technology, 7074) or anti-mouse IgG (1:2000, Cell Signal Technology, 7076) for 40 min at 37 °C. After being stained with Diaminobenzidine (DAB; Vector Laboratories, Cat# SK-4100), it was stained with hematoxylin (Sigma, H3136), dehydrated, sealed, and visualized under a microscopy.

### Statistical analyses

Statistical analyses were carried out with GraphPad Prism 8.0 (GraphPad Software). All experiments were performed at least 3 replicates, unless otherwise noted. Differences in means were compared using an unpaired two-tailed Student’s *t* test, and graphed as the means ± standard deviations (SD) or means ± standard error of mean (SEM). Statistical significance denoted as follows: *, *p* < 0.05; **, *p* < 0.01; ***, *p* < 0.001; ****, *p* < 0.0001.

### Supplementary Information

**Additional file 1: Fig S1.** Workflow of genome-wide HRR identification based on melanoma WGS data. **Fig S2.** Reproducibility and annotation of functional HRR-associated enhancer in CRISPRi screen.** Fig S3.** CRISPRi screen day7 results on A375 and SK-MEL-2 cells. **Fig S4.** Mutational signature analysis for HRR-associated enhancers.** Fig S5.** 3C assay results between the *MEF2A* promoter and E_349. **Fig S6.** Survival evidence for alternative target genes of the top functional enhancers in TCGA-SKCM patients.** Fig S7.** E_349 modulates melanoma cell proliferation and apoptosis by targeting *MEF2A*.** Fig S8.** 3C assay results between the *PTEN* promoter and E_156.** Fig S9.** Distal enhancer-sustaining *PTEN* tumor-suppressive potential in melanoma cells.** Fig S10.** Detection the function of E_349 or E_156 on their predicted genes.** Fig S11.** Identification the knockout of E_349 or E_156 in A375 cells.** Fig S12.** Immunohistochemistry (IHC) results of melanoma patient samples with E_349 deletion or extensive mutations.

**Additional file 2: Table S1.** Integrated clinical information of 297 melanoma patients from ICGC.

**Additional file 3: Table S2.** Summary information and supporting evidence of significant SNV/INDEL-HRRs in melanoma.

**Additional file 4: Table S3.** Summary information and supporting evidence of prioritized SV-HRRs in melanoma.

**Additional file 5: Table S4.** Curated melanoma essential candidate genes from high-throughput screening.

**Additional file 6: Table S5.** Information of sgRNAs and associated enhancers.

**Additional file 7: Table S6.** sgRNA count table of CRISPRi screen for A375 and SK-MEL-2 cells.

**Additional file 8: Table S7.** Significant enhancers of CRISPRi screen identified by MAGeCK.

**Additional file 9: Table S8.** Epigenetic annotations for significant functional enhancers.

**Additional file 10: Table S9.** The presence of functional enhancers in different cell lines.

**Additional file 11: Table S10.** H3k27ac signal of patients captured by enhancers.

**Additional file 12: Table S11.** Melanoma cell lines genetic profile in enhancer regions.

**Additional file 13: Table S12.** A375 H3K27ac HiChIP interactions identified by FitHiChIP.

**Additional file 14: Table S13.** Target gene annotation and supporting evidence functional enhancers in melanoma.

**Additional file 15: Table S14.** Primers and oligonucleotide sequences used in the experiments.

**Additional file 16.** Source data including uncropped WB and gel images.

**Additional file 17.** Review history.

## Data Availability

Raw CRISPRi screening, H3K27ac HiChIP and 4C-seq data in this study were deposited in the GEO database under accession number GSE210424 [107]. CRISPRi screening sgRNA counts and all primer sequences used in this study are listed in Additional files. Raw WGS data for 297 paired melanoma tumor and normal samples (MELA-AU: 183 samples, SKCM-US: 38 samples, and SKCA-BR: 76 samples) were downloaded from the International Cancer Genome Consortium (ICGC) data portal (https://dcc.icgc.org/repositories) and European Genome-phenome Archive (EGAD00001003388). H3K27ac ChIP-seq data from the 10 melanoma cell lines were downloaded from the GEO database under accession number GSE143653, GSE99835 and GSE82332. A375 epigenomic data were downloaded from the GEO database, including CTCF (GSM3671692), H3K4me3 (GSM2653904), Pol II ChIP-seq (GSM1661790), and PRO-seq (GSE128081). A375 Hi-C data was acquired from the GEO database under accession number GSE143676. HiChIP loop data for WM (GSM5680738) and COLO (GSM5680737) cell lines were downloaded from the GEO database.
