# Supplementary material for: Landscape of enhancer disruption and functional screen in melanoma cells
Source: Genome Biol. 2023 Oct 30;24:248. doi: 10.1186/s13059-023-03087-5 (PMC10614365; doi:10.1186/s13059-023-03087-5)
Supplement: Supplementary file 1 — Additional file 1: Fig S1. Workflow of genome-wide HRR identification based on melanoma WGS data. Fig S2. Reproducibility and annotation of functional HRR-associated enhancer in CRISPRi screen. Fig S3. CRISPRi screen day7 results on A375 and SK-MEL-2 cells. Fig S4. Mutational signature analysis for HRR-associated enhancers. Fig S5. 3C assay results between the MEF2A promoter and E_349. Fig S6. Survival evidence for alternative target genes of the top functional enhancers in TCGA-SKCM patients. Fig S7. E_349 modulates melanoma cell proliferation and apoptosis by targeting MEF2A. Fig S8. 3C assay results between the PTEN promoter and E_156. Fig S9. Distal enhancer-sustaining PTEN tumor-suppressive potential in melanoma cells. Fig S10. Detection the function of E_349 or E_156 on their predicted genes. Fig S11. Identification the knockout of E_349 or E_156 in A375 cells. Fig S12. Immunohistochemistry (IHC) results of melanoma patient samples with E_349 deletion or extensive mutations. [file 13059_2023_3087_MOESM1_ESM.pdf]

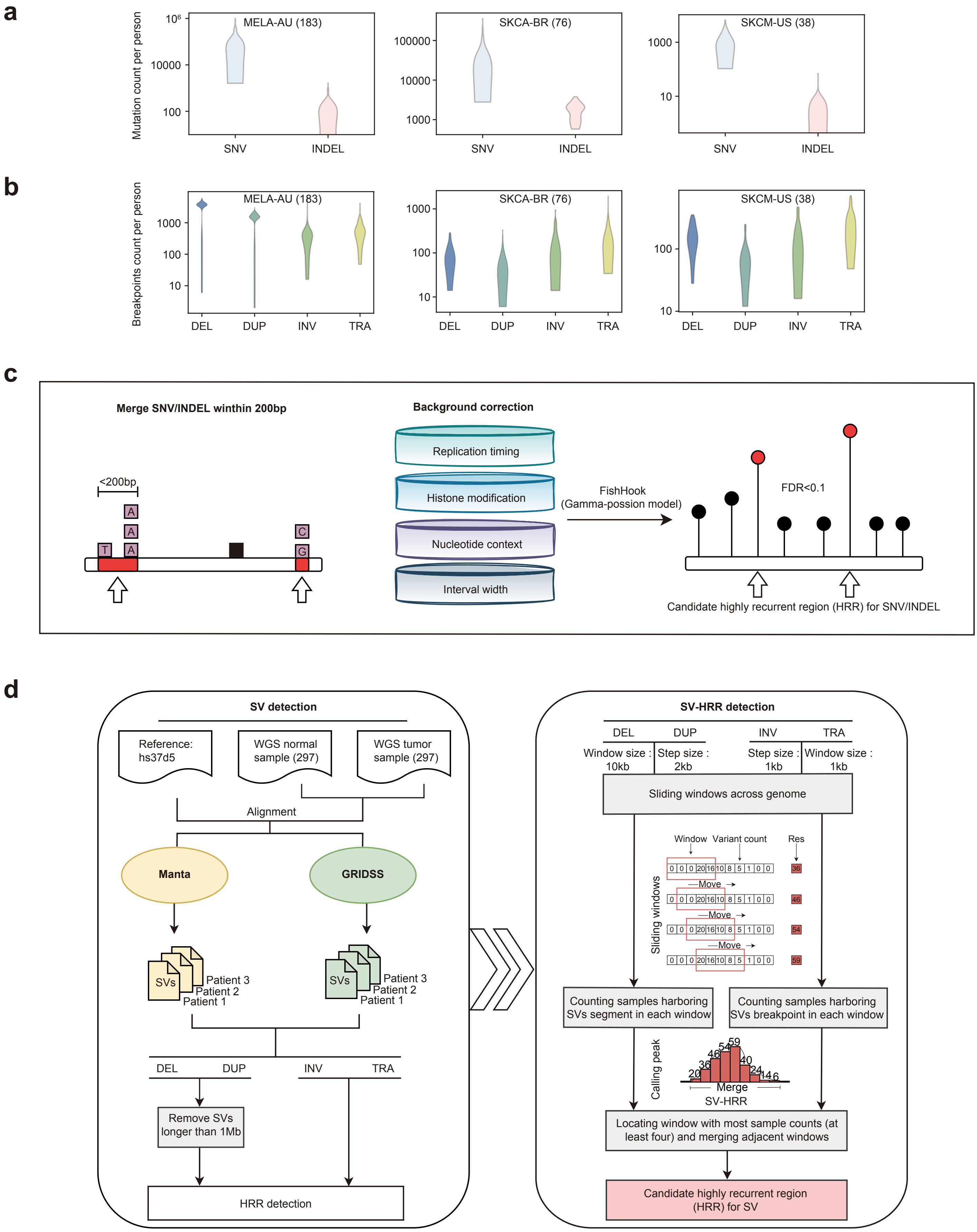
a
Mutation count per person
MELA-AU (183)
SKCA-BR (76)
SKCM-US (38)
SNV
INDEL
b
Breakpoints count per person
DEL
DUP
INV
TRA
c
Merge SNV/INDEL winthin 200bp
<200bp
Background correction
Replication timing
Histone modification
Nucleotide context
Interval width
FishHook
(Gamma-possion model)
FDR<0.1
Candidate highly recurrent region (HRR) for SNV/INDEL
d
SV detection
Reference: hs37d5
WGS normal sample (297)
WGS tumor sample (297)
Alignment
Manta
GRIDSS
SVs
Patient 3
Patient 2
Patient 1
DEL
DUP
INV
TRA
Remove SVs longer than 1Mb
HRR detection
SV-HRR detection
Window size : 10kb
Step size : 2kb
Step size : 1kb
Window size : 1kb
Sliding windows across genome
Window
Variant count
Res
Move
Sliding windows
Counting samples harboring SVs segment in each window
Counting samples harboring SVs breakpoint in each window
Calling peak
Merge
SV-HRR
Locating window with most sample counts (at least four) and merging adjacent windows
Candidate highly recurrent region (HRR) for SV

Additional file1: Fig. S1 Workflow of genome-wide HRR identification based on melanoma WGS data. a Burden of somatic SNV/INDELs in three integrated melanoma WGS projects. **b** Burden of somatic SVs in three integrated melanoma WGS projects. **c** Schematic diagram of the SNV/INDEL-HRR discovery pipeline. **d** Schematic diagram of the SV-HRR prioritization pipeline. HRR-associated genes those have potential contribution to melanoma support by genetic evidence, functional screen evidence, literature mining evidence were labeled in right table.

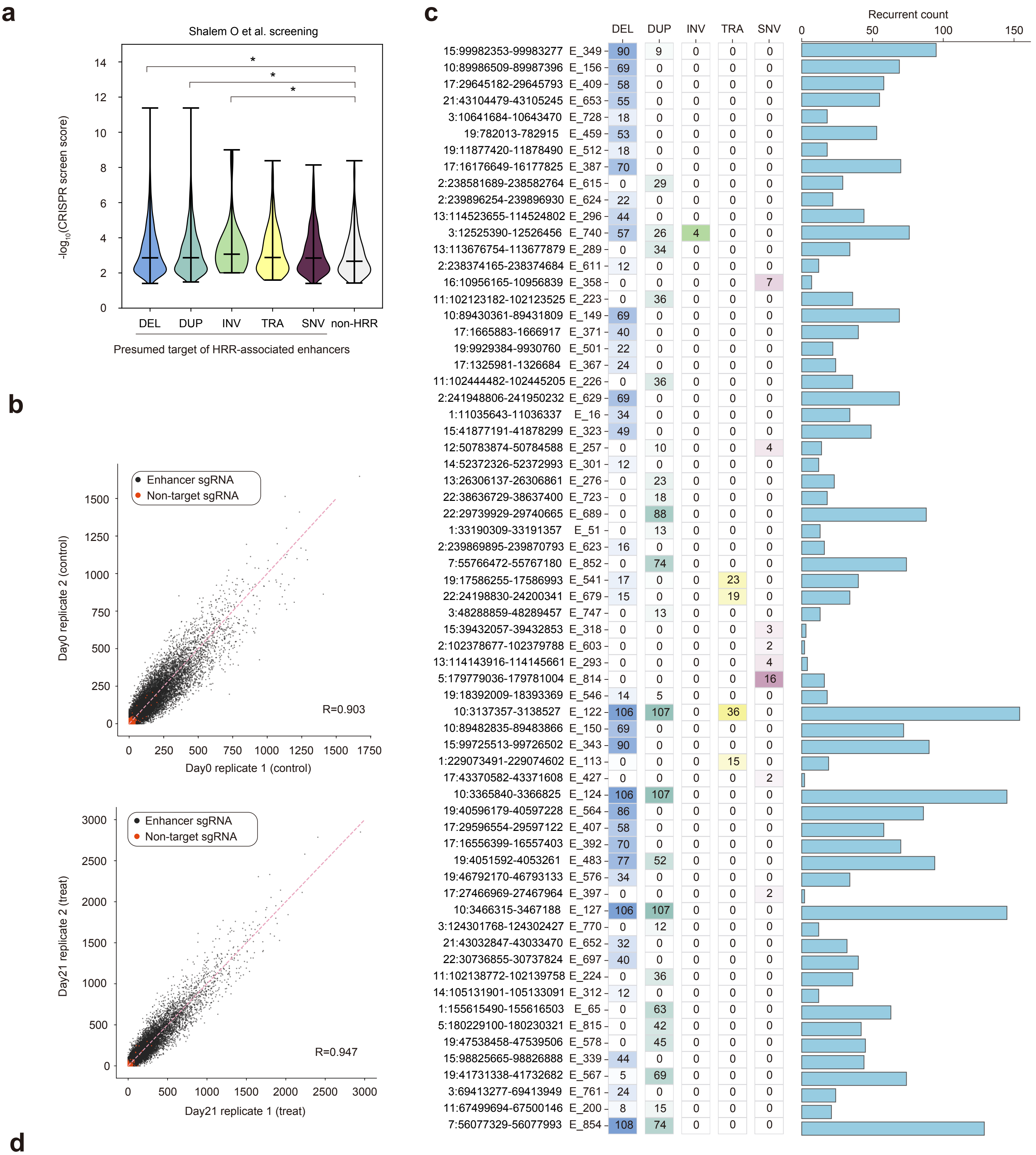

d

ID	enhancer location	neg|fdr	neg|lfc	variant	target gene
E_394	17:16918009-16918495	0.001068	-1.2088	DEL	FLCN, COPS3, MPRIP
E_497	19:5985341-5985792	0.001068	-0.73503	DUP	CAPS, CD70, RANBP3
E_804	5:1792880-1793417	0.001068	-1.139	DEL	
E_376	17:6655806-6656127	0.001068	-2.0114	DUP	SLC16A13, BCL6B, C17orf49, RNASEK, ALOX12
E_545	19:17976074-17977267	0.023504	-0.87705	SNV	IFI30, PIK3R2, MAST3, ARRDC2, KCNN1, CCDC124, B3GNT3, FCHO1, MAP1S, UNC13A, PGLS, SLC27A1, NXNL1 COLGALT1, BST2, GTPBP3, ANO8, MVB12A
E_763	3:69836247-69837207	0.023504	-0.70371	DUP	MITF
E_379	17:7677267-7678089	0.027473	-0.30255	DEL	DNAH2, KDM6B, TMEM88
E_351	15:100026314-100027579	0.041667	-0.86045	DEL	
E_677	22:21137931-21138863	0.041667	-0.59227	DUP	SERPIND1, PI4KA
E_318	15:39437940-39438530	0.041667	-0.47847	SNV	THBS1, FSIP1
E_530	19:13947666-13948172	0.041667	-0.30886	DUP	JUNB, HOOK2, DDX39A, PKN1, DNAJB1, TECR, PODNL1, DCAF15, RFX1, IL27RA, PALM3, C19orf67, SAMD1, PRKACA, GIPC1, NDUFB7, CLEC17A, CC2D1A, RLN3, NANOS3, ASF1B, C19orf53, PRDX2, RNASEH2A, RTBDN, MAST1, BEST2, GCDH, ZSWIM4, FARSA, CALR, RAD23A, SYCE2, DAND5, GADD45GIP1, CACNA1A, IER2, STX1[illegible] MRI1, TRMT1, LYL1, NFIX, NACC1, KLF1
E_50	1:33182249-33182827	0.041667	-0.1471	DUP	
E_684	22:29168330-29169614	0.047009	-0.79465	DEL	XBP1, HSCB, CHEK2
E_871	7:140325981-140326709	0.047009	-0.27918	DUP, TRA	
E_75	1:156163289-156164433	0.047009	-0.14247	DUP	SEMA4A, LMNA, PMF1
E_637	20:35112090-35112733	0.047009	-0.7174	DEL	

Additional file1: Fig. S2 Reproducibility and annotation of functional HRR-associated enhancer in CRISPRi screen. a Functional evaluation of HRR-associated enhancer in A375 public CRISPR KO negative selection screen. Box plot represents the difference of CRISPR enrichment scores between the presumed genes in HRR-associated enhancer group across five HRR types and those in HRR-unrelated enhancer group, Mann-Whitney U Test (p-value DEL = 0.0087; p-value DUP = 0.0194; p-value SNV = 0.014). **b** Reproducibility analysis of CRISPRi screens. Enhancer-targeting sgRNAs (black) and non-targeting sgRNA sgRNAs (red) are shown. The x- and y-axis denote the normalized counts of sgRNA in day 0 (control) and day 21 (treatment) samples. The Pearson correlation coefficient (R) values are shown. **c** Annotation of the 66 significant functional enhancers. Heatmap represents recurrence of different HRR types among 297 melanoma patients corresponding to each functional enhancer. The overall recurrence of enhancer-located HRR is shown on the right bar. **d** Enhancers and target genes identified by HiChIP in negative screen with FDR < 0.05.

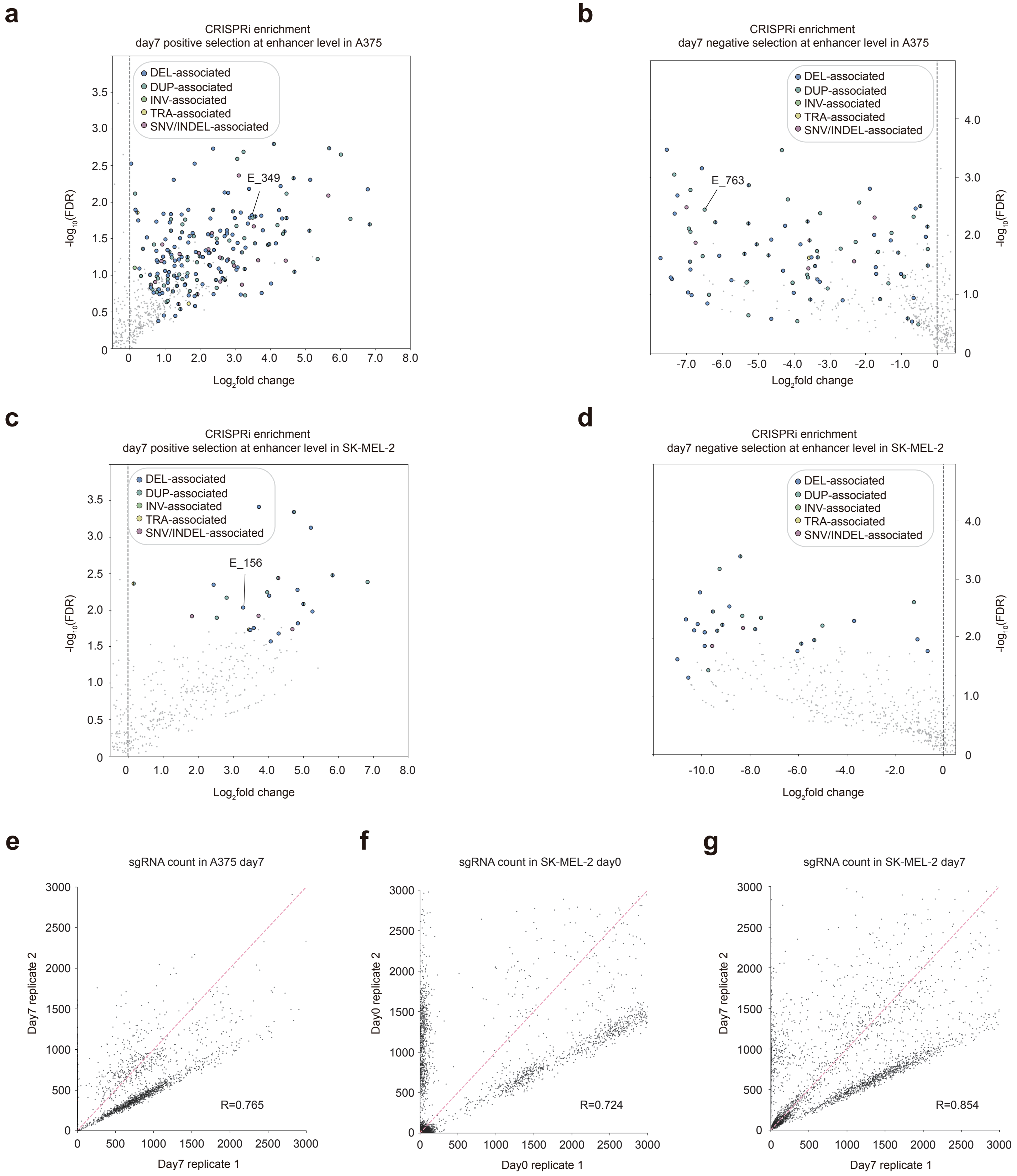

a
CRISPRi enrichment
day7 positive selection at enhancer level in A375
DEL-associated
DUP-associated
INV-associated
TRA-associated
SNV/INDEL-associated
E_349
-log10(FDR)
Log2fold change
b
CRISPRi enrichment
day7 negative selection at enhancer level in A375
DEL-associated
DUP-associated
INV-associated
TRA-associated
SNV/INDEL-associated
E_763
-log10(FDR)
Log2fold change
c
CRISPRi enrichment
day7 positive selection at enhancer level in SK-MEL-2
DEL-associated
DUP-associated
INV-associated
TRA-associated
SNV/INDEL-associated
E_156
-log10(FDR)
Log2fold change
d
CRISPRi enrichment
day7 negative selection at enhancer level in SK-MEL-2
DEL-associated
DUP-associated
INV-associated
TRA-associated
SNV/INDEL-associated
-log10(FDR)
Log2fold change
e
sgRNA count in A375 day7
Day7 replicate 2
Day7 replicate 1
R=0.765
f
sgRNA count in SK-MEL-2 day0
Day0 replicate 2
Day0 replicate 1
R=0.724
g
sgRNA count in SK-MEL-2 day7
Day7 replicate 2
Day7 replicate 1
R=0.854

Additional file1: Fig. S3 CRISPRi screen day7 results on A375 and SK-MEL-2 cells. a,b CRISPRi positive and negative screen in A375 cells. MAGeCK-RRA was performed on day7 group versus day0 group, each group contains 2 replicates. 196 enhancers were screened out in positive with p-value < 0.05 (**a**). 87 enhancers were screened out in negative with p-value < 0.05 (**b**). The middle table shows the enhancers significantly enriched in day7 screen that also as hits in A375 day21 screen, 14 of which is positive, and two is negative. We labelled the enhancer in the scatter plot, which was either validated in the experiment, or has a classic melanoma target gene. **c,d** CRISPRi positive and negative screen in SK-MEL-2 cells, the replicates of each sample and screening method is same as above. 25 enhancers were screened out in positive selection (**c**), and 29 were in negative selection (**d**) with p-value < 0.05. The middle table document the consistency between A375 day7 screened hits above and day21 hits. The enhancer we had experimentally validated was labelled. **e,f,g** Reproducibility analysis of CRISPRi screens in samples of A375 day7 (**e**), SK-MEL-2 day0 (**f**), SK-MEL-2 day7 (**g**), respectively. The Pearson correlation coefficient (R) values are shown.

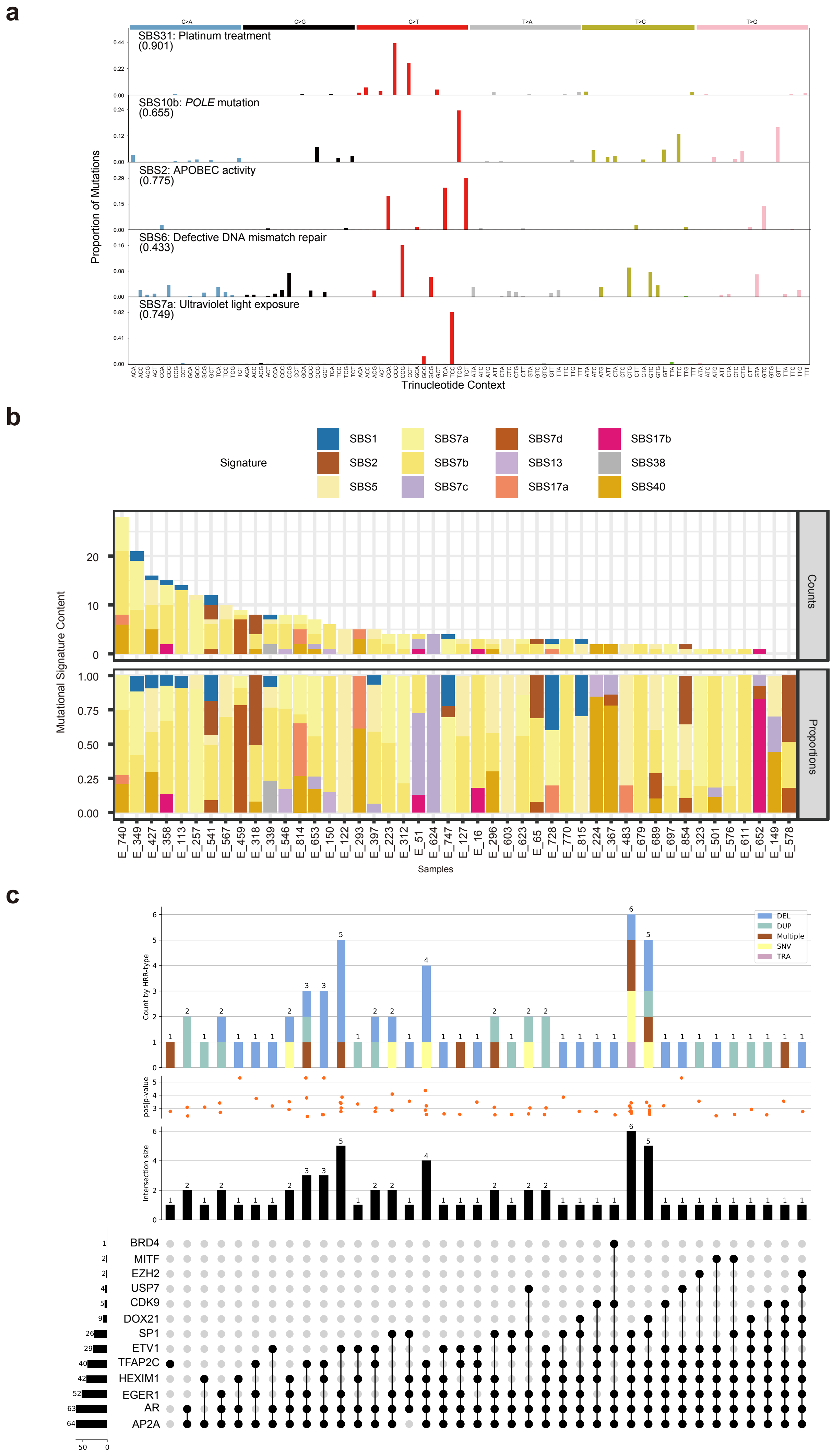
a
C>A
C>G
C>T
T>A
T>C
T>G
SBS31: Platinum treatment
(0.901)
SBS10b: *POLE* mutation
(0.655)
SBS2: APOBEC activity
(0.775)
SBS6: Defective DNA mismatch repair
(0.433)
SBS7a: Ultraviolet light exposure
(0.749)
Proportion of Mutations
Trinucleotide Context
b
Signature
SBS1
SBS2
SBS5
SBS7a
SBS7b
SBS7c
SBS7d
SBS13
SBS17a
SBS17b
SBS38
SBS40
Mutational Signature Content
Counts
Proportions
Samples
E_740
E_349
E_427
E_358
E_113
E_257
E_541
E_567
E_459
E_318
E_339
E_546
E_814
E_653
E_150
E_122
E_293
E_397
E_223
E_312
E_51
E_624
E_747
E_127
E_16
E_296
E_603
E_623
E_65
E_728
E_770
E_815
E_224
E_367
E_483
E_679
E_689
E_697
E_854
E_323
E_501
E_576
E_611
E_652
E_149
E_578
c
DEL
DUP
Multiple
SNV
TRA
Count by HRR-type
pos|p-value
Intersection size
BRD4
MITF
EZH2
USP7
CDK9
DOX21
SP1
ETV1
TFAP2C
HEXIM1
EGER1
AR
AP2A

Additional file1: Fig. S4 Mutational signature analysis for HRR-associated enhancers. **a** *De novo* mutational signature analysis of all 4,645 HRR-associated enhancers. Cosine similarity values with COSMIC mutational signatures are shown. **b** The contribution of the known melanoma COSMIC SBS signatures in 66 functional enhancers. The number (top) and proportion (bottom) of mutations attributed to each mutational signature in each enhancer is colored by signature types.

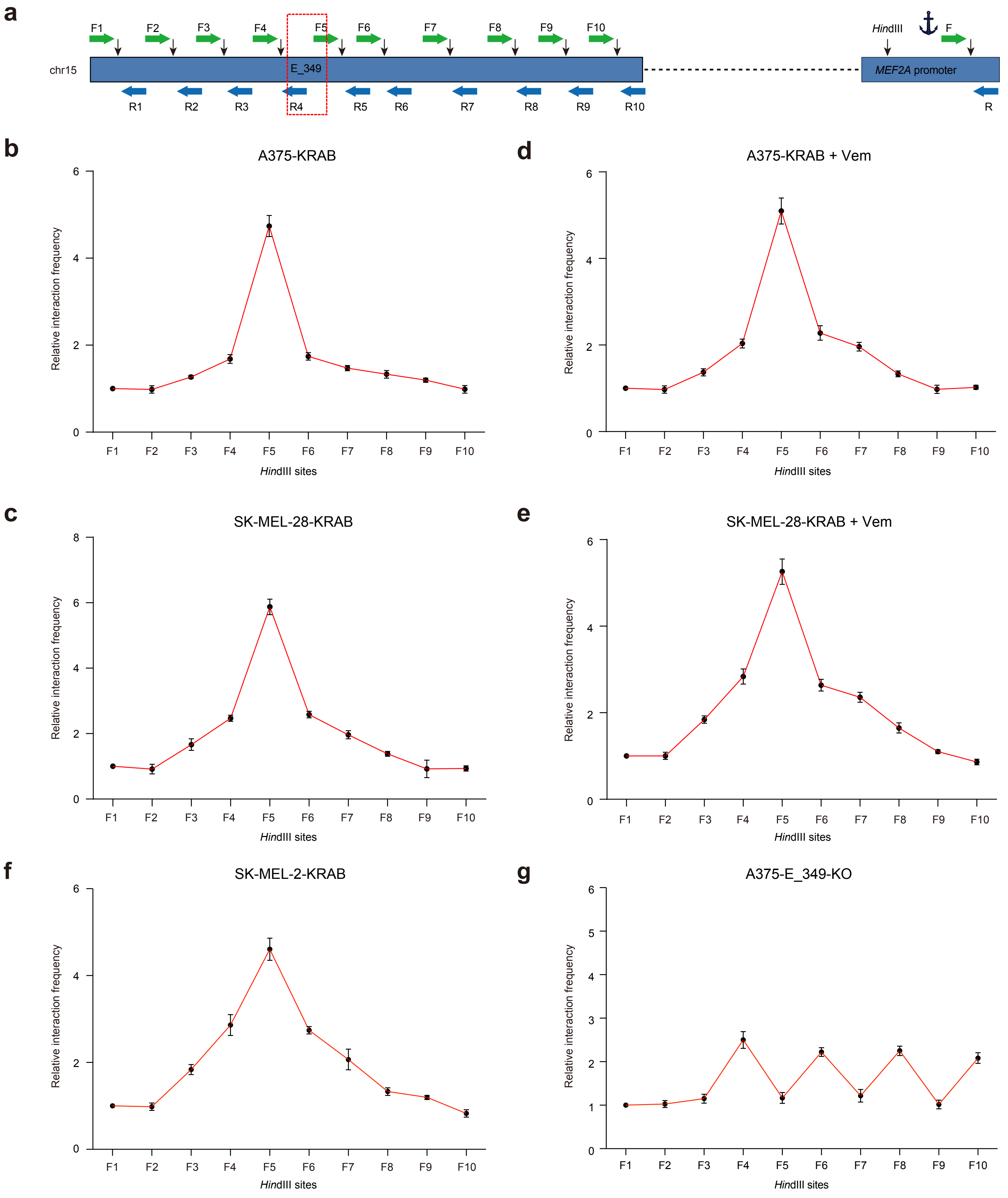
a
chr15
E_349
MEF2A promoter
HindIII
F1 F2 F3 F4 F5 F6 F7 F8 F9 F10 F
R1 R2 R3 R4 R5 R6 R7 R8 R9 R10 R
b
A375-KRAB
c
SK-MEL-28-KRAB
d
A375-KRAB + Vem
e
SK-MEL-28-KRAB + Vem
f
SK-MEL-2-KRAB
g
A375-E_349-KO
Relative interaction frequency
HindIII sites

Additional file1: Fig. S5 3C assay results between the *MEF2A* promoter and E_349. **a** A schematic view of 3C assays at the chromosome region surrounding the *MEF2A* promoter. The *Hin*dIII-cutting fragment containing the *MEF2A* promoter (GRCh37/hg19 chr15: 100,123,291–100,129,474) is measured as a bait indicated by the anchor, and a series of fragments upstream of the *MEF2A* promoter is measured as preys. Blue rectangles denote the chromosome, black arrows denote the *Hin*dIII-cutting sites, and green arrows denote the location and direction of primers used in 3C assay. Blue arrows denote the reverse primer used in amplifying the control fragments. **b**,**c** 3C assay results of interaction between the *MEF2A* promoter and E_349 in A375-KRAB (**b**) and SK-MEL-28-KRAB (**c**) cells (n = 3 samples). **d**,**e** 3C assay results of interaction between the *MEF2A* promoter and E_349 in A375-KRAB (**d**) and SK-MEL-28-KRAB (**e**) cells under treatment with 1 μM vemurafenib (n = 3 samples). **f**,**g** 3C assay results of interaction between the *MEF2A* promoter and E_349 in SK-MEL-2-KRAB (**f**) and E_156-KO A375 (**g**) cells (n = 3 samples).

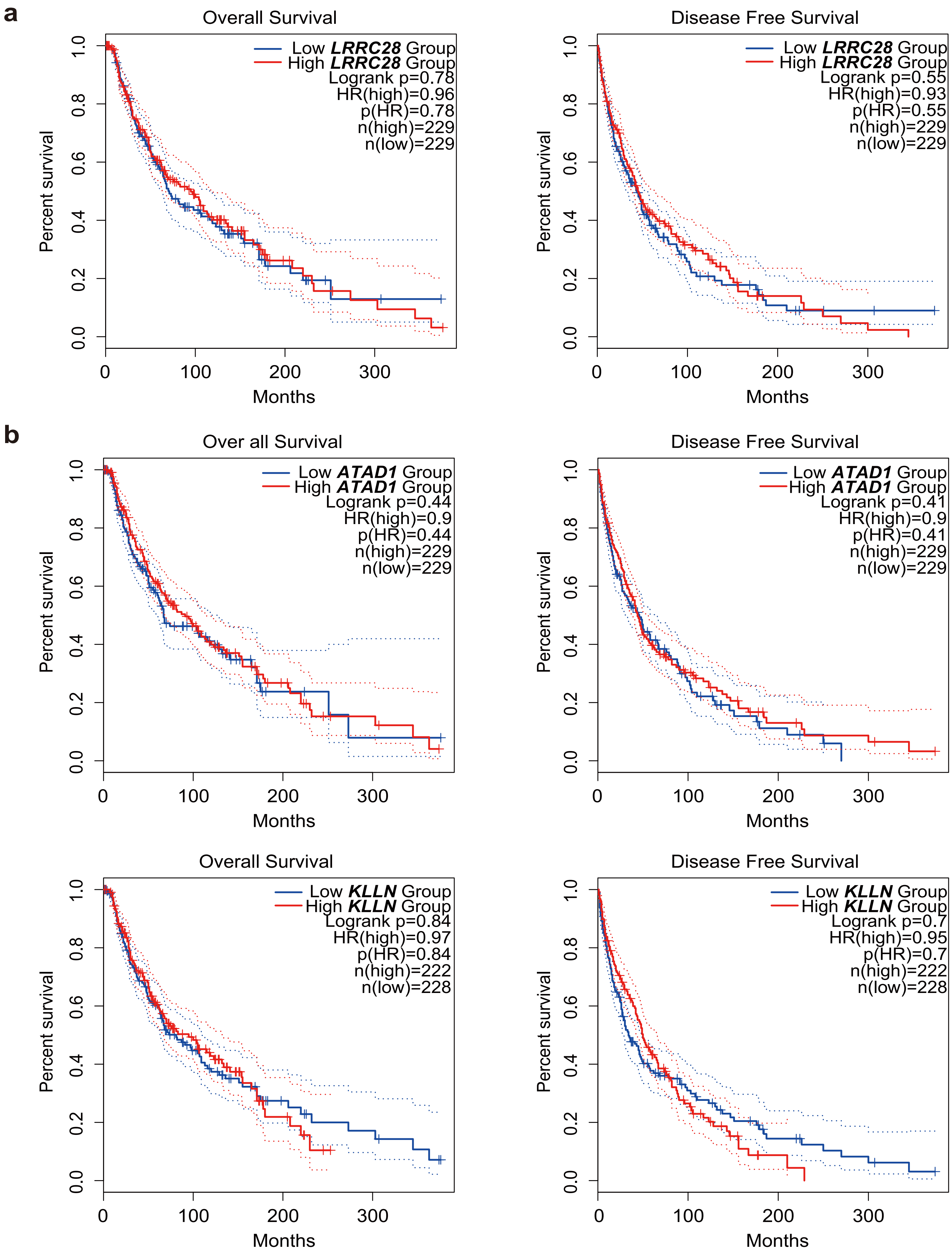
a
Overall Survival
Low LRRC28 Group
High LRRC28 Group
Logrank p=0.78
HR(high)=0.96
p(HR)=0.78
n(high)=229
n(low)=229
Percent survival
Months
Disease Free Survival
Low LRRC28 Group
High LRRC28 Group
Logrank p=0.55
HR(high)=0.93
p(HR)=0.55
n(high)=229
n(low)=229
b
Over all Survival
Low ATAD1 Group
High ATAD1 Group
Logrank p=0.44
HR(high)=0.9
p(HR)=0.44
n(high)=229
n(low)=229
Disease Free Survival
Low ATAD1 Group
High ATAD1 Group
Logrank p=0.41
HR(high)=0.9
p(HR)=0.41
n(high)=229
n(low)=229
Overall Survival
Low KLLN Group
High KLLN Group
Logrank p=0.84
HR(high)=0.97
p(HR)=0.84
n(high)=222
n(low)=228
Disease Free Survival
Low KLLN Group
High KLLN Group
Logrank p=0.7
HR(high)=0.95
p(HR)=0.7
n(high)=222
n(low)=228

Additional file1: Fig. S6 Survival evidence for alternative target genes of the top functional enhancers in TCGA-SKCM patients. a Expression of alternative target gene *LRRC28* of E_349 shows no relevance with clinical outcome. **b** Expression of alterative target genes *ATAD1* and *KLLN* of E_156 show no relevance with clinical outcome.

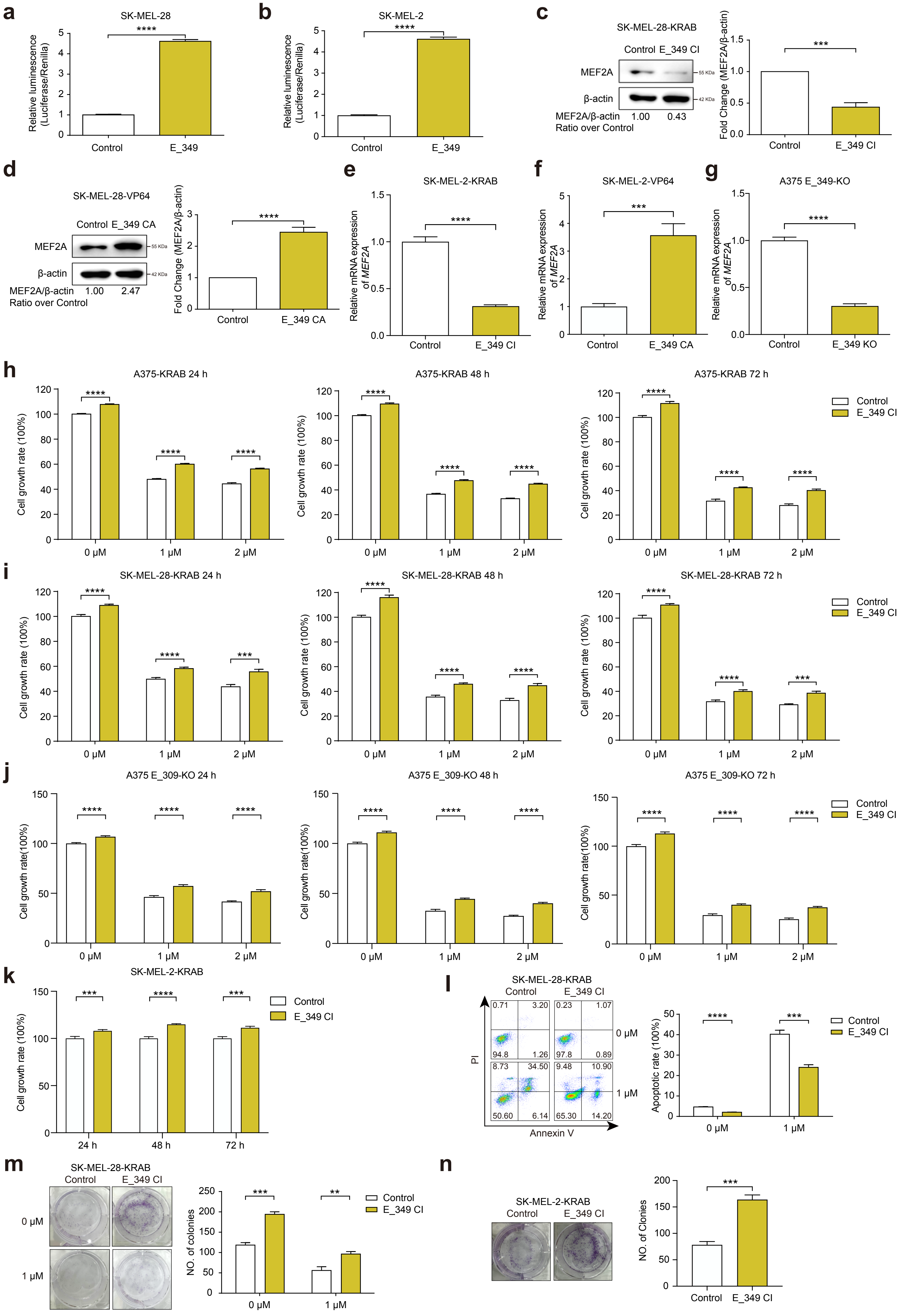
a
SK-MEL-28
Relative luminescence (Luciferase/Renilla)
Control
E_349
b
SK-MEL-2
c
SK-MEL-28-KRAB
Control E_349 CI
MEF2A
β-actin
MEF2A/β-actin 1.00 0.43
Ratio over Control
Fold Change (MEF2A/β-actin)
E_349 CI
d
SK-MEL-28-VP64
Control E_349 CA
MEF2A/β-actin 1.00 2.47
E_349 CA
e
SK-MEL-2-KRAB
Relative mRNA expression of MEF2A
f
SK-MEL-2-VP64
g
A375 E_349-KO
E_349 KO
h
A375-KRAB 24 h
A375-KRAB 48 h
A375-KRAB 72 h
Cell growth rate (100%)
0 μM
1 μM
2 μM
i
SK-MEL-28-KRAB 24 h
SK-MEL-28-KRAB 48 h
SK-MEL-28-KRAB 72 h
j
A375 E_309-KO 24 h
A375 E_309-KO 48 h
A375 E_309-KO 72 h
Cell growth rate(100%)
k
SK-MEL-2-KRAB
24 h
48 h
72 h
l
SK-MEL-28-KRAB
PI
Annexin V
Apoptotic rate (100%)
m
SK-MEL-28-KRAB
NO. of colonies
n
SK-MEL-2-KRAB
NO. of Clonies

Additional file1: Fig. S7 E_349 modulates melanoma cell proliferation and apoptosis by targeting *MEF2A*. a,b Luciferase assay results of the E_349 core region in SK-MEL-28 (**a**) and SK-MEL-2 (**b**) cells. **c**,**d** Western blotting results of *MEF2A* protein expression in the E_349-inhibited SK-MEL-28-KRAB (**c**) and E_349-activated SK-MEL-28-VP64 (**d**) cells. **e,f,g** RT-qPCR results of *MEF2A* mRNA expression in the E_349-inhibited SK-MEL-2-KRAB (**e**) and -activated SK-MEL-2-VP64 (**f**), and E_349-KO A375 (**g**) cells. **h**,**i,j,k** CCK8 cell proliferation assay results of the E_349-inhibited A375-KRAB (**h**), E_349-inhibited SK-MEL-28-KRAB (**i**), and E_349-KO A375 (**j**) cells with 1 or 2 μM vemurafenib or without treatment at 24, 48, and 72 h, and E_349-inhibited SK-MEL-2 cells (**k**) (n = 4 samples). **l**,**m** Cell apoptosis (**l**) and Plate clone formation (**m**) assay results of the E_349-inhibited SK-MEL-28-KRAB cells with 1 μM vemurafenib or without treatment (n = 3 samples). **n**, Plate clone formation assay results of the E_349-inhibited SK-MEL-2-KRAB cells (n = 3 samples). CI denotes CRISPR interference, and CA denotes CRISPR activation. Left graph is the representative result, and right graph is the statistical result. All the data are expressed as the means ± SD and analyzed by an unpaired two-tailed Student's *t*-test. Asterisks indicate significant differences between the indicated experimental groups: *, $p < 0.05$; **, $p < 0.01$; ***, $p < 0.001$; ****, $p < 0.0001$.

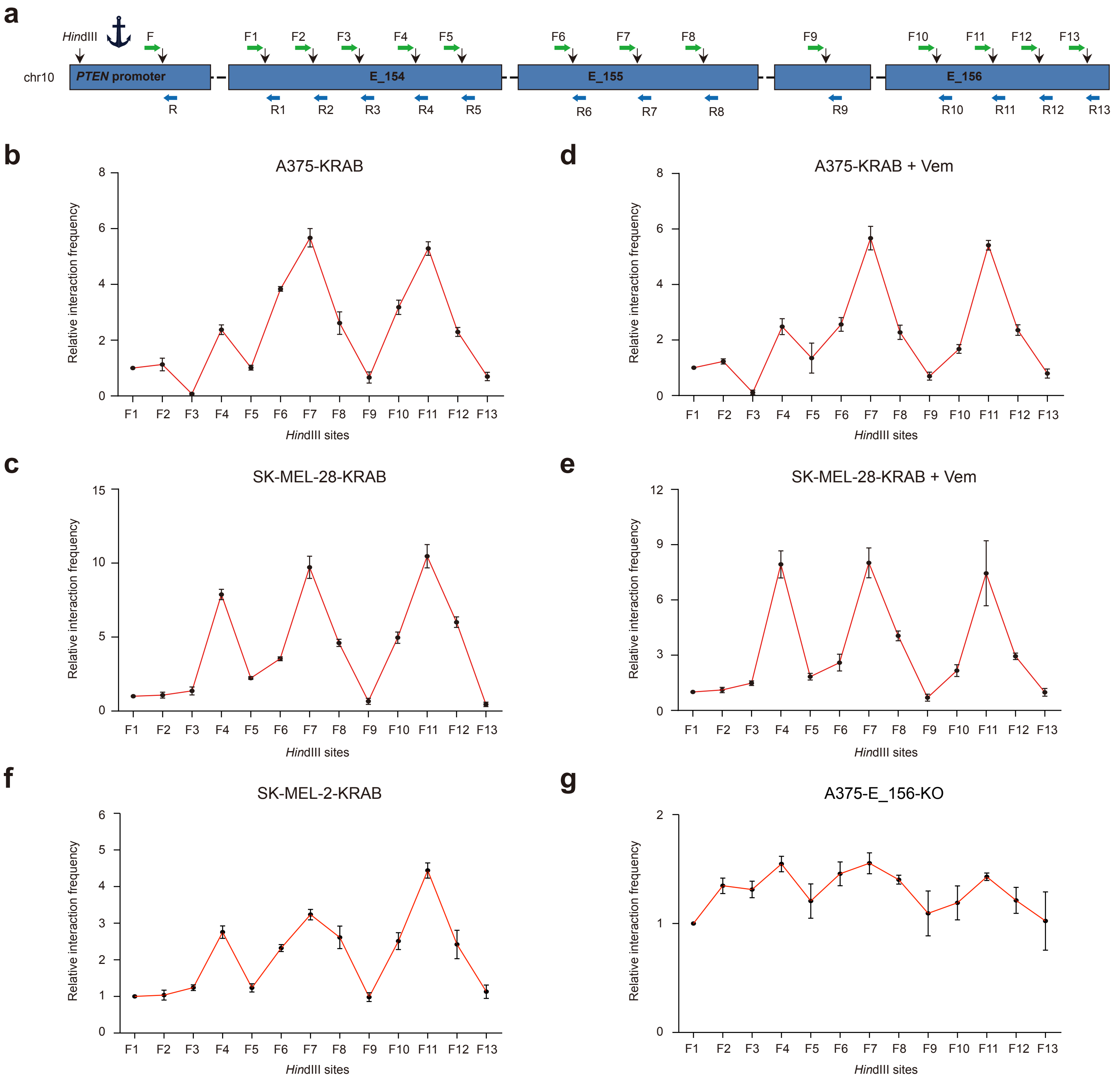
a
HindIII
F
F1 F2 F3 F4 F5
F6 F7 F8
F9
F10 F11 F12 F13
chr10
PTEN promoter
E_154
E_155
E_156
R
R1 R2 R3 R4 R5
R6 R7 R8
R9
R10 R11 R12 R13
b
A375-KRAB
d
A375-KRAB + Vem
c
SK-MEL-28-KRAB
e
SK-MEL-28-KRAB + Vem
f
SK-MEL-2-KRAB
g
A375-E_156-KO
Relative interaction frequency
HindIII sites
F1 F2 F3 F4 F5 F6 F7 F8 F9 F10 F11 F12 F13

Additional file1: Fig. S8 3C assay results between the *PTEN* promoter and E_156. a A schematic view of 3C assays at the chromosome region surrounding the *PTEN* promoter. The *Hin*dIII-cutting fragment containing the *PTEN* promoter (GRCh37/hg19 chr10: 89,620,814–89,627,996) is measured as a bait indicated by the anchor, and a series of fragments downstream of the *PTEN* promoter is measured as preys. Blue rectangles denote the chromosome, black arrows denote the *Hin*dIII-cutting sites, and green arrows denote the location and direction of primers used in 3C assay. Blue arrows denote the reverse primer used in amplifying the control fragments. **b**,**c** 3C assay results of interaction between the *PTEN* promoter and E_156 in A375-KRAB (**b**) and SK-MEL-28-KRAB (**c**) cells (n = 3 samples). **d**,**e** 3C assay results of interaction between the *PTEN* promoter and E_156 in A375-KRAB (**d**) and SK-MEL-28-KRAB (**e**) cells under treatment with 1 μM vemurafenib (n = 3 samples). **f**,**g** 3C assay results of interaction between the *PTEN* promoter and E_156 in SK-MEL-2-KRAB (**f**) and E_156-KO A375 (**g**) cells (n = 3 samples).

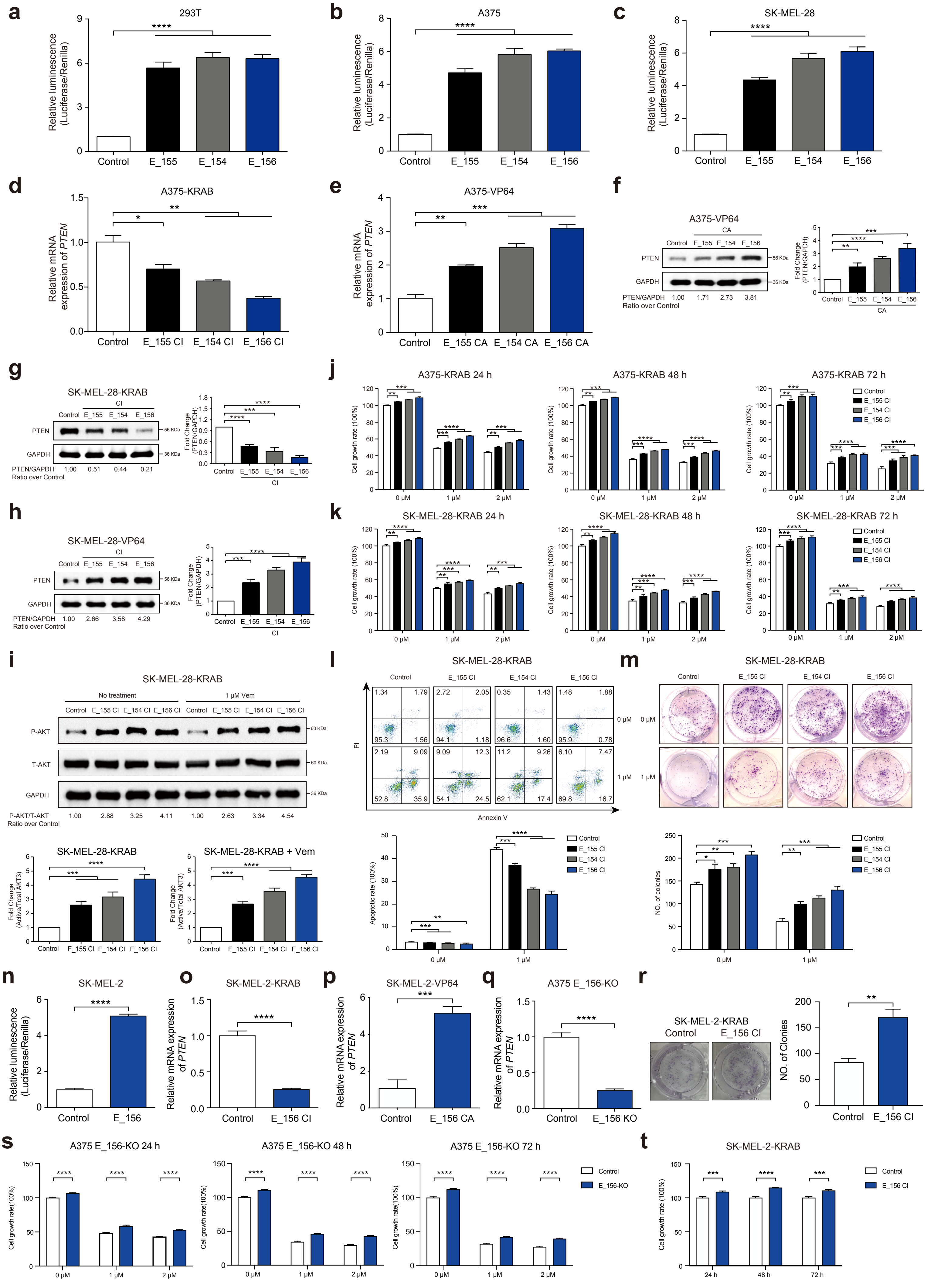

a
293T
Relative luminescence (Luciferase/Renilla)
Control
E_155
E_154
E_156
****
b
A375
c
SK-MEL-28
d
A375-KRAB
Relative mRNA expression of PTEN
E_155 CI
E_154 CI
E_156 CI
e
A375-VP64
E_155 CA
E_154 CA
E_156 CA
f
A375-VP64
CA
PTEN
GAPDH
56 KDa
36 KDa
PTEN/GAPDH Ratio over Control
1.00
1.71
2.73
3.81
Fold Change (PTEN/GAPDH)
g
SK-MEL-28-KRAB
CI
PTEN/GAPDH Ratio over Control
1.00
0.51
0.44
0.21
h
SK-MEL-28-VP64
1.00
2.66
3.58
4.29
i
SK-MEL-28-KRAB
No treatment
1 μM Vem
P-AKT
T-AKT
GAPDH
60 KDa
P-AKT/T-AKT Ratio over Control
1.00
2.88
3.25
4.11
1.00
2.63
3.34
4.54
SK-MEL-28-KRAB
SK-MEL-28-KRAB + Vem
Fold Change (Active/Total AKT3)
j
A375-KRAB 24 h
A375-KRAB 48 h
A375-KRAB 72 h
Cell growth rate (100%)
0 μM
1 μM
2 μM
k
SK-MEL-28-KRAB 24 h
SK-MEL-28-KRAB 48 h
SK-MEL-28-KRAB 72 h
l
SK-MEL-28-KRAB
PI
Annexin V
Apoptotic rate (100%)
m
SK-MEL-28-KRAB
NO. of colonies
n
SK-MEL-2
o
SK-MEL-2-KRAB
p
SK-MEL-2-VP64
q
A375 E_156-KO
E_156 KO
r
SK-MEL-2-KRAB
NO. of Clonies
s
A375 E_156-KO 24 h
A375 E_156-KO 48 h
A375 E_156-KO 72 h
E_156-KO
t
SK-MEL-2-KRAB
24 h
48 h
72 h

Additional file1: Fig. S9 Distal enhancer-sustaining *PTEN* tumor-suppressive potential in melanoma cells. a-c Luciferase assay results of the E_156 (E_155 or E_154 adjacent to E_156) core region in 293T cells (**a**), A375 cells (**b**), and SK-MEL-28 cells (**c**). **d**,**e** RT-qPCR results of *PTEN* mRNA expression in the E_156 (E_155 or E_154 adjacent to E_156)-inhibited A375-KRAB (**d**) and -activated A375-VP64 (**e**) cells. **f**-**h** Western blotting results of PTEN protein expression in the E_156 (E_155 or E_154 adjacent to E_156)-activated A375-VP64 (**f**), -inhibited SK-MEL-28-KRAB (**g**), and -activated SK-MEL-28-VP64 (**h**) cells. **i** Western blotting results of PI3K/AKT signaling pathway activation in the E_156 (E_155 or E_154 adjacent to E_156)-inhibited SK-MEL-28-KRAB cells with 1 μM vemurafenib or without treatment. P-AKT denotes phosphorylated AKT, and T-AKT denotes total AKT. **j**,**k** CCK8 cell proliferation assay results of the E_156 (E_155 or E_154 adjacent to E_156)-inhibited A375-KRAB (**j**) and -inhibited SK-MEL-28-KRAB (**k**) cells with 1 or 2 μM vemurafenib or without treatment at 24, 48, and 72 h (n = 4 samples). **l**,**m** Cell apoptosis (**l**) and Plate clone formation (**m**) assay results of the E_156 (E_155 or E_154 adjacent to E_156)-inhibited SK-MEL-28-KRAB cells with 1 μM vemurafenib or without treatment (n = 3 samples). **n**, Luciferase assay results of the E_156 core region in SK-MEL-2 cells. **o,p** RT-qPCR results of *PTEN* mRNA expression in the E_156-inhibited SK-MEL-2-KRAB (**o**) and -activated SK-MEL-2-VP64 (**p**) cells. **q** RT-qPCR results of *PTEN* mRNA expression in the E_156-KO A375 cells. **r** Plate clone formation assay results of the E_156-inhibited SK-MEL-2-KRAB cells (n = 3 samples). **s,t** CCK8 cell proliferation assay results of the E_156-KO A375 (**s**) and E156-inhibited SK-MEL-2-KRAB (**t**) cells (n = 4 samples). CI denotes CRISPR interference, and CA denotes CRISPR activation. Left or up graph is the representative result, and right or below graph is the statistical result. All the data are expressed as the means ± SD and analyzed by an unpaired two-tailed Student's *t*-test. Asterisks indicate significant differences between the indicated experimental groups: *, $p < 0.05$; **, $p < 0.01$; ***, $p < 0.001$; ****, $p < 0.0001$.

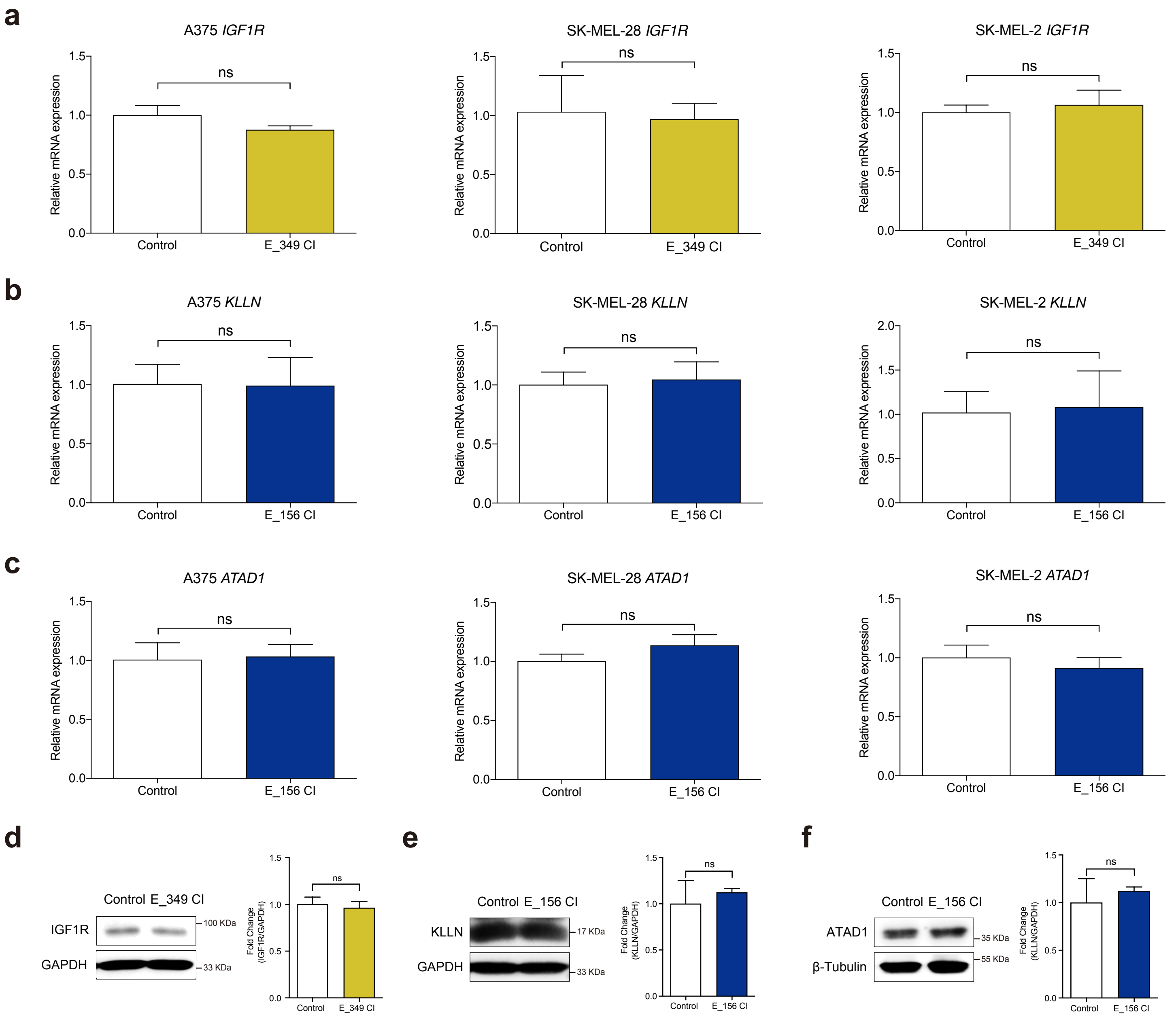
a
A375 IGF1R
SK-MEL-28 IGF1R
SK-MEL-2 IGF1R
Relative mRNA expression
ns
Control
E_349 CI
b
A375 KLLN
SK-MEL-28 KLLN
SK-MEL-2 KLLN
Relative mRNA expression
ns
Control
E_156 CI
c
A375 ATAD1
SK-MEL-28 ATAD1
SK-MEL-2 ATAD1
Relative mRNA expression
ns
Control
E_156 CI
d
Control E_349 CI
IGF1R
GAPDH
100 KDa
33 KDa
Fold Change (IGF1R/GAPDH)
ns
Control
E_349 CI
e
Control E_156 CI
KLLN
GAPDH
17 KDa
33 KDa
Fold Change (KLLN/GAPDH)
ns
Control
E_156 CI
f
Control E_156 CI
ATAD1
β-Tubulin
35 KDa
55 KDa
Fold Change (KLLN/GAPDH)
ns
Control
E_156 CI

Additional file1: Fig. S10 Detection the function of E_349 or E_156 on their predicted genes. **a**,**b**,**c** RT-qPCR results of *IGF1R* (**a**), *KLLN* (**b**), or *ATAD1* (**c**) mRNA expression in the E_349-inhibited or E_156-inhibited A375, SK-MEL-28 and SK-MEL-2 cells. **d**,**e**,**f** Western blotting results of IGF1R (**d**), KLLN (**e**), or ATAD1 (**f**) expression in the E_349-inhibited or E_156-inhibited A375 cells. CI denotes CRISPR interference. Left or up graph is the representative result, and right or below graph is the statistical result. All the data are expressed as the means ± SD (n = 3 samples) and analyzed by an unpaired two-tailed Student's *t*-test. "ns" represents no significant difference between the indicated experimental groups.

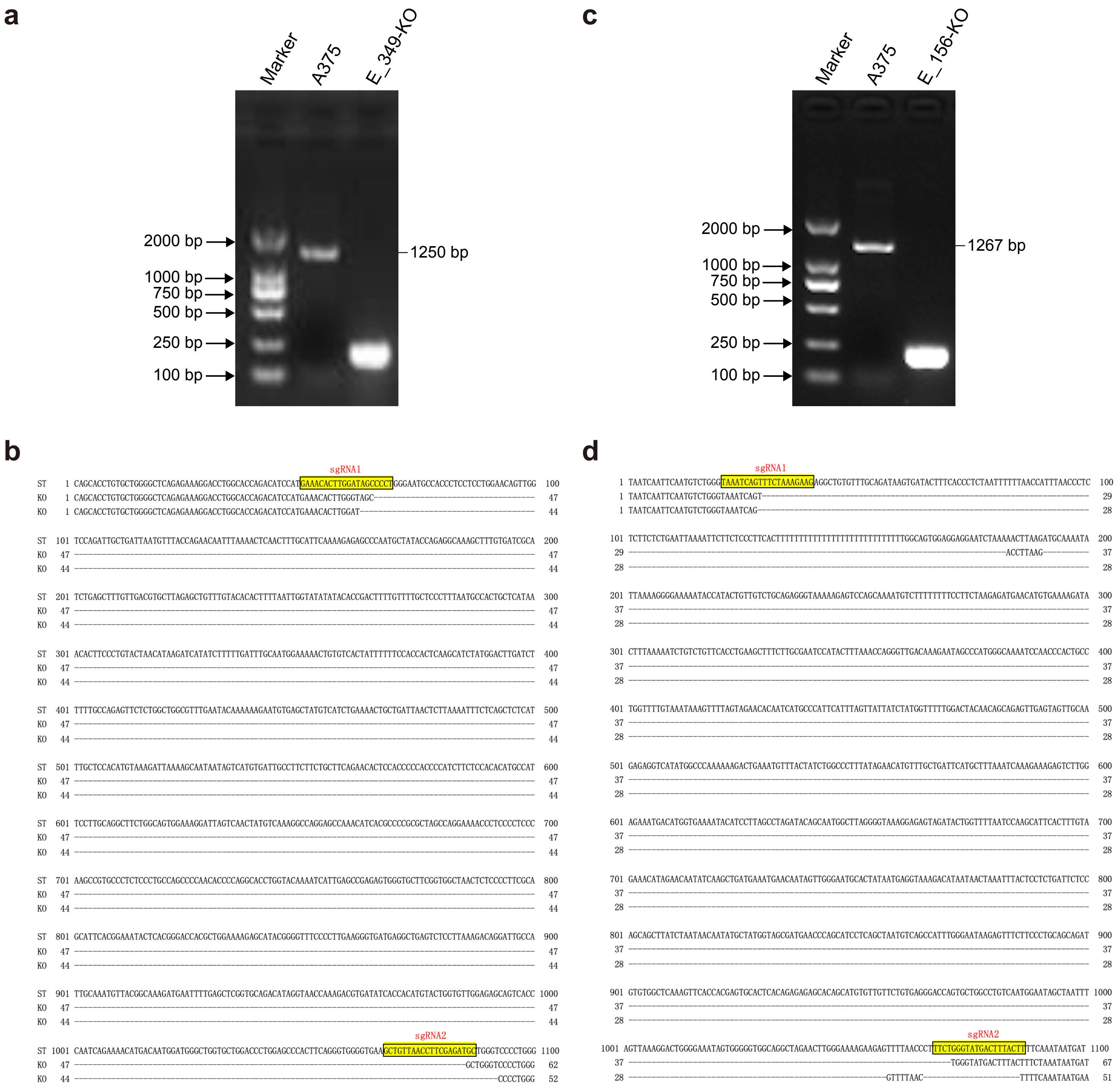
a
Marker
A375
E_349-KO
2000 bp
1000 bp
750 bp
500 bp
250 bp
100 bp
1250 bp
c
Marker
A375
E_156-KO
2000 bp
1000 bp
750 bp
500 bp
250 bp
100 bp
1267 bp
b
sgRNA1
sgRNA2
d
sgRNA1
sgRNA2

Additional file1: Fig. S11 Identification the knockout of E_349 or E_156 in A375 cells. **a**,**b** Electrophoresis results of E_349-knockout (KO) (**a**) and E_156-KO (**b**) in A375 cells. **c**,**d** Sanger sequencing alignment results of E_349-knockout (KO) (**c**) and E_156-KO (**d**) in A375 cells. The highlight regions represent the location of sgRNAs.

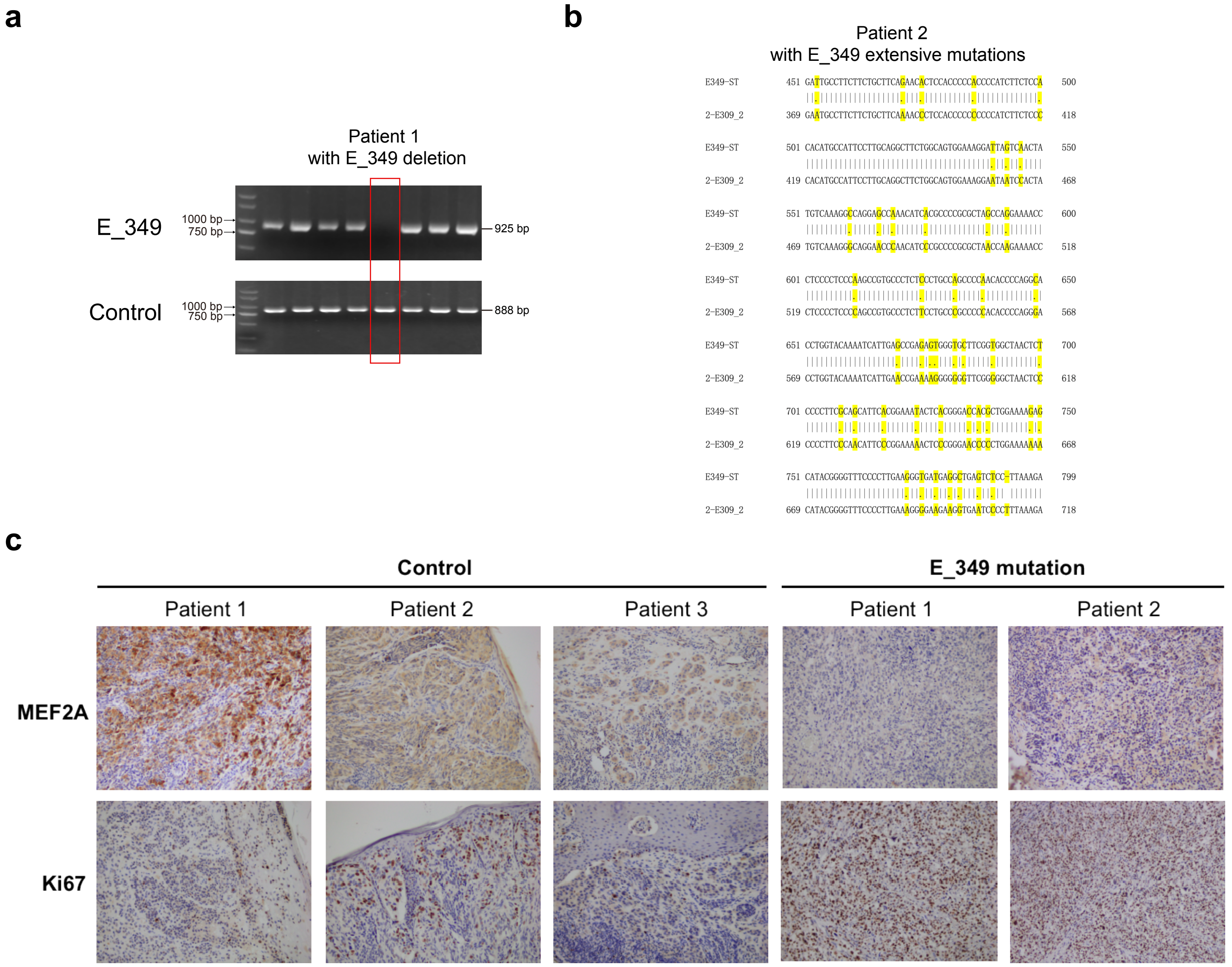
a
Patient 1
with E_349 deletion
E_349
1000 bp
750 bp
925 bp
Control
1000 bp
750 bp
888 bp
b
Patient 2
with E_349 extensive mutations
c
Control
E_349 mutation
Patient 1
Patient 2
Patient 3
Patient 1
Patient 2
MEF2A
Ki67

Additional file1: Fig. S12 Immunohistochemistry (IHC) results of melanoma patient samples with E_349 deletion or extensive mutations. We collected patient melanoma tissues, extracted the genomes, and amplified the DNA sequence harboring E349 core region for Sanger sequencing. One sample was detected with E_349 deletion (Patient 1) (**a**) and the other sample with extensive mutations (patient 2) (**b**). The samples with similar disease courses were used as random controls. These samples were performed IHC using MEF2A and Ki-67 antibodies (**c**).
