# Supplementary material for: Landscape of enhancer disruption and functional screen in melanoma cells
Source: Genome Biol. 2023 Oct 30;24:248. doi: 10.1186/s13059-023-03087-5 (PMC10614365; doi:10.1186/s13059-023-03087-5)
Supplement: Supplementary file 17 — Additional file 17. Review history. [file 13059_2023_3087_MOESM17_ESM.docx]

Review History

**First round of review**

**Reviewer 1**

**Are you able to assess all statistics in the manuscript, including the appropriateness of statistical tests used?** Yes, and I have assessed the statistics in my report.

**Comments to author:**

Luo and his/her colleagues combined 297 melanoma WGS results from public database and prioritized highly recurrent regions (HRRs) to functionalize. They overlapped this with H3K27ac-marked enhancers from A375 cells and identified a set of active HRR-associated enhancers on which they perform a genome-scale CRISPRi screen. With comprehensive bioinformatic analysis, the authors established an effective method for studying cancer highly recurrent regions and non-coding elements/enhancers. They focus deeper on E_349 and E_156 and suggest targeting of MEF2A and PTEN. The concept of functional enhancers that are genetically altered is quite appealing and can provide deeper insight into epigenetic mechanisms driving melanoma development/progression with future translational implications. However, the study fails to consider epigenetic/enhancer heterogeneity in melanoma, has some technical issues and required deeper validation.

* There is one major issue for the designing of CRISPRi screen in this work. The library was designed from a collection of human patient genome sequencing results. It represents the progressive mutation accumulation and diversity during melanoma development. However, the whole work only used a single melanoma cell line, A375, to perform the screen and tried to validate their method and hypothesis with this A375-derived screen result. Obviously, a single cancer cell line does not have the heterogenicity of human melanomas, hence the result using a full-spectrum library is hard to interpret. Authors should utilize existing H3K27ac and ATAC-Seq data from melanoma tumors (not cell lines) (there are several such datasets) to capture heterogeneity.

* Association of WGS derived HRR from melanoma patients to H3K27Ac and H3K4me1 peaks of melanoma cell lines is not backed by any experimental evidence which can establish connection between DEL-HRR and the loss of enhancer activity (which supposedly provides the proliferation advantage to melanoma cells). Direct evidence such as introduction of a DEL-HRR on an enhancer directly impacts enhancer function (or loss of H3k27ac) and subsequential loss of downstream target would be useful in establishing better rationale for the screen.

* In addition, the screen with an established cancer cell line is unlikely to reveal driver mutations that initiate melanoma development, and will suggest those that may be needed to sustain melanoma development/proliferation.

* Another flaw in the story is screen readout. Authors have chosen continuous division and proliferation of A375 cells as a function to analyze this screen as enrichment screen which creates a question of background amplification as the read out is same before and after the introduction of the library (i.e. not selection of a specific phenotype). This is one of the main reasons why drop-out screens are more popular.

* What were the gRNAs that dropped out in the screen? That is likely to identify interesting oncogenic events (although that is not supported by original selection of DEL-HRR regions as loss-of-function events).

* (Fig. 2b and method) The in vitro screen lasted for 21 days. That was exceptionally long for fast-proliferating cancer cells, which normally would be 7-10 days. The long screen time will cause severe background and the real hits might be shielded by false positive targets.

* Lack of rigor in data interpretation is an issue. (P7 line 7-11) The screen hit some HRR-associated enhancers in the vicinity of several melanoma oncogenes (RAF1, YAP1). The discovery is quite the opposite to the tumor suppressor enhancers the screen was originally designed to search for. This undermining result need to be seriously addressed.

* Another major issue lies at the analysis and validation for the CRISPRi screen result. The screen in A375 identified a panel of potential enhancers and 66 significant enhancers (hits) were assayed using epigenetic method in A375 cells (Fig. 3 and Fig. 4). Two significant ones, E_349 and E_156 were further analyzed in A375 and SK-MEL-28 (another BRAFV600E melanoma cell line) cells for their function as enhancers for adjacent tumor suppressive genes (Fig. 5 and Fig. 6). Detailed assays were designed and performed to support the author's discovery. However, some essential questions were ignored in the analysis.

o (Fig. 3 and Fig. 4) The designing of the screen was to identify HRR-associated enhancers that was suppressed in melanoma by high-frequency mutations. The first analysis should be the examination of any significant hits for their mutation status in A375 in comparison to normal melanocytes. Further validation could be done in other melanoma cell lines with different genetic mutations.

o (Fig. 5 and Fig. 6) The assays for two enhancers (E_349 and E_156) did support the conclusion that they were involved in tumor cell proliferation and the expression of the adjacent tumor suppressors. But those assays were not enough to show they were important for tumorigenesis and development. Authors need to perform in vivo experiments, if possible in immune proficient hosts given the known biology of melanoma, to test if these enhancers are really functional.

* In addition, the authors did not perform any analysis or experiment to suggest that the enhancers identified in melanoma cells were compromised, as compared with normal physiological condition, by somatic mutations. Histopathological staining of the patient samples where Del-HRR occurs to show that gene function and phenotype was impacted could be very helpful.

o

* More clarity is needed on how gene targets for some of the enhancers were identified. This is critical.

o Figure 1d) How were enhancers-promoter associations identified?

o Figure 2a) Using proximity of enhancer to nearest gene is not an acceptable metric for enhancer-promoter interactions. It is known that only ~7% of human enhancers are localized to their nearest gene. What are the results from running multiple random iterations based on random intergenic regions?

o Figure 2b, c) How were enhancer-promoter interactions identified. If proximity was used, this can be problematic for interpreting downstream results. In addition, how do the results on E-P interactions from A375 capitulate in other melanoma cell lines? Similar question on heterogeneity in these 3D looping patterns.

o Figure 3c) Similar to the above comments for Figure2, how were enhancer-promoter interactions identified. Also, does the finding in A375 cells capitulate in other melanoma samples?

o Figure 3e) Once again, it is a major problem using proximity to annotate enhancer elements. This is helped by Figure4 and the HiChIP analysis, however, this should have been used in Figure1 for initial enhancer-promoter interactions. In addition, HiChIP-seq should be performed in at least one more representative melanoma sample.

o Figure 4a) It is unclear if all the HRR enhancers contain a valid enhancer-promoter interaction. How many of the 66 HRR enhancers were interacting with the predicted gene?

o Figure 5a) Why was MEF2A not identified in the initial analysis. Is this due to the enhancer-promoter predictions used throughout the study? This is again concerning as E_349 was the top hit of the screen. In addition, why/how was MEF2A chosen to functionalize?

o Figure 6c) Does this enhancer also interact with either KLLN or ATAD1? It seems this enhancer is interacting with the 3' end of these genes in Figure 6a. This should also be evaluated due to the low reduction of PTEN protein expression upon enhancer loss if Figure 6f.

o In addition, how many valid interactions were identified in the HiChIP data? It is unclear from the methods.

o Figure 5) The results for MEF2A are interesting, however, it is extremely problematic that E_349 is the top enhancer and not associating with the predicted gene(s).

* Figure 1c) It is unclear how the mutation calls performed here compared to their original study (i.e., number of mutation calls, type of call, location of call, should be shown in supplemental information).

* Figure S4a-c) Is the SK-MEL-5 mutational landscape, including intergenic regions, the same as A375?

* Figure 3d) It is unclear how motifs were identified and what background used for analysis. Notably, it is surprising TF such as MITF were not identified? ChIP-seq for at least one TF should be performed to demonstrate direct overlap with the HRR enhancers.

* Figure 6f) The reduction of PTEN expression upon enhancer loss is not striking. Does this reach significance?

**Reviewer 2**

**Are you able to assess all statistics in the manuscript, including the appropriateness of statistical tests used?** No, I do not feel adequately qualified to assess the statistics.

**Comments to author:**

In this manuscript, Luo et al., first computationally identified highly recurrent regions (HRRs) in the non-coding genome of melanoma based on WGS data of a large of cohort of human melanoma samples. They further carried out a CRISPRi tiling screen of putative enhancers in these HRRs using cell growth of A375 melanoma cells as a readout. After identifying 66 enhancers with putative tumor suppressive roles from the screen, they used the H3K27ac HiChIP approach to identify target genes of these enhancers in A375 cells. They further examined the functions of two enhancers that targeting MEF2A and PTEN, respectively, in melanoma cell proliferation, apoptosis and drug resistance. Overall, this is a very interesting and comprehensive study. It has revealed important information on enhancer-mediated transcriptional dysregulation in melanoma carcinogenesis. I have only several minor comments.

1. It appears that enhancer annotation was based on H3K27Ac ChIP data from multiple melanoma cell lines, beyond A375 and SK-Mel-28 cells that most experiments were carried out in. How heterogenous were the data for various melanoma cell lines, in terms of enhancer calling? The authors may discuss this in the manuscript.

2. Fig. 4d: It is unclear what the percentage of target genes showed up as hits in the CRISPR screen is? The authors may want to tune down the conclusion if only a small percentage of them showed up in the CRISPR screen hit list.

3. In general, I suggest the authors need to be cautious in making conclusion regarding the role of target genes (derived from HiChIP data) in melanoma carcinogenesis, as they are not experimentally verified ( except for MEF2A and PTEN) and mostly just speculations at this stage. Chromatin interaction by itself does not dedicate functional relevance in the context of carcinogenesis.

4. Fig. 5h-J: IGF1R expression could be included as a control to confirm that IGF1R is not a target as the authors suggested.

5. Fig. 5j/k (and related supplementary figures): Since manipulation of E349 affected readouts under both control and vemurafenib treatment conditions, to examine the role of E349 in vemurafenib resistance, the authors should analyze the data in a different way, i.e. calculating the ratios between treatment versus control in different groups of cells. Same suggestion for similar figures in Fig. 6 regarding the PTEN enhancers.

**Authors Response**

**Point-by-point responses to the reviewers’ comments:**

**Reviewer #1:** Luo and his/her colleagues combined 297 melanoma WGS results from public database and prioritized highly recurrent regions (HRRs) to functionalize. They overlapped this with H3K27ac-marked enhancers from A375 cells ad identified a set of active HRR-associated enhancers on which they perform a genome-scale CRISPRi screen. With comprehensive bioinformatic analysis, the authors established an effective method for studying cancer highly recurrent regions and non-coding elements/enhancers. They focus deeper on E_349 and E_156 and suggest targeting of MEF2A and PTEN. The concept of functional enhancers that are genetically altered is quite appealing and can provide deeper insight into epigenetic mechanisms driving melanoma development/progression with future translational implications. However, the study fails to consider epigenetic/enhancer heterogeneity in melanoma, has some technical issues and required deeper validation.

***Response*:** We appreciate the reviewer's insightful comments regarding enhancer heterogeneity, the technical aspects of CRISPR screening, and the need for more comprehensive validation. In response to these concerns, we have conducted additional experiments to further investigate these areas. Please refer to the detailed response provided below for more information.

*       There is one major issue for the designing of CRISPRi screen in this work. The library was designed from a collection of human patient genome sequencing results. It represents the progressive mutation accumulation and diversity during melanoma development. However, the whole work only used a single melanoma cell line, A375, to perform the screen and tried to validate their method and hypothesis with this A375-derived screen result. Obviously, a single cancer cell line does not have the heterogenicity of human melanomas, hence the result using a full-spectrum library is hard to interpret. Authors should utilize existing H3K27ac and ATAC-Seq data from melanoma tumors (not cell lines) (there are several such datasets) to capture heterogeneity.

***Response*:** We are grateful for the constructive and valuable feedback. Indeed, as the reviewer astutely pointed out, our CRISPRi library was developed from a collection of human patient genome sequencing data, which represents the accumulation and diversity of mutations throughout melanoma development. A single cancer cell line, however, does not fully capture the heterogeneity inherent in human melanomas. To address this limitation, we have conducted further CRISPRi screening using SK-MEL-2 melanoma cells, which have a wild-type BRAF. Intriguingly, we found that 19 out of the 25 positive selected enhancers in SK-MEL-2 are also shared by the A375 cell line at either day 7 or day 21 of screening. Similarly, 13 out of the 29 negative selected enhancers (from drop-out analysis as suggested) are also shared by the A375 cell line at either of these time points. This suggests that some functional enhancers may exhibit context-specific activity in different melanoma cells. More details about these new screening results can be found in the responses below.

Importantly, in accordance with the reviewer's suggestion, we initially downloaded H3K27ac ChIP-seq data from the Gene Expression Omnibus (GEO) dataset GSE134043 [1, 2], which contains raw H3K27ac data from 17 melanoma patients. After calling narrow peaks and identifying signals captured in enhancer regions, we found that 58 out of the 66 significantly functional enhancers from the CRISPRi screening at A375 cells successfully match the H3K27ac signals of patient samples. This implies that the majority of these functional enhancers remain active in melanoma patients. We have incorporated these results into Supplementary Table 10 of our revised manuscript. Subsequently, we utilized ATAC-seq data from 13 melanoma patients to investigate whether the enhancers we screened reflect chromatin states in melanoma patients [3]. As a result, 18 out of the 66 enhancers contained the melanoma patient's ATAC-seq signal. This suggests that chromatin accessibility may exhibit greater heterogeneity across different melanoma contexts, and further underscores the need for a larger sample size to explore the intertumoral heterogeneity of HRR-associated enhancers. These results have been included in Supplementary Table 10 of our revised manuscript. Notably, the two enhancers, E_349 and E_156, which we strongly highlighted and experimentally validated, both carry the melanoma patient's ATAC-seq signal (**Fig. 1 below**). This finding further underscores the significance of E_349 and E_156 in melanoma.

In addition, based on the above analysis, we must acknowledge that our CRISPR library in this study does have certain limitations in capturing the heterogeneity of HRR-associated enhancers. We believe that by summarizing the epigenetic markers from a larger cohort of melanoma patients, we could more systematically and comprehensively capture these potentially crucial regulatory elements. This would enable us to design improved libraries for functional screening and identification in a broader range of melanoma cell models, including cutaneous, acral, and mucosal types. We have briefly discussed this aspect (**L487-494**) in our revised manuscript.

**Fig. 1 | IGV [4] screenshot displaying ATAC-seq signals in E_349 and E_156 from melanoma patients.** The H3K27ac (n=17) and ATAC-seq (n = 13) signals from patients were loaded into the IGV track to present the chromatin activities in the regions proximal to E_349 and E_156. The results demonstrate that both E_349 and E_156 possess ATAC-seq signals from melanoma patients.

*       Association of WGS derived HRR from melanoma patients to H3K27Ac and H3K4me1 peaks of melanoma cell lines is not backed by any experimental evidence which can establish connection between DEL-HRR and the loss of enhancer activity (which supposedly provides the proliferation advantage to melanoma cells). Direct evidence such as introduction of a DEL-HRR on an enhancer directly impacts enhancer function (or loss of H3k27ac) and subsequential loss of downstream target would be useful in establishing better rationale for the screen.

***Response*:** Thanks for the constructive comments. In order to further detect the function of E_349 and E_156 in melanoma development, we knocked out the core region of these two enhancers in A375 cells by CRISPR technology and constructed the A375 E_349-KO (**Fig. 1a, b below**) and A375 E_156-KO (**Fig. 2a, b below**) melanoma cells, respectively. Firstly, we detected the expression of *MEF2A* and *PTEN* in these two melanoma cells. RT-qPCR result indicated that when E_349 or E_156 was knocked out, the expression of *MEF2A* (**Fig. 1c below**) or *PTEN* (**Fig. 2c below**) was significantly decreased, highlighting the important function of E_349 or E_156 in modulating *MEF2A* or *PTEN* expression. Next, to access the function of E_349 and E_156 on the melanoma phenotypes, we performed cell proliferation experiment. Consequently, the proliferative abilities of the cells in the E_349 or E_156-inhibited group was markedly higher than those in the control group (all p-value < 0.0001) (**Fig. 1d and Fig. 2d below**). We then performed 3C assays on these two enhancer-KO A375 cells. As a result, compared with the A375-KRAB melanoma (control) cells (Fig. S5b and Fig. S8b in the revised manuscript), the interaction between the *MEF2A* promoter and the region (F15 fragment in Fig. S5a of the revised manuscript) harboring E_349 in A375 E_349-KO melanoma cells was significantly weakened (**Fig. 3 below**), and the interaction between the *PTEN* promoter and the region (F11 fragment in Fig. S8a of the revised manuscript) harboring E_156 in A375 E_156-KO melanoma cells was also significantly weakened (**Fig. 4 below**), highlighting the important role of E_349 and E_156 in the sustaining the chromatin interactions at these DEL-HRR loci. In summary, introducing deletion events on E_349 or E_156 directly affects their enhancer function, leading to the subsequent loss of their downstream targets. **We have added the results into Fig. S11, Fig. S7g, j, Fig. S9q, s, Fig. S5g, Fig. S8g, the related description (L370-372, L440-442, L372-373, L442-L443 L373-376, and L407), and figure legend (L1523-1526, L1457-1462, L1503-1506, L1446-1447 and L1483-1484) in the revised manuscript, and please check our revised manuscript.**

**Fig. 1 | E_349 knockout contributes to the proliferation of A375 melanoma cells by downregulating *MEF2A*. a** Electrophoresis result of identifying the E_349 knockout (KO) in A375 melanoma cells. **b** Sanger sequencing alignment result for E_349 knockout in E_349-KO A375 and control cells. **c** RT-qPCR result of *MEF2A* expression in the E_349-KO A375 cells (n = 3 samples). **d** CCK8 cell proliferation assay results of the E_349-KO cells with 1 or 2 µM vemurafenib or without treatment at 24, 48, and 72 h (n = 4 samples). KO denotes CRISPR knockout. All the data are expressed as the means ± standard deviations (SD) and analyzed by an unpaired two-tailed Student’s *t*-test: *, p < 0.05, **, p < 0.01, ***, p < 0.001, ****, p < 0.0001.

**Fig. 2 | E_156 knockout contributes to the proliferation of A375 melanoma cell by downregulating *PTEN*. a** Electrophoresis result of identifying the E_156 knockout (KO) in A375 melanoma cells. **b** Sanger sequencing alignment result for E_156 knockout in E_156-KO A375 and control cells. **c** RT-qPCR result of *MEF2A* expression in the E_156-KO A375 cells (n = 3 samples). **d** CCK8 cell proliferation assay results of the E_156-KO cells with 1 or 2 µM vemurafenib or without treatment at 24, 48, and 72 h (n = 4 samples). KO denotes CRISPR knockout. All the data are expressed as the means ± standard deviations (SD) and analyzed by an unpaired two-tailed Student’s *t*-test: *, p < 0.05, **, p < 0.01, ***, p < 0.001, ****, p < 0.0001.

**Fig. 3 | 3C results of between the *MEF2A* promoter and E_349 in E_349-KO A375 cells. a** Electrophoresis results of 3C assay for the *MEF2A* promoter in A375 E_349-KO cells. **b** Relative interaction frequencies between the *MEF2A* promoter and representative *Hin*dIII-cutting fragments indicated in Fig. S5 in the revised manuscript. Data are represented as the means ± standard deviations (SD).

**Fig. 4 | 3C results of between the *PTEN* promoter and E_156 in E_156-KO A375 cells. a** Electrophoresis results of 3C assay for the *PTEN* promoter in A375 E_156-KO cells. **b** Relative interaction frequencies between the *PTEN* promoter and representative *Hin*dIII-cutting fragments indicated in Fig. S8 in the revised manuscript. Data are represented as the means ± standard deviations (SD).

*       In addition, the screen with an established cancer cell line is unlikely to reveal driver mutations that initiate melanoma development, and will suggest those that may be needed to sustain melanoma development/proliferation.

***Response*:** We appreciate the constructive feedback. As the reviewer pointed out, a screen with an established cancer cell line is unlikely to reveal driver mutations that initiate melanoma development. We fully agree with this perspective. However, conducting functional screening in a cell model that simulates tumor initiation can be challenging, and the results we obtained mainly reflect contributions to sustaining melanoma development or proliferation. We have toned down and rephrased related statements in our revised manuscript. Additionally, we have used another melanoma cell line, SK-MEL-2 (which does not harbor a BRAF mutation), to perform the CRISPRi screen (as mentioned below) and carry out the related phenotypic experiments.

*       Another flaw in the story is screen readout. Authors have chosen continuous division and proliferation of A375 cells as a function to analyze this screen as enrichment screen which creates a question of background amplification as the read out is same before and after the introduction of the library (i.e. not selection of a specific phenotype). This is one of the main reasons why drop-out screens are more popular.

***Response*:** We appreciate the insightful and professional question. Yes, as the reviewer pointed out, background amplification is indeed a critical factor that influences the readout in positive selection screens. In this study, we primarily focused on the positive selection screen to explore the role of HRR-associated enhancer destruction in melanoma onset and progression. Previously, by designing strategies to remove guides with the lowest count in each sample, using non-targeting control guides to estimate the size factor for normalization, and estimating significant enrichment at the enhancer level with tiling sgRNAs, we have attempted to control for background amplification as much as possible. However, we must acknowledge that some potential uncontrolled background amplification could still exist. In response to the reviewer's suggestion, we have conducted the drop-out analysis and added a day7 screening on both A375 (BRAF^V600E^) and another newly included melanoma cell line, SK-MEL-2 (BRAF wild-type), as mentioned below.

In the revised manuscript, we performed CRISPRi both positive and negative selection screens on A375 and SK-MEL-2 melanoma cells, respectively, by culturing them for 7 days (day7 screen). We applied the MAGeCK-RRA algorithm [5] to calculate enrichment, comparing the day7 group against the day0 group, with each group containing two replicates. Consequently, for A375 cells, 196 enhancers were identified in the positive screen and 87 enhancers in the negative screen (p-value < 0.05) (**Table 1 as below**). We then compared the results of the day7 screen with those of the day21 screen in A375 as described in the manuscript. As a result, we found that 14 of the 66 hits in the day21 screen were also enriched in the day7 screen, including E_349. In the negative screen, even though only two enhancers showed consistency in both day7 and day21 screens (the primary reason could be that drop-out screening often loses power during prolonged culture, especially in the case of fast-proliferating cancer cells), we observed that E_763, an enhancer we briefly describe below, was also a negative hit in the day7 screen. According to HiChIP loop data, this enhancer targets the classic melanoma driver gene, *MITF*. These results have been included in **Fig. S3a, b** of our revised manuscript

In addition, in the day7 screen of SK-MEL-2 melanoma cells, we identified 25 enhancers in the positive screen and 29 in the negative screen (p-value < 0.05) (**Table 1 as below**). We examined the consistency of the results among the SK-MEL-2 day7 screen, A375 day7 screen, and A375 day21 screen. Notably, 19 of the 25 positive enhancers in SK-MEL-2 are also shared by either the A375 day7 or day21 screens, and 13 of the 29 negative enhancers are also shared by either the A375 day7 or day21 screens. Importantly, E_156, the enhancer on which we focused, also showed enrichment in SK-MEL-2 melanoma cells. These results have been included in **Fig. S3c, d** of our revised manuscript. Taken together, these results suggest that by conducting CRISPRi screens at different time points on two genetically heterogeneous melanoma cell lines, A375 and SK-MEL-2, we were able to discover that 1) the analysis of day7 screening could reveal more functional enhancers, especially for drop-out screening; 2) over half of the hits from SK-MEL-2 have a detectable effect in both A375 and SK-MEL-2 cells, suggesting that the enhancers we designed by locating HRR are likely to function in more than one or two cell lines, possibly more comprehensively; 3) both E_156 and E_349 are hits in both the day7 and day21 screens, indicating these two enhancers might have more stable effects on melanoma survival, deserving further in-depth validation.

Lastly, we must acknowledge that background amplification and the selection of screening timeframes can be a double-edged sword. The presence of certain background amplification can obscure enhancers with less impact on phenotypes, thereby helping to identify more functionally significant enhancers. In future studies, we will focus on both drop-out and positive selection screens, combining the two methods for analysis, which will help us to explore scientific questions more comprehensively. We are very pleased to engage in discussion with the reviewer on professional issues related to CRISPR screening, and we have briefly discussed such caveats (**L509-518**) in the revised manuscript.

Table 1. Significant enhancers in day7 screening.

|  | **ID** | **enhancer core location** | **HRR type** | **Target gene** | **Screen out in A375 (day7)** | **Screen out in SK-MEL-2 (day7)** |
| --- | --- | --- | --- | --- | --- | --- |
| **Positive screen** | E_113 | 1:229080229-229080401 | TRA | *TMEM78,RHOU* | Y |  |
|  | E_150 | 10:89482835-89483866 | DEL | *PAPSS2* | Y |  |
|  | E_200 | 11:67499694-67500146 | DUP |  | Y |  |
|  | E_296 | 13:114523655-114524802 | DEL | *RASA3,GAS6,TMEM255B* | Y |  |
|  | E_349 | 15:99982353-99983277 | DEL | *LRRC28,MEF2A* | Y |  |
|  | E_367 | 17:1325981-1326684 | DEL | *YWHAE,CRK,MYO1C* | Y |  |
|  | E_387 | 17:16176649-16177825 | DEL | *TRPV2,CENPV,PIGL,NCOR1* | Y |  |
|  | E_397 | 17:27466969-27467964 | SNV | *CRYBA1,NUFIP2,PIPOX,PHF12,SEZ6* | Y |  |
|  | E_512 | 19:11877420-11878490 | DEL | *ZNF441,ZNF440,ZNF491,ZNF823* | Y |  |
|  | E_653 | 21:43104479-43105245 | DEL |  | Y |  |
|  | E_740 | 3:12525390-12526456 | DEL,DUP | *PPARG,SYN2,TIMP4* | Y |  |
|  | E_747 | 3:48288859-48289457 | DUP | *SHISA5,TREX1,ATRIP,TMA7,CDC25A,CCDC51,PLXNB1* | Y |  |
|  | E_761 | 3:69413277-69413949 | DEL | *FRMD4B,ARL6IP5,UBA3* | Y |  |
|  | E_854 | 7:56077329-56077993 | DEL,DUP | *ZNF713,SUMF2,PSPH,MRPS17,CCT6A* | Y |  |
|  | E_156 | 10:89986509-89987396 | DEL | *ATAD1,KLLN,PTEN* |  | Y |
|  | E_16 | 1:11039449-11040555 | DEL | *DFFA,PEX14,TARDBP,MASP2* |  | Y |
|  | E_546 | 19:18385275-18386754 | DEL | *UBA52,PGLS,MAST3,PIK3R2,IFI30,MPV17L2,RAB3A,PDE4C,LSM4,PGPEP1,GDF15,LRRC25,SSBP4,ISYNA1,ELL,SLC27A1,KXD1* |  | Y |
|  | E_852 | 7:55766472-55767180 | DUP |  |  | Y |
| **Negative screen** | E_763 | 3:69836247-69837207 | DUP | *MITF* | Y |  |
|  | E_530 | 19:13957341-13958809 | DUP | *JUNB,HOOK2,DDX39A,PKN1,DNAJB1,TECR,PODNL1,DCAF15,RFX1,IL27RA,PALM3,C19orf67,SAMD1,*  *PRKACA,GIPC1,NDUFB7,CLEC17A,CC2D1A,RLN3,*  *NANOS3,ASF1B,C19orf53,PRDX2,RNASEH2A,*  *RTBDN,MAST1,BEST2,GCDH,ZSWIM4,FARSA,CALR,RAD23A,SYCE2,DAND5,GADD45GIP1,CACNA1A,*  *IER2,STX10,MRI1,TRMT1,LYL1,NFIX,NACC1,KLF1* | Y |  |
|  | E_637 | 20:35112090-35112733 | DEL |  |  | Y |

*       What were the gRNAs that dropped out in the screen? That is likely to identify interesting oncogenic events (although that is not supported by original selection of DEL-HRR regions as loss-of-function events).

***Response*:** Thanks for the constructive comments. According to the reviewer’s suggestion, we analyzed the sgRNAs that dropped out in the screen. Consequently, we used the MAGeCK-RRA algorithm [5] to screen out 16 negatively selected enhancers with FDR < 0.05, of which nearly half belonged to the DUP type (**Table 1 and Fig. 1 below**). Among their target genes, the presence of canonical oncogene *MITP* may arise a gain-of-function hypothesis that duplication on E_763 may regulate the expression of *MITP* and drives tumor progression.

**Table 1. Results of CRISPRi negative selection screen (day21 versus day0).**

| **id** | **enhancer location** | **neg\|fdr** | **neg\|lfc** | **variant** | **target gene** | **Oncogene (CancerMine)** |
| --- | --- | --- | --- | --- | --- | --- |
| E_394 | 17:16918009-16918495 | 0.001068 | -1.2088 | DEL | *FLCN*, COPS3, *MPRIP* | *FLCN* |
| E_497 | 19:5985341-5985792 | 0.001068 | -0.73503 | DUP | *CAPS*, *CD70*, *RANBP3* |  |
| E_804 | 5:1792880-1793417 | 0.001068 | -1.139 | DEL |  |  |
| E_376 | 17:6655806-6656127 | 0.001068 | -2.0114 | DUP | *SLC16A13*, *BCL6B*, *C17orf49*, *RNASEK*, *ALOX12* |  |
| E_545 | 19:17976074-17977267 | 0.023504 | -0.87705 | SNV | *IFI30*, *PIK3R2*, *MAST3*, *ARRDC2*, *KCNN1*, *CCDC124*, *B3GNT3*, *FCHO1*, *MAP1S*, *UNC13A*, *PGLS*, *SLC27A1*, *NXNL1*, *COLGALT1*, *BST2*, *GTPBP3*, *ANO8*, *MVB12A* | *B3GNT3*, *BST2* |
| **E_763** | **3:69836247-69837207** | **0.023504** | **-0.70371** | **DUP** | ***MITF*** | ***MITF*** |
| E_379 | 17:7677267-7678089 | 0.027473 | -0.30255 | DEL | *DNAH2*, *KDM6B*, *TMEM88* | *KDM6B* |
| E_351 | 15:100026314-100027579 | 0.041667 | -0.86045 | DEL |  |  |
| E_677 | 22:21137931-21138863 | 0.041667 | -0.59227 | DUP | *SERPIND1*, *PI4KA* |  |
| E_318 | 15:39437940-39438530 | 0.041667 | -0.47847 | SNV | *THBS1*, *FSIP1* | *FSIP1* |
| E_530 | 19:13947666-13948172 | 0.041667 | -0.30886 | DUP | *JUNB*, *HOOK2*, *DDX39A*, *PKN1*, *DNAJB1*, *TECR*, *PODNL1*, *DCAF15*, *RFX1*, *IL27RA*, *PALM3*, *C19orf67*, *SAMD1*, *PRKACA*, *GIPC1*, *NDUFB7*, *CLEC17A*, *CC2D1A*, *RLN3*, *NANOS3*, *ASF1B*, *C19orf53*, *PRDX2*, *RNASEH2A*, *RTBDN*, *MAST1*, *BEST2*, *GCDH*, *ZSWIM4*, *FARSA*, *CALR*, *RAD23A*, *SYCE2*, *DAND5*, *GADD45GIP1*, *CACNA1A*, *IER2*, *STX10*, *MRI1*, *TRMT1*, *LYL1*, *NFIX*, *NACC1*, *KLF1* | *JUNB*, *PKN1*, *DNAJB1*, *PRKACA*, *ASF1B*, *PRDX2*, *CALR*, *LYL1*, *NACC1* |
| E_50 | 1:33182249-33182827 | 0.041667 | -0.1471 | DUP |  |  |
| E_684 | 22:29168330-29169614 | 0.047009 | -0.79465 | DEL | *XBP1*, *HSCB*, *CHEK2* | *XBP1*, *CHEK2* |
| E_871 | 7:140325981-140326709 | 0.047009 | -0.27918 | DUP, TRA |  |  |
| E_75 | 1:156163289-156164433 | 0.047009 | -0.14247 | DUP | *SEMA4A*, *LMNA*, *PMF1* | *LMNA* |
| E_637 | 20:35112090-35112733 | 0.047009 | -0.7174 | DEL |  |  |

**Fig 1 | The distribution of functional enhancers varies across different types of HRR in drop-out screen.** DUP-HRRs, in particular, exhibit stronger enrichment in CRISPRi negative selection screens.

*       (Fig. 2b and method) The in vitro screen lasted for 21 days. That was exceptionally long for fast-proliferating cancer cells, which normally would be 7-10 days. The long screen time will cause severe background and the real hits might be shielded by false positive targets.
***Response*:** Thank you for the insightful comments. In response to the reviewer's suggestions, we have added results of the CRISPRi screening at 7 days for both A375 (BRAF^V600E^) and SK-MEL-2 (BRAF wild-type) cells, as mentioned earlier. Briefly, by comparing the results from day 7 and day 21 screenings, we found that day 7 screening revealed a greater number of functional enhancers, especially in drop-out tests. More than half of the hits from SK-MEL-2 had an observable impact on A375 cells, underscoring the broad applicability of many functional HRR-associated enhancers. Importantly, enhancers E_156 and E_349 consistently affected melanoma survival across multiple screenings, and therefore deserve further in-depth study. However, despite our best efforts to minimize background amplification through methods such as guide removal optimization, normalization using non-targeting controls, and enhancer-level enrichment estimation, we acknowledge the potential existence of uncontrolled background amplification during the screens. We also briefly discussed such problem (**L509-518**) in the revised manuscript.

*       Lack of rigor in data interpretation is an issue. (P7 line 7-11) The screen hit some HRR-associated enhancers in the vicinity of several melanoma oncogenes (RAF1, YAP1). The discovery is quite the opposite to the tumor suppressor enhancers the screen was originally designed to search for. This undermining result need to be seriously addressed.

***Response*:** Thanks for the constructive and challenging comments. Indeed, *YAP1* [6] and *RAF1* [7] are key candidate genes associated with melanoma cell growth and drug resistance, as identified in published CRISPR/shRNA screening studies. As such, they were highlighted in our previous Fig. 2. These genes have been reported to be linked to melanoma development and are located proximally to the HRR-associated enhancers (~1Mb) in the genome. However, HiChIP results suggest that they may not be the actual target genes in the A375 melanoma cell line (see Supplementary Table 12). We identified that the screened enhancer E_223 targets not only *YAP1* but also *BIRC2*. Using TCGA-SKCM clinical data, we found that *BIRC2* expression correlates with a good prognosis **(Fig 1, below)**, suggesting that E_223 could play a tumor-suppressing role by targeting *BIRC2* and not *YAP1*. Similarly, *RAF1* does not appear to be a real target gene, with no HiChIP loop supporting its connection with a screened enhancer. Intriguingly, our most recent study [8] discovered that CRISPRi can indeed activate certain target gene in CROP-seq screens. In this context, CREs can be hits in both CRISPRi and CRISPRa screenings (as shown in "Endogenous fine-mapping of functional regulatory elements in complex genetic loci | bioRxiv, Fig. 6"), though the specific mechanism requires further exploration. We have briefly discussed the phenomenon (**L535-539**) in the revised manuscript.

**Fig. 1 | The overall survival analysis of *BIRC2* and *YAP1* in TCGA-SKCM.** Kaplan-Meier cure of gene *BIRC2* and *YAP1*, respectively. Survival analysis was based on gene expression in TCGA-SKCM using GEPIA2 [9].

*       Another major issue lies at the analysis and validation for the CRISPRi screen result. The screen in A375 identified a panel of potential enhancers and 66 significant enhancers (hits) were assayed using epigenetic method in A375 cells (Fig. 3 and Fig. 4). Two significant ones, E_349 and E_156 were further analyzed in A375 and SK-MEL-28 (another BRAFV600E melanoma cell line) cells for their function as enhancers for adjacent tumor suppressive genes (Fig. 5 and Fig. 6). Detailed assays were designed and performed to support the author's discovery. However, some essential questions were ignored in the analysis.

***Response*:** Thank you for raising your concerns. In the revised manuscript, we have substantially bolstered our argument for the functionality of these two enhancers by including a wealth of evidence from cellular, animal, and patient sample studies.

o       (Fig. 3 and Fig. 4) The designing of the screen was to identify HRR-associated enhancers that was suppressed in melanoma by high-frequency mutations. The first analysis should be the examination of any significant hits for their mutation status in A375 in comparison to normal melanocytes. Further validation could be done in other melanoma cell lines with different genetic mutations.

***Response*:** Thank you for the constructive comments. Following the reviewer's suggestion, we analyzed the mutation status of significant hits in A375 melanoma cells, comparing them with other melanocyte/melanoma cell lines at diverse carcinogenic stages from the Cancer Cell Line Encyclopedia (CCLE) [10]. Among all 71 melanocyte/melanoma cell lines in the CCLE, including A375, we initially assessed the presence of somatic copy number variation (Gain, heterozygous loss) and somatic structural variations (DEL, DUP, INV, TRA) within the enhancer genome region. The results showed that 21 out of 66 enhancers, including E_349 and E_156, exhibited gain events, while 17 out of 66 had heterozygous loss events in the A375 melanoma cell line. When considering all the cell lines, 30 cell lines displayed heterozygous loss, one cell line (hs944t) had a deletion, and only A375 showed a gain in the E_156 region. In the E_349 region, 22 cell lines showed a gain, while 6 cell lines exhibited heterozygous loss. Due to the design of CRISPRi, these enhanced deletion events are generally not seen in the A375 cell line but may be present in other cell line genomes. The tumor environment of a cell line may also contribute to enhancer dysregulation. We have included more detailed information about the genetic features of melanoma cells into Supplementary Table 11.

Next, we used another melanoma cell line SK-MEL-2 (BRAF wild-type) to further validate our conclusions. The luciferase assay result showed that either the core region of E_349 or E_156 significantly promote the luciferase activities, indicating both E_349 and E_156 have the enhancer activities in SK-MEL-2 melanoma cells (**Fig. 1 below**). Then, we constructed the SK-MEL-2-KRAB cells and SK-MEL-2-VP64 by CRISPR technology as described in the method section of the manuscript. The 3C results indicated that the region including *MEF2A* promoter has a stronger interaction with F5 (the region harboring E_349, see Fig. S5a in the revised manuscript) (**Fig. 2 below**); and the region including *PTEN* promoter has a stronger interaction with F11 (the region harboring E_156, see Fig. S8a in the revised manuscript) (**Fig. 3 below**). Next, we detected the expression of *MEF2A* and *PTEN*, and their corresponding phenotypes. As a result, we acquired the similar phenotypes in SK-MEL-2 melanoma cells with the A375 and SK-MEL-28 melanoma cells (**Fig. 4 and 5 below**). Taken together, our data indicate that both E_349 and E_156 can regulate the expression of their target genes affecting melanoma development in SK-MEL-2 melanoma cells. Please also check our revised manuscript. **We have added the results into Fig. S7b, e, f, k, n, Fig. S9n, o, p, r, t, Fig. S5f, Fig. S8f, the related description (L346-356, L418-443, L335, and L407), and figure legend (L1450-1451, L1487-1488, L1458-1469, and L1502-1508) in the revised manuscript, and please check our revised manuscript.**

**Fig. 1 | Luciferase activities of E_349 and E_156 in SK-MEL-2 melanoma cells. a,b** The core sequences of E_349 and E_156 were cloned into pGL3-Promoter vector for enhancer assay, respectively. The recombinant plasmids were transfected into SK-MEL-2 cells, and the luciferase activities of E_349 (**a**) and E_156 (**b**) were detected. All the data are expressed as the means ± standard deviations (SD) and analyzed by an unpaired two-tailed Student’s *t*-test: *, p < 0.05, **, p < 0.01, ***, p < 0.001, ****, p < 0.0001.

**Fig. 2 | 3C results of between the *MEF2A* promoter and E_349 in SK-MEL-2-KRAB cells. a** Electrophoresis results of 3C assay for the *MEF2A* promoter in SK-MEL-2-KRAB cells. **b** Relative interaction frequencies between the *MEF2A* promoter and representative *Hin*dIII-cutting fragments indicated in Fig. S5 in the revised manuscript. Data are represented as the means ± standard deviations (SD).

**Fig. 3 | 3C results of between the *PTEN* promoter and E_156 in SK-MEL-2-KRAB cells. a** Electrophoresis results of 3C assay for the *PTEN* promoter in SK-MEL-2-KRAB cells. **b** Relative interaction frequencies between the *PTEN* promoter and representative *Hin*dIII-cutting fragments indicated in Fig. S8 in the revised manuscript. Data are represented as the means ± standard deviations (SD).

**Fig. 4 | Inhibition of E_349 contributes to the proliferation of SK-MEL-2 melanoma cells by downregulating *MEF2A*. a** RT-qPCR result for *MEF2A* in the E_349-inhibited SK-MEL-2-KRAB cells (n = 3 samples). **b** RT-qPCR result for *MEF2A* in the E_349-activated SK-MEL-2-VP64 cells (n = 3 samples). **c** Plate clone formation assay result of the E_349-inhibited SK-MEL-2-KRAB cells (n = 3 samples). **d** CCK8 cell proliferation assay results of the E_349-inhibited SK-MEL-2 cells at 24, 48, and 72 h (n = 4 samples). CI denotes CRISPR interference, and CA denotes CRISPR activation. All the data are expressed as the means ± standard deviations (SD) and analyzed by an unpaired two-tailed Student’s *t*-test: *, p < 0.05, **, p < 0.01, ***, p < 0.001, ****, p < 0.0001.

**Fig. 5 | Inhibition of E_156 contributes to the proliferation of SK-MEL-2 melanoma cells by downregulating *PTEN*. a** RT-qPCR result for *PTEN* in the E_156-inhibited SK-MEL-2-KRAB cells (n = 3 samples). **b** RT-qPCR result for *PTEN* in the E_156-activated SK-MEL-2-VP64 cells (n = 3 samples). **c** Plate clone formation assay result of the E_156-inhibited SK-MEL-2-KRAB cells (n = 3 samples). **d** CCK8 cell proliferation assay results of the E_156-inhibited SK-MEL-2 cells at 24, 48, and 72 h (n = 4 samples). CI denotes CRISPR interference, and CA denotes CRISPR activation. All the data are expressed as the means ± standard deviations (SD) and analyzed by an unpaired two-tailed Student’s *t*-test: *, p < 0.05, **, p < 0.01, ***, p < 0.001, ****, p < 0.0001.

o       (Fig. 5 and Fig. 6) The assays for two enhancers (E_349 and E_156) did support the conclusion that they were involved in tumor cell proliferation and the expression of the adjacent tumor suppressors. But those assays were not enough to show they were important for tumorigenesis and development. Authors need to perform in vivo experiments, if possible in immune proficient hosts given the known biology of melanoma, to test if these enhancers are really functional.

***Response*:** Thank you for your constructive and rigorous comments. In response to the reviewer's suggestion, we initially aimed to validate the function of E_349 and E_156 in vivo using 3 or 5-week-old female C57BL/6 mice (immune proficient hosts) in a cell-derived xenograft (CDX) experiment. However, despite numerous attempts, including increasing the number of cells injected subcutaneously and altering the injection location (armpit, groin, etc.), we observed that the A375-derived melanoma tumors did not grow well. As an alternate approach, we used immunodeficient nude mice to investigate the roles of E_349 and E_156 in melanoma growth. Enhancer-inhibited A375-KRAB and A375-KRAB infected with the empty virus melanoma cells were used for CDX. 1×10^6^ viable cells were resuspended in 100 µl DMEM and subcutaneously injected into each 5-week-old female nude mice (C57BL/6 background), and then detected the tumor growth including volume and weight at different times (**Fig. 1 and 2 below**). As a result, the subcutaneous A375 melanoma cells in the E_349-inhibited group were significantly larger (p-value < 0.05 at 2 weeks, and p-value < 0.01 at 3 weeks) (**Fig. 1a and Fig. 1b, left below**) and heavier (p-value < 0.01) (**Fig. 1b, right below**) than those in the control group, highlighting the important function of E_349 in melanoma progression; the subcutaneous A375 melanoma cells in the E_156-inhibited group were markedly larger (p-value < 0.05 at 2 and 3 weeks) (**Fig. 2a and Fig. 2b, left below**) and heavier (p-value < 0.01) (**Fig. 2b, right below**) than those in the control group, highlighting the important function of E_156 in melanoma progression. Taken together, above data indicate that E_349 and E_156 are *bona fide* functional enhancers in melanoma growth and progression. **We have added the results into Fig. 5l, m and Fig. 6j, k, the related description (L376-380, L446-450), and figure legend (L1354-1358, and L1382-1385) in the revised manuscript, and please check our revised manuscript.**

**Fig. 1 | Evaluate the effect of E_349 inhibition on the proliferation potential of melanoma cells by using the CDX model**. A375-KRAB infected with the empty virus and E_349-inhibited A375-KRAB melanoma cells were used for CDX. 1×10^6^ viable cells were resuspended in 100 µl DMEM and subcutaneously injected into 5-week-old female nude mice (C57BL/6 background) and then detected the tumor growth including volume and weight. The representative three-dimension (3D) modeling analysis process (**a, left**) and statistical results (**a, right**) in calculating A375-derived tumor volumes at two and three weeks after subcutaneous injection, and then the tumors were weighed (**b, right**) after euthanasia of the mice (**b, left**). CI denotes CRISPR interference. All the data are expressed as the means ± standard error of mean (SEM) (n = 7 samples) and p-values were calculated by an unpaired two-tailed Student’s *t*-test: *, p < 0.05, **, p < 0.01, ***, p < 0.001, ****, p < 0.0001.

**Fig. 2 | Evaluate the effect of E_156 inhibition on the proliferation potential of melanoma cells by using the CDX model**. A375-KRAB infected with the empty virus and E_156-inhibited A375-KRAB melanoma cells were used for CDX. 1×10^6^ viable cells were resuspended in 100 µl DMEM and subcutaneously injected into 5-week-old female nude mice (C57BL/6 background) and then detected the tumor growth including volume and weight. The representative three-dimension (3D) modeling analysis process (**a, left**) and statistical results (**a, right**) in calculating A375-derived tumor volumes at two and three weeks after subcutaneous injection, and then the tumors were weighed (**b, right**) after euthanasia of the mice (**b, left**). CI denotes CRISPR interference. All the data are expressed as the means ± standard error of mean (SEM) (n = 7 samples) and p-values were calculated by an unpaired two-tailed Student’s *t*-test: *, p < 0.05, **, p < 0.01, ***, p < 0.001, ****, p < 0.0001.

*       In addition, the authors did not perform any analysis or experiment to suggest that the enhancers identified in melanoma cells were compromised, as compared with normal physiological condition, by somatic mutations. Histopathological staining of the patient samples where Del-HRR occurs to show that gene function and phenotype was impacted could be very helpful.

***Response*:** Thank you for the constructive comments and specific experimental suggestions. In response to the reviewer's suggestion, to explore the function of E_349 and E_156 in melanoma patients, we collected paraffin-embedded melanoma samples and DNA from 122 patients from the tissue sample library of Tianjin Cancer Hospital. We extracted the genomes and amplified the DNA sequence encompassing the core region of E349 for Sanger sequencing. Among the 122 melanomas, we only identified 2 samples with deletion or extensive mutations in E_349 (**Fig. 1** **below**). The immunohistochemistry (IHC) results indicated that E_349 with deletion or extensive mutations markedly reduced the MEF2A expression and enhanced melanoma growth (**Fig. 2** **below**), underscoring the importance of E_349 in melanoma development. Regrettably, due to the rarity of samples among Asian populations, we only obtained a small number of melanoma patient samples and did not identify any with deletions or extensive mutations in E_156. Therefore, to fully address this request regarding E_156, we need more time to collect and identify sufficient melanoma patient samples. However, given the established tumor suppressor role of PTEN in melanoma and its clear chromatin interaction with E_156, we can infer the important role of E_156 in melanoma development and survival. For all these patient-level results, please refer to our revised manuscript. **We have added the results into Fig. S12, the related description (L380-387, L450-452), and figure legend (L1530-1536) in the revised manuscript, and please check our revised manuscript.**

**Fig. 1 | Two melanoma samples with E_349 deletion or extensive mutations.** We collected patient melanoma tissues, extracted the genomes, and amplified the DNA sequence harboring E349 core region for Sanger sequencing. One sample was detected with E_309 deletion (Patient 1) (**a**) and the other sample with extensive mutations (patient 2) (**b**).

**Fig. 2 | Immunohistochemistry (IHC) results of melanoma patient samples with E_349 deletion or extensive mutations**. We collected patient melanoma tissues, extracted the genomes, and amplified the DNA sequence harboring E349 core region for Sanger sequencing. One sample was detected with E_349 deletion (Patient 1) and the other sample with extensive mutations (patient 2). Three samples with similar disease courses were used as random controls. These samples were performed IHC with MEF2A and Ki-67 antibodies.

*       More clarity is needed on how gene targets for some of the enhancers were identified. This is critical.

***Response*:** Thanks for the raised concern. As the reviewer mentioned, it is critical to accurately locate the target genes of enhancers. We have addressed relative questions as below and revised our manuscript.

o       Figure 1d) How were enhancers-promoter associations identified?

***Response*:** Thank you for your insightful question. As the reviewer pointed out, accurately identifying the target genes of enhancers is indeed crucial. In Fig. 1 of our previous manuscript, we did not mention the target genes of the enhancers, nor did we identify the interactions between the enhancers and the promoters. In this section, we mainly conducted a global detection of high recurrence regions (HRRs). While functional enhancers are our primary area of interest, through the annotation of HRR regions, we discovered that numerous genes, such as *TERT*, *CDKN2A*, and other novel genes, also hold potential for cancer driver studies. The genes shown in the figure are those disrupted by highly recurrent variations. We apologize for any confusion caused, and we have revised the corresponding figure legend to clarify the evidence source.

o       Figure 2a) Using proximity of enhancer to nearest gene is not an acceptable metric for enhancer-promoter interactions. It is known that only ~7% of human enhancers are localized to their nearest gene. What are the results from running multiple random iterations based on random intergenic regions?

***Response*:** Thank you for your insightful question. As the reviewer pointed out, accurately identifying the target genes of enhancers is indeed crucial. We apologize for previously characterizing the enhancer targets based on the nearest gene concept. In this section, we have redefined the presumed targets using all genes within 1Mb downstream and upstream of enhancers prior to generating HiChIP loops. Furthermore, we divided the presumed targets into five groups based on the HRR types of their enhancers. Similarly, we performed a Mann-Whitney U Test on the presumed genes in HRR-associated enhancers across the five HRR type groups and those in HRR-unrelated enhancer groups. As shown in Fig. 3 of our revised manuscript, the presumed targets show significant screen scores in the DEL, DUP, and SNV groups (p-value (DEL) = 0.0033; p-value (DUP) = 0.0028; p-value (SNV) = 0.0076). All these results have been updated to **Fig. 2a** of our revised manuscript.

o       Figure 2b, c) How were enhancer-promoter interactions identified. If proximity was used, this can be problematic for interpreting downstream results. In addition, how do the results on E-P interactions from A375 capitulate in other melanoma cell lines? Similar question on heterogeneity in these 3D looping patterns.

***Response*:** Thank you for your insightful question. In Fig. 2b, we propose a strategy for building a limited enhancer screening library. Considering the large number of all HRR-associated enhancers, it's not feasible to screen all of them due to inefficiencies and high costs. As an alternative, we established a filter to narrow down the enhancer candidates. We collected a series of melanoma-essential genes that have been reported and/or screened in other melanoma studies, and created a list of melanoma-essential genes (see Supplementary Table 4). All HRR-associated enhancers located within 1Mb of these essential genes were considered our screening candidates, including both proximal and relatively distal enhancers. In original Fig. 2c, we displayed the designed and nearest genes in the results. Our original intention was to provide more information about enhancers, not to establish the association between enhancers and genes, as there is no direct evidence for such a connection. To avoid misunderstanding, we have removed such information about the nearest gene in new **Fig. 2c**. Besides, to identify the interaction of the screened enhancers in other melanoma cell lines, we gathered HiChIP loop data for WM (GEO: GSM5680738 [11]) and COLO (GEO: GSM5680737 [11]) cell lines. Among the top three enhancers, the E_349-*MEF2A* interaction only exists in A375 melanoma cells, E_156-*ATAD1*/*KLLN*/*PTEN* is present in both A375 melanoma cells and WM, and E_409-*NF1*/*OMG*/*EVI2B*/*EVI2A* is present in all three melanoma cell lines (**Table 1 below**). Additionally, we acknowledge the heterogeneity in these 3D looping patterns. We have briefly discussed this limitation (**L487-494**) in our revised manuscript.

o       Figure 3c) Similar to the above comments for Figure2, how were enhancer-promoter interactions identified. Also, does the finding in A375 cells capitulate in other melanoma samples?

***Response*:** Thank you for your insightful question. In Fig. 3c, we only displayed the epigenomic features of the 66 screened HRR-associated enhancer regions. The promoter signal (H3K4me3) that we detected in the enhancer regions serves to indicate the possibility of promoters acting like enhancers. Additionally, we have briefly addressed the issue of epigenomic heterogeneity (**L257-271**) in our revised manuscript.

o       Figure 3e) Once again, it is a major problem using proximity to annotate enhancer elements. This is helped by Figure4 and the HiChIP analysis, however, this should have been used in Figure1 for initial enhancer-promoter interactions. In addition, HiChIP-seq should be performed in at least one more representative melanoma sample.

***Response*:** Thank you for your insightful comment. We apologize for any confusion caused by the gene annotation problem. Previously, until the HiChIP analysis in Fig. 4, the genes we marked in Fig. 2 and Fig. 3 were artificially designated genes used to filter the enhancer library. In this revised manuscript, we have moved the HiChIP analysis to Fig. 2, replacing the original "Nearest gene" annotation with a target gene circus plot to provide a clearer understanding for the readers. Additionally, we have further clarified the specific referential information in the legend of Fig. 1. Certainly, to investigate the interaction of the selected enhancers across different melanoma cell lines, we gathered HiChIP loop data for WM and COLO cell lines [11]. The E_349-*MEF2A* interaction is unique to A375 cells, E_156-*ATAD1*/*KLLN*/*PTEN* is found in both A375 and WM cells, and E_409-*NF1*/*OMG*/*EVI2B*/*EVI2A* is common to all three cell lines (**Table 1 below**). These results suggest that the function of enhancers may be dependent on specific tumor environments. Additional results can be found in Supplementary Table 13 of the revised manuscript. Please also check our revised manuscript.

**Table 1. Enhancer targets analysis based on HiChIP of WM and COLO cell lines.**

| **id** | **p-value** | **variant** | **Target-gene (A375)** | **Target-gene (WM)** | **Target-gene (COLO)** |
| --- | --- | --- | --- | --- | --- |
| E_349 | 4.92E-06 | DEL | *LRRC28*, *MEF2A* | *AC022819.1*, *AC022819.2*, *HNRNPA1P62*, *HSP90B2P*, *LRRC28*, *RP11-20G13.1*, *RP11-20G13.2*, *RP11-20G13.3*, *RP11-616M17.1*, *TTC23* | *AC022819.1*, *AC022819.2*, *HSP90B2P*, *LRRC28*, *RP11-20G13.1*, *RP11-20G13.2*, *RP11-20G13.3*, *RP11-616M17.1*, *TTC23* |
| E_156 | 4.92E-06 | DEL | *ATAD1*,  *KLLN*,  *PTEN* | *ATAD1*, *CFL1P1*, *KLLN*, *PTEN*, *RP11-380G5.2*, *RP11-380G5.3*, *SNORD74* |  |
| E_409 | 4.92E-06 | DEL | *NF1*, *OMG*, *EVI2B*, *EVI2A*, *RAB11FIP4* | *CTD-2370N5.3*, *DPRXP4*, *EVI2A*, *EVI2B*, *MIR4733*, *NF1*, *OMG*, *RNF135*, *RP11-271K11.5*, *RP11-848P1.2*, *RP11-848P1.3*, *RP11-848P1.4*, *RP11-848P1.5*, *RP11-848P1.9* | *CTD-2370N5.3*, *EVI2A*, *EVI2B*, *NF1*, *OMG* |

o       Figure 4a) It is unclear if all the HRR enhancers contain a valid enhancer-promoter interaction. How many of the 66 HRR enhancers were interacting with the predicted gene?

***Response*:** Thank you for your insightful question. We apologize for not explaining this clearly before. In our study, 57 out of 66 HRR enhancers interact with at least one target gene, and 46 enhancers have multiple target genes. Among the 11 enhancers, the predicted target genes align with the designated melanoma essential genes.

o       Figure 5a) Why was MEF2A not identified in the initial analysis. Is this due to the enhancer-promoter predictions used throughout the study? This is again concerning as E_349 was the top hit of the screen. In addition, why/how was MEF2A chosen to functionalize?

***Response*:** Thank you for your insightful question and we apologize for any confusion. It's important to clarify that we assembled a list of melanoma essential genes that were reported and/or screened in other melanoma studies. Unlike *PTEN*, a well-known tumor suppressor, the role of *MEF2A* in melanoma hasn't been clearly defined, which is why *MEF2A* wasn't initially designated as an essential gene when we designed the enhancer library. In fact, in our initial HRR detection analysis, we found many deletion events disrupting *MEF2A*, which underscores the potential influence of MEF2A dysregulation on melanoma. Also, the most significantly screened enhancer, E_349, loops to the promoter of *MEF2A*, not its designed target, *IGF1R*. Moreover, we found evidence supporting the tumor suppressor features of *MEF2A* through the analysis of public data sources like TCGA and CRISPR. Given the genetic, gene expression, and functional screening evidence, we believe that both E_349 and its corresponding target gene, *MEF2A*, are worthy of further functional verification. As anticipated, our functional experiments confirmed that inhibiting E_349 in melanoma cells doesn't affect the expression of its predicted target gene, *IGF1R* (refer to **Reviewer 2, Question 4 below**). This indicates that E_349 cannot regulate *IGF1R* expression in melanoma. In conclusion, along with the new experimental validation in this revision, we believe that the E_349-*MEF2A* interaction is functionally relevant for melanoma growth and progression. We have made it clear in the result and figure legend of our revised manuscript.

o       Figure 6c) Does this enhancer also interact with either KLLN or ATAD1? It seems this enhancer is interacting with the 3' end of these genes in Figure 6a. This should also be evaluated due to the low reduction of PTEN protein expression upon enhancer loss if Figure 6f.

***Response*:** Thanks for the good suggestions. As the reviewer mentioned, E_156 interacts with the 3' end of *KLLN* and *ATAD1* in Fig. 6a. According to the reviewer’s suggestion, to investigate if E_156 regulates *KLLN* and *ATAD1* expression, we detected the *KLLN* and *ATAD1* expression in E_156-inhibited A375-KRAB, SK-MEL-28-KRAB, and SK-MEL-2-KRAB cells. Consequently, RT-qPCR results indicated that both *KLLN* and *ATAD1* expression have no significant difference in the E_156-inhibited A375-KRAB, SK-MEL-28-KRAB, and SK-MEL-2-KRAB cells compared to their control cells (**Fig. 1 below**). In addition, western blotting results indicated that either KLLN or ATAD1 expression has no significant difference in the E_156-inhibited A375-KRAB cells compared to the control cells (**Fig. 2 below**). Above results suggested that the E_156 cannot directly regulate the expression of *KLLN* or *ATAD1*. We have added the results into Fig. S10, the related description (L423-425), and figure legend (L1515-1523) in the revised manuscript, and please check our revised manuscript.

**Fig. 1 | RT-qPCR results of *KLLN* and *ATAD1* in the E_156-inhibited A375-KRAB, SK-MEL-28-KRAB, and SK-MEL-2-KRAB cells compared to their control cells. a** RT-qPCR result of *KLLN* expression in the E_156-inhibited A375-KRAB, SK-MEL-28-KRAB, and SK-MEL-2-KRAB cells. **b** RT-qPCR result of *ATAD1* expression in the E_156-inhibited A375-KRAB cells, SK-MEL-28-KRAB, and SK-MEL-2-KRAB cells. CI denotes CRISPR interference. All the data are expressed as the means ± standard deviations (SD) (n = 3 samples) and analyzed by an unpaired two-tailed Student’s *t*-test. “ns” represents no significant difference between the indicated experimental groups.

**Fig. 2 | Western blotting results of KLLN and ATAD1 in the E_156-inhibited A375-KRAB cells compared to the control cells. a** Representative (top graph) and statistical (bottom graph) results of KLLN expression in the E_156-inhibited A375-KRAB cells. **b** Representative (top graph) and statistical (bottom graph) results of ATAD1 expression in the E_156-inhibited A375-KRAB cells. CI denotes CRISPR interference. All the data are expressed as the means ± SD (n = 3 samples) and analyzed by an unpaired two-tailed Student’s *t*-test. “ns” represents no significant difference between the indicated experimental groups.

o       In addition, how many valid interactions were identified in the HiChIP data? It is unclear from the methods.

***Response*:** Thanks for the good question and comment. Our HiChIP identified a total of 121,775 loops, of which 68,998 loops contain valid Enhancer-Promoter pairs. Enhancers are A375 total enhancers which defined by A375 H3K27ac, H3K4me1, and ATAC-seq, and promoters of the genes are defined as 3kb near the transcriptional start site (TSS). We have made it clear in the method section (**L728-731**).

o       Figure 5) The results for MEF2A are interesting, however, it is extremely problematic that E_349 is the top enhancer and not associating with the predicted gene(s).

***Response*:** Thank you for your insightful question. Our primary goal for screening was to illuminate enhancers that regulate known classic genes in melanoma (such as *PTEN*), and to attempt to unravel the mechanisms by which these enhancers drive tumor development via these classic genes. Our screening results revealed that only a subset of enhancers fit this mechanistic profile. However, the functional role of the enhancers not targeting classic genes in melanoma development also warrants investigation. In particular, we found that the top enhancer, E_349, targets *MEF2A*, which may have a significant role in melanoma development. Although its role in melanoma is not yet elucidated, *MEF2A* has been demonstrated to be a noteworthy driver gene through genetic, functional screens, gene expression, and clinical studies. Moreover, our biological functional experiments confirmed that the inhibition of E_349 in melanoma cells doesn't affect the expression of its predicted target gene, *IGF1R*, indicating that E_349 doesn't regulate *IGF1R* expression in melanoma. We have made it clear in the result (**L354-356**) and figure legend (**Fig. S10**) of our revised manuscript.

*       Figure 1c) It is unclear how the mutation calls performed here compared to their original study (i.e., number of mutation calls, type of call, location of call, should be shown in supplemental information).

***Response*:** Thank you for your insightful question and for bringing attention to the confusion surrounding our previous mutation calling procedures. In our study, we handled the calling of structural variations (SVs) ourselves. For small somatic mutations, which include single-nucleotide variants (SNVs) and insertions/deletions (INDELs), we directly extracted the Genome Analysis Toolkit (GATK) Mutect2 call sets of MELA-AU and SKCM-US from the International Cancer Genome Consortium (ICGC). However, we supplemented the SKCA-BR call sets with GATK Mutect2. Regarding the structural variations (SVs), the original call sets had issues, such as a small number of variants and incomplete types. To address these issues, we re-analyzed the data using Manta [12] and GRIDSS [13], and then merged the results from these two tools. This uniform analysis of SVs made the call sets compatible and comparable (**Fig. 1 below**). Upon comparison, we found that both Manta and GRIDSS callings could generate more SVs than the original calling, which used different tools. We have clarified this in the Methods section (**L563-567**) of our revised manuscript.

**Fig. 1| Comparison of SV call sets between origin and Manta/GRIDSS.** The number of each SV call sets across three melanoma projects were stat in the table, origin call sets were directly downloaded from ICGC.

*       Figure S4a-c) Is the SK-MEL-5 mutational landscape, including intergenic regions, the same as A375?

***Response*:** Thank you for your insightful question. We did not use SK-MEL-5 melanoma cells for functional verification in Fig. S4a-c, and we wonder if the reviewer might be referring to SK-MEL-28 melanoma cells, not SK-MEL-5 melanoma cells. In response to your suggestion, we compared the mutation landscape among SK-MEL-28, A375, and other melanoma cells. We analyzed the Copy Number Variations (CNV) and Structural Variations (SV) statuses of 56 melanoma cell lines from the CCLE dataset. The results showed that 31 of the 66 functional enhancer signals from CRISPRi screen, share the same genetic alteration in both A375 and SK-MEL-28 melanoma cells. Interestingly, the enhancer we focused on, E_349, exhibits copy number gain in both melanoma cell lines. However, E_156, another enhancer, shows copy number gain in A375 melanoma cells and heterogeneous copy number loss (LOH) in SK-MEL-28 melanoma cells. Intriguingly, LOH in the E_156 region occurs in half of the melanoma cell lines. This widespread loss-of-function event of E_156 may also be associated with melanoma development. These results have been added to Supplementary Table 11 in our revised manuscript.

*       Figure 3d) It is unclear how motifs were identified and what background used for analysis. Notably, it is surprising TF such as MITF were not identified? ChIP-seq for at least one TF should be performed to demonstrate direct overlap with the HRR enhancers.

***Response*:** Thank you for your valuable comment. Following your suggestion, we gathered ChIP-seq narrow peak data for 13 transcription factors (TFs) from public datasets, and analyzed their signals in the 66 enhancer regions. The ChIP-seq results suggest that MITF may not be associated with these enhancers (**Fig. 1 below**). Consistent with the speculation in Fig. 3d, we observed HEXIM1 binding signals in 27 enhancer regions, including E_349 and E_156. We also found that AP2A, AR, and EGFR binding signals are present on many enhancers, which are specifically found in A375 melanoma cells, but are not enriched at the tissue level as per Fig. 3d. All TF signal profiles have been added to Supplementary Table 8 in our revised manuscript.

**Fig 1. ChIP-seq signal of TFs at the functional enhancer regions.** the upset plot represents the distribution of 13 TF ChIP-seq signals on the functional enhancers, the bar plot shows driver mutation types, and CRISPRi screen p-values of each enhancer.

*       Figure 6f) The reduction of PTEN expression upon enhancer loss is not striking. Does this reach significance?

***Response*:** Thanks for the concern and reminding. We are sorry for our carelessness. In this experiment, we used three samples and carried out data statistics. We showed the result of one sample (Fig. 6f, right). From the statistics of data results (Figure 6f, left), inhibition of enhancer significantly reduced the expression of PTEN. In order to show our results more clearly and rigorously, we have updated the most representative result (**Fig. 1**) of these three samples with significant difference in our revised manuscript. Thanks again for the reminding.

**Fig. 1 | Western blotting results of *MEF2A* protein expression in the E_156 (E_155 or E_154 adjacent to E_156)-inhibited A375-KRAB cells.** CI denotes CRISPR interference. Left graph is the representative result, and right graph is the statistical result. All the data are expressed as the means ± SD (n = 3 samples) and analyzed by an unpaired two-tailed Student’s *t*-test. Asterisks indicate significant differences between the indicated experimental groups: *, p < 0.05; **, p < 0.01; ***, p < 0.001; ****, p < 0.0001. **Reviewer #2:** In this manuscript, Luo et al., first computationally identified highly recurrent regions (HRRs) in the non-coding genome of melanoma based on WGS data of a large of cohort of human melanoma samples. They further carried out a CRISPRi tiling screen of putative enhancers in these HRRs using cell growth of A375 melanoma cells as a readout. After identifying 66 enhancers with putative tumor suppressive roles from the screen, they used the H3K27ac HiChIP approach to identify target genes of these enhancers in A375 cells. They further examined the functions of two enhancers that targeting MEF2A and PTEN, respectively, in melanoma cell proliferation, apoptosis and drug resistance. Overall, this is a very interesting and comprehensive study. It has revealed important information on enhancer-mediated transcriptional dysregulation in melanoma carcinogenesis. I have only several minor comments.

***Response*:** Thanks for the reviewer’s highlight and positive response, and we also greatly appreciate the reviewer's summarization of the scientific findings revealed in our study.

1.      It appears that enhancer annotation was based on H3K27Ac ChIP data from multiple melanoma cell lines, beyond A375 and SK-Mel-28 cells that most experiments were carried out in. How heterogenous were the data for various melanoma cell lines, in terms of enhancer calling? The authors may discuss this in the manuscript.

***Response*:** Thank you for your constructive comments. Indeed, in this study, we broadly defined the enhancer region using the H3K27ac signal from different melanoma cell lines. However, when designing the screening library, to ensure accurate sgRNA targeting in the A375 cells we used for screening, we precisely defined the enhancer region based on the comprehensive epigenomic data (including H3K27ac, H3K4me1, and open chromatin) of A375 melanoma cells. To illustrate the heterogeneity of enhancers among different melanoma cell types, we leveraged the H3K27ac signal and calculated the presence of the 66 significant HRR-associated enhancers identified from CRISPRi screening in various melanoma cell lines. The results suggest that at least half of these enhancers are present across different melanoma cell lines (**Table 1 below**). This finding has been added to Supplementary Table 9-10 in our revised manuscript. Additionally, **we have briefly addressed the issue of epigenomic heterogeneity (L487-494) in our revised manuscript**.

**Table 1. The presence of functional screened enhancers in different melanoma cell lines.**

| **Cell line** | **Existed functional enhancers** |
| --- | --- |
| SK-MEL-2 | 51 |
| MDA-MB-435 | 31 |
| SK-MEL-28 | 47 |
| MALME-3M | 30 |
| UACC-62 | 42 |
| SK-MEL-5 | 40 |
| UACC-257 | 43 |
| M14 | 45 |
| LOX-IMVI | 34 |

2.      Fig. 4d: It is unclear what the percentage of target genes showed up as hits in the CRISPR screen is? The authors may want to tune down the conclusion if only a small percentage of them showed up in the CRISPR screen hit list.

***Response*:** Thank you for your insightful question. In response to the reviewer's suggestion, we have calculated the percentage of target genes that appeared as hits in the CRISPRi screen. Our results reveal that 24 out of 200 target genes appeared in the public CRISPR positive selection screen (Fig. 4f), and 37 appeared in the public CRISPR negative selection screen. Thus, the presence of a quarter of these targets in the public hits at least indicates the potential of the screened enhancers and their targets to function as factors in melanoma growth and progression. We have also refined the related sentence in the revised manuscript.

3.      In general, I suggest the authors need to be cautious in making conclusion regarding the role of target genes (derived from HiChIP data) in melanoma carcinogenesis, as they are not experimentally verified (except for MEF2A and PTEN) and mostly just speculations at this stage. Chromatin interaction by itself does not dedicate functional relevance in the context of carcinogenesis.

***Response*:** Thank you for your professional comments and reminders. We agree with your assessment that our previous statement may have been biased and not sufficiently rigorous. To ensure the precision and correctness of our description, we have made careful modifications and toned down the related statements in our revised manuscript.

4.      Fig. 5h-J: IGF1R expression could be included as a control to confirm that IGF1R is not a target as the authors suggested.

***Response*:** Thanks for the rigorous comments. According to the reviewer’s suggestion, we detected the *IGF1R* expression in the E_349-inhibited A375-KRAB, SK-MEL-28-KRAB, and SK-MEL-2-KRAB melanoma cells, respectively. Consequently, RT-qPCR results indicated that *IGF1R* expression has no significant difference in the E_349-inhibited A375-KRAB, SK-MEL-28-KRAB, and SK-MEL-2-KRAB cells compared to the control cells (**Fig. 1 below**). In addition, western blotting result indicated that IGF1R expression has no marked difference in the E_349-inhibited A375-KRAB cells compared to the control cells (**Fig. 2 below**). Above results suggested that *IGF1R* is not the E_349 targeted gene and cannot be directly regulated by E_349. We have added the results into Fig. S10, the related description (L354-356), and figure legend (L1515-1523) in the revised manuscript, and please check our revised manuscript.

**Fig. 1 | RT-qPCR results of *IGF1R* in the E_349-inhibited A375-KRAB, SK-MEL-28-KRAB, and SK-MEL-2-KRAB cells compared to the control cells. a** RT-qPCR result of *IGF1R* expression in the E_349-inhibited A375-KRAB cells. **b** RT-qPCR result of *IGF1R* expression in the E_349-inhibited SK-MEL-28-KRAB cells. **c** RT-qPCR result of *IGF1R* expression in the E_349-inhibited SK-MEL-2-KRAB cells. CI denotes CRISPR interference. All the data are expressed as the means ± SD (n = 3 samples) and analyzed by an unpaired two-tailed Student’s *t*-test. “ns” represents no significant difference between the indicated experimental groups.

**Fig. 2 | Western blotting result in the E_349-inhibited A375-KRAB cells compared to the control cells. a** Representative graph of IGF1R expression in the E_349-inhibited A375-KRAB cells. **b** Statistical result of IGF1R expression in the E_349-inhibited A375-KRAB cells. CI denotes CRISPR interference. Data are expressed as the means ± SD (n = 3 samples) and analyzed by an unpaired two-tailed Student’s *t*-test. “ns” represents no significant difference between the indicated experimental groups.

5.      Fig. 5j/k (and related supplementary figures): Since manipulation of E349 affected readouts under both control and vemurafenib treatment conditions, to examine the role of E349 in vemurafenib resistance, the authors should analyze the data in a different way, i.e. calculating the ratios between treatment versus control in different groups of cells. Same suggestion for similar figures in Fig. 6 regarding the PTEN enhancers.

***Response*:** Thanks for the good comments. According to the reviewer’s suggestion, we conducted a new statistical analysis of the relevant data in accordance with the reviewer’s suggestions. As a result, despite certain trends, it has no significant difference between the treatment or without treatment (**Fig. 1 below**), suggesting this may due to inappropriate experimental processing conditions, such as drug concentration and duration of treatment. Actually, our study mainly focused on the effect of enhancers on melanoma growth. In our opinion, since inhibition of enhancers, E_349 and E_156, promote the growth of melanoma, it also has a certain promoting effect under drug pressure. In other words, we further used vemurafenib to validate the growth phenotypes and did not focus on drug resistance itself. In order to better highlight the main theme of our study, we adopted this method of presenting the results. Thanks again for your understanding, and valuable and professional suggestion.

**Fig. 1 | Re-analyzed the data associated with vemurafenib treatment. a** corresponding to Fig. 5j. **b** corresponding to Fig. 5k. **c** corresponding to Fig. 6h. **d** corresponding to Fig. 6i. Data are expressed as the means ± SD (n = 3 samples) and analyzed by an unpaired two-tailed Student’s *t*-test. “ns” represents no significant difference between the indicated experimental groups. Asterisks indicate significant differences between the indicated experimental groups: *, p < 0.05; **, p < 0.01; ***, p < 0.001; ****, p < 0.0001.

**Reference:**

1.	Terranova CJ, Tang M, Maitituoheti M, Raman AT, Ghosh AK, Schulz J, Amin SB, Orouji E, Tomczak K, Sarkar S, et al: **Reprogramming of bivalent chromatin states in NRAS mutant melanoma suggests PRC2 inhibition as a therapeutic strategy.** *Cell Rep* 2021, **36:**109410.

2.	Terranova C, Tang M, Maitituoheti M, Raman AT, Schulz J, Amin SB, Orouji E, Tomczak K, Sarkar S, Oba J, et al: **Bivalent and Broad Chromatin Domains Regulate Pro-metastatic Drivers in Melanoma.** *bioRxiv* 2019**:**721480.

3.	Corces MR, Granja JM, Shams S, Louie BH, Seoane JA, Zhou W, Silva TC, Groeneveld C, Wong CK, Cho SW, et al: **The chromatin accessibility landscape of primary human cancers.** *Science* 2018, **362**.

4.	Thorvaldsdottir H, Robinson JT, Mesirov JP: **Integrative Genomics Viewer (IGV): high-performance genomics data visualization and exploration.** *Brief Bioinform* 2013, **14:**178-192.

5.	Li W, Xu H, Xiao T, Cong L, Love MI, Zhang F, Irizarry RA, Liu JS, Brown M, Liu XS: **MAGeCK enables robust identification of essential genes from genome-scale CRISPR/Cas9 knockout screens.** *Genome Biol* 2014, **15:**554.

6.	Zhang X, Yang L, Szeto P, Abali GK, Zhang Y, Kulkarni A, Amarasinghe K, Li J, Vergara IA, Molania R, et al: **The Hippo pathway oncoprotein YAP promotes melanoma cell invasion and spontaneous metastasis.** *Oncogene* 2020, **39:**5267-5281.

7.	Palanisamy N, Ateeq B, Kalyana-Sundaram S, Pflueger D, Ramnarayanan K, Shankar S, Han B, Cao Q, Cao X, Suleman K, et al: **Rearrangements of the RAF kinase pathway in prostate cancer, gastric cancer and melanoma.** *Nat Med* 2010, **16:**793-798.

8.	Zhao K, Zhou Y, Wu C, Wang J, Yao H, Cheng X, Zhao L, Wang W, Chu X, Yi X, et al: **Endogenous fine-mapping of functional regulatory elements in complex genetic loci.** *bioRxiv* 2023**:**2023.2005.2006.539696.

9.	Tang Z, Kang B, Li C, Chen T, Zhang Z: **GEPIA2: an enhanced web server for large-scale expression profiling and interactive analysis.** *Nucleic Acids Res* 2019, **47:**W556-w560.

10.	Ghandi M, Huang FW, Jane-Valbuena J, Kryukov GV, Lo CC, McDonald ER, 3rd, Barretina J, Gelfand ET, Bielski CM, Li H, et al: **Next-generation characterization of the Cancer Cell Line Encyclopedia.** *Nature* 2019, **569:**503-508.

11.	Donohue LKH, Guo MG, Zhao Y, Jung N, Bussat RT, Kim DS, Neela PH, Kellman LN, Garcia OS, Meyers RM, et al: **A cis-regulatory lexicon of DNA motif combinations mediating cell-type-specific gene regulation.** *Cell Genom* 2022, **2**.

12.	Chen X, Schulz-Trieglaff O, Shaw R, Barnes B, Schlesinger F, Kallberg M, Cox AJ, Kruglyak S, Saunders CT: **Manta: rapid detection of structural variants and indels for germline and cancer sequencing applications.** *Bioinformatics* 2016, **32:**1220-1222.

13.	Cameron DL, Schroder J, Penington JS, Do H, Molania R, Dobrovic A, Speed TP, Papenfuss AT: **GRIDSS: sensitive and specific genomic rearrangement detection using positional de Bruijn graph assembly.** *Genome Res* 2017, **27:**2050-2060.

**Second round of review**

**Reviewer 1**

Authors have done a good job responding to my comments. I commend them for thorough and comprehensive responses to each of my points. This work provides important advance towards functionalization of enhancer elements in melanoma.

**Reviewer 2**

The authors have included substaintial amout of new data in the revised manuscript and addressed all my previous concerns.
